# The structural features and immunological role of biomphalysins in the snail *Biomphalaria glabrata*

**DOI:** 10.1371/journal.ppat.1013225

**Published:** 2025-06-24

**Authors:** Pierre Poteaux, Aline Parpinel, Chantal Ripoll, Amélie Sarrazin, Richard Galinier, Sabine Brugière, Yohann Couté, Lionel Mourey, Patrick C. Hanington, Benjamin Gourbal, Laurent Maveyraud, David Duval

**Affiliations:** 1 IHPE, CNRS, IFREMER, Université de Montpellier, Université Perpignan Via Domitia, Perpignan, France; 2 Institut de Pharmacologie et de Biologie Structurale (IPBS), Université de Toulouse, CNRS, Université Toulouse III - Paul Sabatier (UT3), Toulouse, France; 3 Institut de Génomique Fonctionnelle, Université de Montpellier, CNRS, INSERM, Montpellier, France; 4 MRI, BioCampus Montpellier, CNRS, INSERM, Université de Montpellier, Montpellier, France; 5 Université Grenoble Alpes, INSERM, CEA, UA13 BGE, CNRS, CEA, UAR2048, Grenoble, France; 6 School of Public Health, University of Alberta, Edmonton, Alberta, Canada; University of Wisconsin-Madison, UNITED STATES OF AMERICA

## Abstract

Biomphalysins are β-Pore Forming Toxins (β-PFT) identified in the planorbid *Biomphalaria glabrata* that belong to the aerolysin-like protein family. Despite potentially diverse biochemical activities, very few eukaryotic aerolysin-related proteins have been extensively studied. Most of the data refers to their discovery in genomes or to transcriptional activity. The involvement of biomphalysins in the immune response of *Biomphalaria glabrata* has been studied previously, especially regarding biomphalysin 1, which can bind and kill *Schistosoma mansoni* mother sporocysts. However, the repartition of biomphalysin 1 protein in *B. glabrata* has yet to be defined. The transcriptional behavior of the 22 other biomphalysin genes following immune challenge also remains uncharacterized. Therefore, herein, we investigate for the first time the tissular distribution of biomphalysin 1 (and 2) in *B. glabrata* by histological and cytological analyses through immunofluorescence approaches, notably unveiling unexpected tissue location that are involved in biomphalysin 1 synthesis. Structural predictions of the 23 members of the family have been updated using predictions based on aminoacyl spatial pair representation (AlphaFold2), highlighting unique features of the small lobe. In addition, mass spectrometry-based proteomic data more precisely predicted the regions of post-translational cleavage of biomphalysin 1. Transcriptional activity of the biomphalysin genes was explored, after which the plasmatic presence of the biomphalysin proteins was investigated in naive and *S. mansoni*-infected snails. The ability of native biomphalysin 1 (and 2) to bind several cell types was also investigated and correlated with the lytic ability of plasma toward the exposed cells, highlighting the central role occupied by biomphalysin 1 (and 2) in the humoral immunity of *B. glabrata*.

## Author summary

As an intermediate host for the intestinal schistosomiasis agent, *Schistosoma mansoni*, *Biomphalaria glabrata* immunity is extensively studied, as recommended by the World Health Organization. Biomphalysins are among the most important humoral factors in the snail hemolymph, and the main lytic plasma factors. Although this family of pore-forming toxins provides a better understanding of *B. glabrata* immunity, it should not overshadow the prospect of studying these proteins in the context of targeting other cell types, such as some cancer cell lines, for example. This study presents for the first time the distribution of the most abundant biomphalysin protein (biomphalysin 1/2) in the snail *Biomphalaria glabrata*. This protein is found in glandular structures of the foot, in other structures identified as hemolymphatic vessels, in plasma but also in a specific subpopulation of round hemocytes. This work also introduces the ability of biomphalysin 1/2 to bind several cell types with a specific spotted pattern and at a particular high-molecular weight, indicative of pore formation. Up-to-date structural prediction of biomphalysins are implemented using a trained artificial intelligence (AI) program, revealing, among other things, a potential lectin pattern and a post-translational cleavage (also confirmed by a peptide coverage obtained through a proteomic approach).

## Introduction

Pore Forming Toxins (PFTs) are a group of proteins that form either β-barrels or α-helix channels by assembling toxin monomers following binding to cell membranes [1]. They constitute the largest class of bacterial toxins, acting as virulence factors. Two families of PFT exist: the α-PFT such as haemolysin BL from *Bacillus cereus* [2] or colicins from *Escherichia coli* [3]; and the β-PFT as α- and γ-haemolysin from *Staphylococcus aureus* [4] or aerolysin from *Aeromonas hydrophila* [5]. Furthermore, PFTs are widely distributed in the tree of life through distinct eukaryotic taxa. Their polyphyletic distribution is assumed to originate from several bacterial Horizontal Gene Transfer events (HGT) [6–8]. However, except for the Cnidarian actinoporins [9–13], the genomic profiles, transcriptional patterns, and histological distribution of eukaryotic PFTs remain poorly understood, and the structures and functions of these proteins in eukaryotic animals is seldom explored. The lack of interest regarding PFTs in most eukaryotes likely stems from the unannounced way these toxins are often discovered, from studies such as global transcriptomic or genomic sequencing and annotation analyses. However, when PFTs have been afforded proper attention, a great variety of functions and important biological contexts are revealed [14–17].

In the snail *Biomphalaria glabrata*, 23 genes encode biomphalysins, which belong to the β-pore forming toxins family and to the sub-family of aerolysins. *B. glabrata* originated in South America, where it serves as the intermediate host of the human-infecting parasite *Schistosoma mansoni*. Evidence supports a bacterial origin of the biomphalysin family, suggesting that these genes were adapted to the organism *B. glabrata* [18]. As part of the aerolysin family, biomphalysins exhibit a predicted structure containing a small lobe and a large lobe, the latter constituted by the aerolysin motif [17,18].

The 23 biomphalysins are important constituents of the *B. glabrata* immune response. Some biomphalysins are found in the hemolymph as immune effectors that bind to several microorganisms [19,20] including sporocysts of *S. mansoni*. In synergy with plasmatic compounds, biomphalysin 1 (B1) can induce a cytolytic effect on *S. mansoni* sporocysts [17]. Moreover, not all genes coding for biomphalysins have the same transcriptional pattern, and not all snail tissues are transcriptionally active for all the biomphalysins [18]. Their mode of action is not yet known, even though some evidence suggests the involvement of molecular partners to induce their binding or activation [17,19].

Our previous work [17,18,20] on biomphalysins has mainly focused on their phylogenetic origin and defining the broad lines of their function. The present study aims to establish the location and the precise context of biomphalysin expression and synthesis within *B. glabrata* to define their function, based on cellular and histological-oriented experiments, focusing on B1 and its ability to bind to different cell types.

## Results

### Biomphalysin 1/2 exhibit specific high-molecular weights in hemocytes, mantle edge and posterior foot through Western blotting

**Fig 1:**
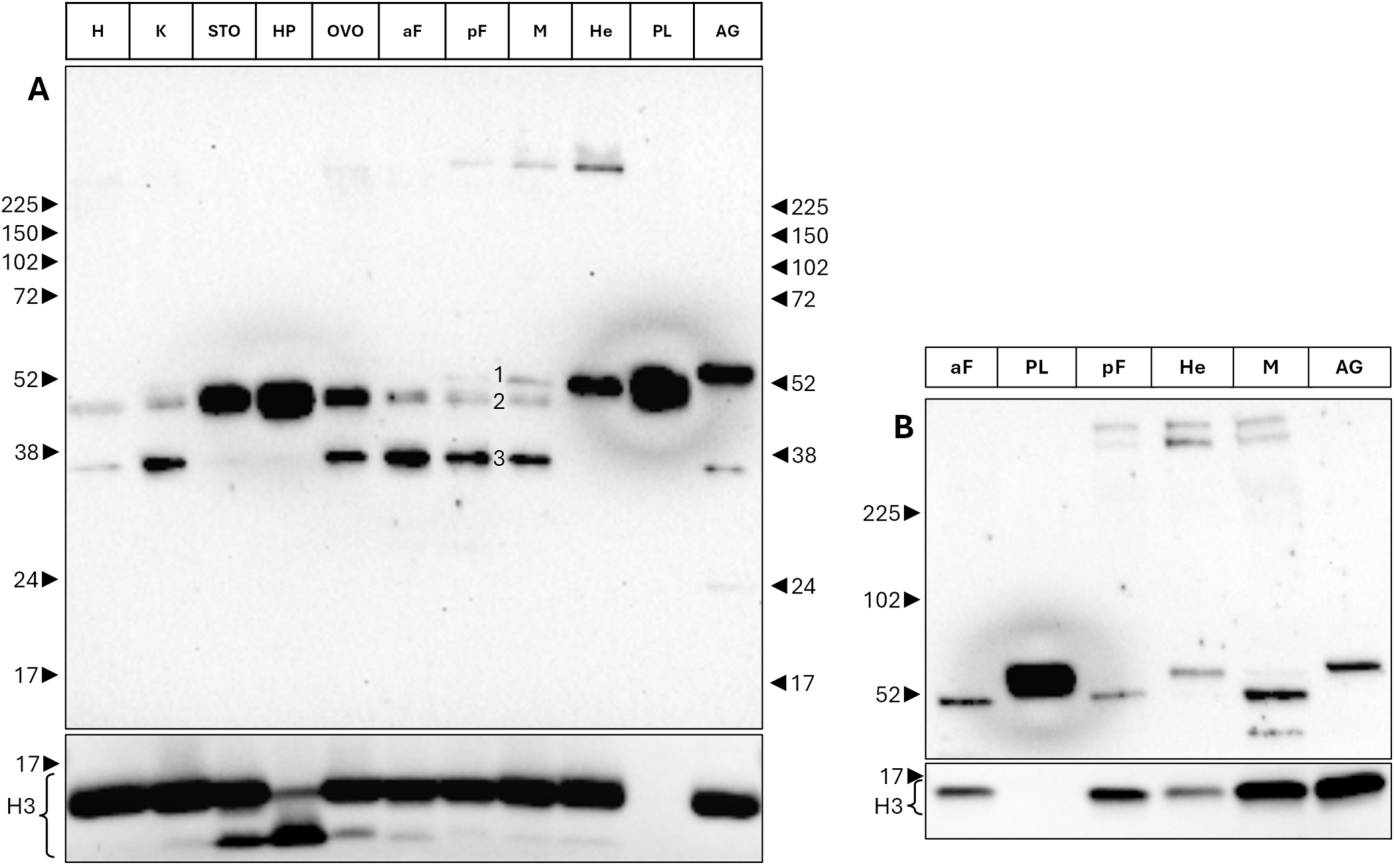
Biomphalysin 1 distribution in *Biomphalaria glabrata* tissues assessed through Western blotting. Tissues from five *Bg*BRE2 strain individuals were dissected, pooled and lysed. A protein dosage was performed, ensuring a homogeneous deposition of the samples on gels. SDS-PAGE were performed and biomphalysin 1 was revealed through immunoblotting. Biomphalysin 1 has an approximative size of 54 kDa (monomeric form), there is a slight difference in size between several tissues. Some tissues (hemocytes, mantle edge, posterior part of the foot, kidney -including tegument of the paleal cavity-) exhibit high molecular weight bands. aF: anterior part of the Foot, AG: Albumin Gland, H: Heart, He: Hemocytes, HP: Hepatopancreas, K: Kidney, M: Mantle edge, OVO: Ovotestis, pF: posterior part of the Foot, PL: Plasma, STO: Stomach. A: migration on a 12% gel. B: migration on a gradient 4-20% gel.

Western blot was used to assess the amount of biomphalysin B1 and B2 (hereinafter referred to as B1/2, please see Materials and Methods section) in different tissues of *B. glabrata*: anterior part of the Foot (aF), Albumen Gland (AG), Heart (H), Hemocytes (He), Hepatopancreas (HP), Kidney (K), Mantle edge (M), Ovotestis (OVO), posterior part of the Foot (pF), Plasma (PL) and Stomach (STO) (Fig 1). B1/2 is detected in all tested tissues, at a very low level in the heart and with the highest levels in the stomach, the hepatopancreas and the plasma. H3 histone was used as a loading control, the different apparent molecular weights of H3 in different tissues are due to histone variants, whereas plasma, as a cell-free compartment, doesn’t display histone protein.

Several molecular forms of B1/2 exhibiting different apparent molecular weights are detected in the soluble lysis fractions, and, surprisingly, all of them display an apparent molecular weight smaller than 52 kDa, much lower than the theoretical size of B1 at 62.8 kDa (64.7 kDa, including the signal peptide, which is cleaved in the case of such a secreted protein [18]) (Fig 1A). The largest form is exclusively detected in the albumen gland (AG) at a size slightly greater than 52 kDa. A second form, slightly smaller (annotated “1” in mantle edge tissue in Fig 1A), is present in the hemocytes (He) and in the plasma (PL), in which it seems to be the only form present, and this form is also detected in the posterior (pF) foot and in the mantle edge (M). A third smaller form (annotated “2” in mantle edge tissue in Fig 1A) is present in all tissues, except for the hemocytes (He), the plasma (PL) and the albumen gland (AG), this form is especially abundant in the stomach (STO), hepatopancreas (HP) and the ovotestis (OVO). A last lighter form (annotated “3” in mantle edge tissue in Fig 1A) is detected at a size of approximately 38 kDa for several tissues, mainly including the foot (aF and pF), the ovotestis (OVO), the kidney (K) and the mantle edge (M) (and with a weaker signal, AG and H tissues). A unique band at approximately 24 kDa can be noticed for the albumen gland (AG) tissue.

B1/2 is also detectable at molecular weights higher than 225 kDa, especially in the mantle edge tissue (M), the posterior part of the foot (pF), the hemocytes (He) and slightly detectable in ovotestis (OVO). To more precisely describe these molecular species, a 4–20% gradient gel, more resolutive to high molecular weights, was performed (Fig 1B). Two clear bands are visible for these tissues, corresponding to denaturation-resistant forms of B1/2. These bands could correspond either, to one or more B1/2 proteins bound, covalently or not, to some other unknown proteins, or, to a homo or hetero-oligomeric biomphalysin assembly. As B1/2 is an aerolysin-like protein, such assembly could resemble prepore or pore structures, as these were shown to resist denaturation and migrate as high molecular weight structures on SDS-PAGE [21–28]. A western blot performed on plasma samples and using the anti-B1/2 antibody preincubated with its specific antigenic peptide, as a specificity control, is available in S2 Fig.

### Biomphalysin 1 is strongly revealed in posterior foot and mantle edge through immunolabeling on histological slides of whole *B. glabrata* snail

**Fig 2:**
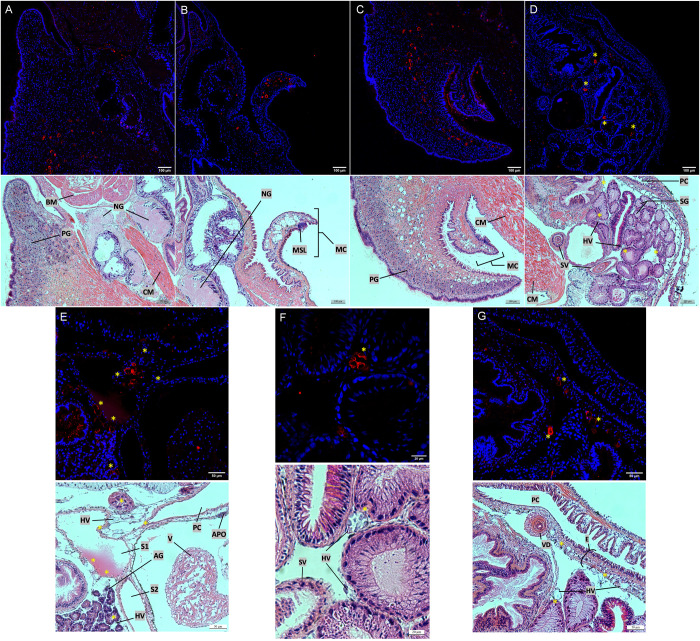
Biomphalysin 1/2 distribution in *Biomphalaria glabrata* tissues through immunohistology. Cuts are shown by pairs (A to F), an immunolabeled cut with anti-B1/2 antibody revealed through an Alexa 594-labeled secondary antibody (above), and a HES-stained cut permitting the identification of the immunolabeled tissues (below). Asterisks indicate a similar position between the fluorescence labeled cut and the HES stained one. The cuts were 3µm thick. A: Anterior part of the foot, under the buccal mass. B: Superior part of the buccal mass, centered on the mantle edge. C: Posterior part of the foot. D: First part of the visceral mass, rich in serous gland, salivary glands. E: Visceral mass, focused on heart sinuses area. F: Magnification on serous gland of the first part of the visceral mass. G: Magnification on D, focused on the paleal cavity. Abbreviation: AG: Albumen Gland, APO: Amoebocyte Producing Organ, BM: Buccal Mass, CM: Columellar Muscle, E: Epithelium (floor of the paleal cavity), HV: Hemolymphatic Vessel, MC: Mantle Collar, MSL: Median Sensory Lobe, NG: Neural Ganglia, OSL: Outer Secretory Lobe, PC: Paleal Cavity, PG: Pedal Gland, S-S1-S2: Sinus, SG: Serous Gland, SV: Seminal Vesicle, V: Ventricle, VD: Vas Deferens.

Immunoblots can provide relative abundances of B1/2 in each tissue and inform on the existence of cleaved molecular species. However, it does not allow the protein to be precisely located within organs or tissues. Such information was obtained with histological studies and immunostaining. Serial cuts of fixed *B. glabrata* were performed, and two adjacent cuts (3 µm thick), one treated with fluorescent immunological labeling and the other by Hematoxylin-Eosin-Saffron (HES)-staining, were compared to allow for precise location of B1/2 in the tissue (Fig 2).

HES staining is very contrasted in *Biomphalaria* slides and permits a good identification of the different tissues: nuclei appear in blue, muscular fibers in red and conjunctive tissue mostly in yellow (Fig 2A–2G). The pedal sole is easily recognizable through its ciliated epithelium and, deeper down, by a suprapedal gland (Fig 2A and 2C), consisting of several layers of blue-grey serous cells, responsible for mucus secretion. This suprapedal gland can also be depicted as two glands: an anterior and a posterior pedal gland [29] (Fig 2A and 2C). A mix of connective tissue and muscle fibers containing lacunae is observed in the deeper layers, especially for the posterior part of the foot. These lacunae probably constitute hemolymphatic tubules, which often contain structures, potentially cells such as hemocytes and rhogocytes (the latter appearing with an homogenous red material [30]).

B1/2 is revealed in some lacunae, especially those containing putative cellular or secretion structures (clear-blue coloration in HES). No labeling was noticed inside of the pedal gland (Fig 2A and 2C). Lacunae can be found in other organs and tissues, but they are particularly abundant in the snail mantle edge (Fig 2B), in the posterior part of the foot (Fig 2C) and in the floor of the paleal cavity (Fig 2D and 2G), providing arguments in favor of a fluorescent labeling inside of hemolymphatic capillaries. Responsible for the shell production, the mantel edge contains a median sensory lobe and an outer secretory lobe (Fig 2B) [31]. Even though this tissue contains glandular cells, it also contains several lacunae that result in strong immunolabeling of internal structures (Fig 2B).

Similar immunofluorescent pattern can be noticed close to heart sinuses (Fig 2E), and some fluorescent elements in the heart (S1 Fig), no fluorescence was detected in the stomach, albumen gland, hepatopancreas or ovotestis. Similarly, no fluorescence was observed in the hemocyte-producing organ, and only a few, if any, hemocytes were observed in the heart in contrast to previous reports (Figs 2E and S1) [32]. The same observation can be made regarding the kidney, in which no hemocyte immunostaining was noticed [33]. The same lacunar immunolabeled structures were sparsely observed between organs or tissues such as between salivary glands (Fig 2D and 2F). This labeling dispersion in the animal, very localized and strong in intensity, the nature of the labeled tissues as well as comparative histology support the idea of hemolymph vessel labeling [31,32,34,35], either through a direct labeling of the endothelial tissue or a labeling of sessile hemocytes within those vessels. Negative controls of the immunolabeling are available in S3A–S3D Fig.

**Fig 3:**
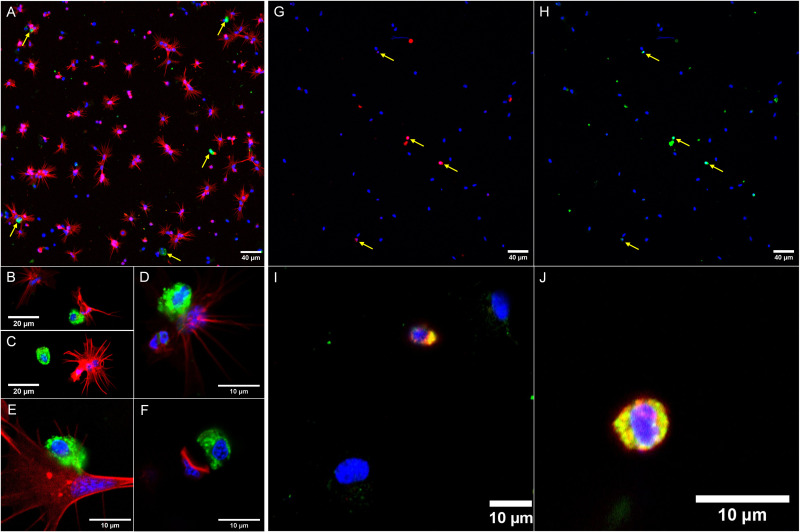
Biomphalysin 1/2 distribution in *Biomphalaria glabrata* hemocytes through immunocytology. A to F: Immunolabeling of biomphalysin 1 in hemocytes of *Bg*BS-90 strain was performed on circulating hemocytes from the hemolymph compartment. The labeling is specific to a subpopulation of blast-like cells, unbound by phalloidin. Green: Alexa488 coupled secondary antibody (B1/2), Red: Alexa594 coupled phalloidin, Blue: 4′,6-diamidino-2-phenylindole. The same immunolabeling procedure was performed on *Bg*BRE2 strain (shown in S4 Fig). A: General view on several hemocytes, showing an overview of the proportion of the B1/2-positive population. B, C, D, E: Shape and size comparison between B1/2-positive cells and other cell types such as hyalinocytes. F: B1/2-positive cells aren’t stained with phalloidin and some cells, close in size, can be negative for B1/2. G to J: Immunolabeling of biomphalysin 1 and TEP1 in hemocytes of *Bg*BS-90 strain. Immunolabeling of hemocytes by TEP1 and biomphalysin 1 antibodies shows a co-location in the same blast-like cells. Green: Alexa488 coupled secondary antibody (TEP1), Red: Alexa594 coupled secondary antibody (B1/2), Blue: 4′,6-diamidino-2-phenylindole. G: B1 labelling (same observation field than H). H: TEP1 labelling (same observation field than G). I and J: Magnification of B1/2/TEP1 positive cells.

Circulating hemocytes are constituted by different subpopulations involving at least hyalinocytes, granulocytes and blast-like cells. Generally, hyalinocytes are very adherent agranular spreading cells, granulocytes have a high density of granules while blast-like cells are characterized by a high nucleo-cytoplasmic ratio and a poor adherence capacity. According to the work of Cavalcanti and collaborators, hyalinocytes form 47% of the total hemocytes, blast-like cells form 45% of the total population, and granulocytes form 4% of the total hemocytes [36]. Those estimations vary between authors, as blast-like cells could represent 21% of circulating hemocytes, granulocytes about 12% and hyalinocytes 66% [37]. By immunocytology approach (Fig 3), only a minor subgroup of blast-like hemocytes was stained with the anti-B1/2 antibody (Fig 3A). This suggests that not all the hemocytes are able to produce B1/2 but only some small round cells among the circulating hemocytes (Fig 3B–3E). Similar results were obtained on *Bg*BRE2 strain (see S4 Fig). Those cells can’t be revealed using tagged phalloidin (Fig 3F) and are costained following the use of anti-TEP 1 antibody (Fig 3G–3J), which allows the identification of a subpopulation of hemocytes able to produce at least two distinct abundant immune-related plasmatic proteins. This subpopulation of blast-like cells could play a role in the secretion of constitutive immune components. Negative controls of the immunolabeling are available in S3E and S3F Fig.

### Optical tissue clearing of *B. glabrata* reveals possible mucus glands through B1/2-immunolabeling

**Fig 4:**
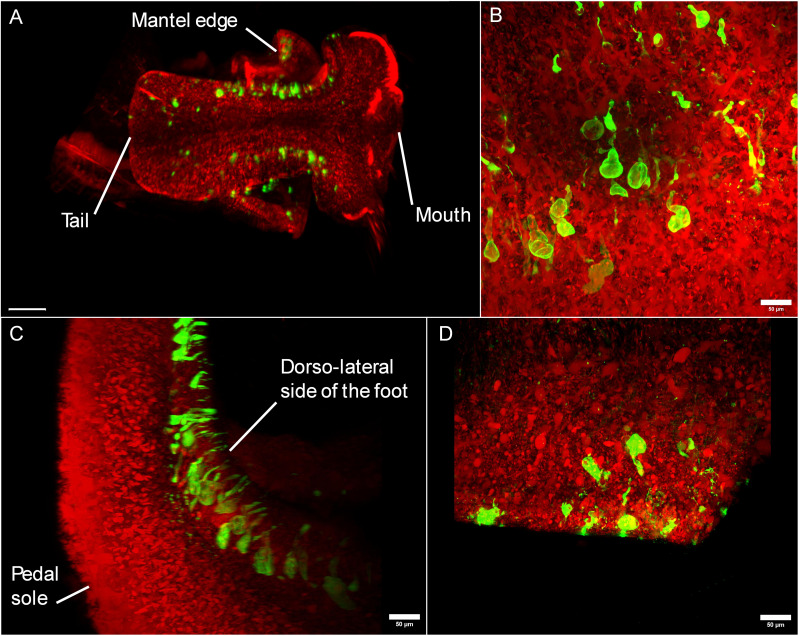
Lightsheet and confocal visualizations of biomphalysin 1/2 immunolabeling on tissue-cleared *Biomphalaria glabrata* snails. Tissue clearing was performed using X-CLARITY system, after fixation snails are incubated in acryl solution and optically cleared through electrophoretic delipidation using SDS. Propidium iodide (red) allows to locate snail tissues by DNA staining around B1/2 labeling (green). A: Z-stack of the head-foot region of a tissue-cleared snail (ventral view using a lightsheet microscope) scale bar = 300µm, B: Z-stack facing the pedal sole of the foot, C: 90-degree horizontal rotation of stack B, highlighting the dorsal outlet of the pear-shaped structures labeled in the foot, D: Mantel edge of the snail, exhibiting similar biomphalysin 1/2-labeled structures. B, C and D pictures were taken using a confocal microscope. Red: propidium iodide, green: immunolabeling of B1/2. A short video showing the rotation between picture B and C is provided in S5 Fig.

To improve the identification accuracy of the labeled structures observed in immunohistology experiments, tissue clearing coupled to B1/2-immunolabeling was performed on snails (Fig 4). Strongly labeled structures were observed in the dorsal part of the foot (Fig 4A–4C), and the mantle edge (Fig 4D) of the considered specimens, in accordance with the labeling observed for the immunohistology assay. Those structures seem to exhibit an analogous shape and location to dorso-lateral mucus glands [38–40]. The structures are pear-shaped and bear a duct, reaching the outer medium. A video showing the rotation between B and C pictures (Fig 4) is provided in S5 Fig.

### Biomphalysin transcriptional response following microorganism immune challenges

**Fig 5:**
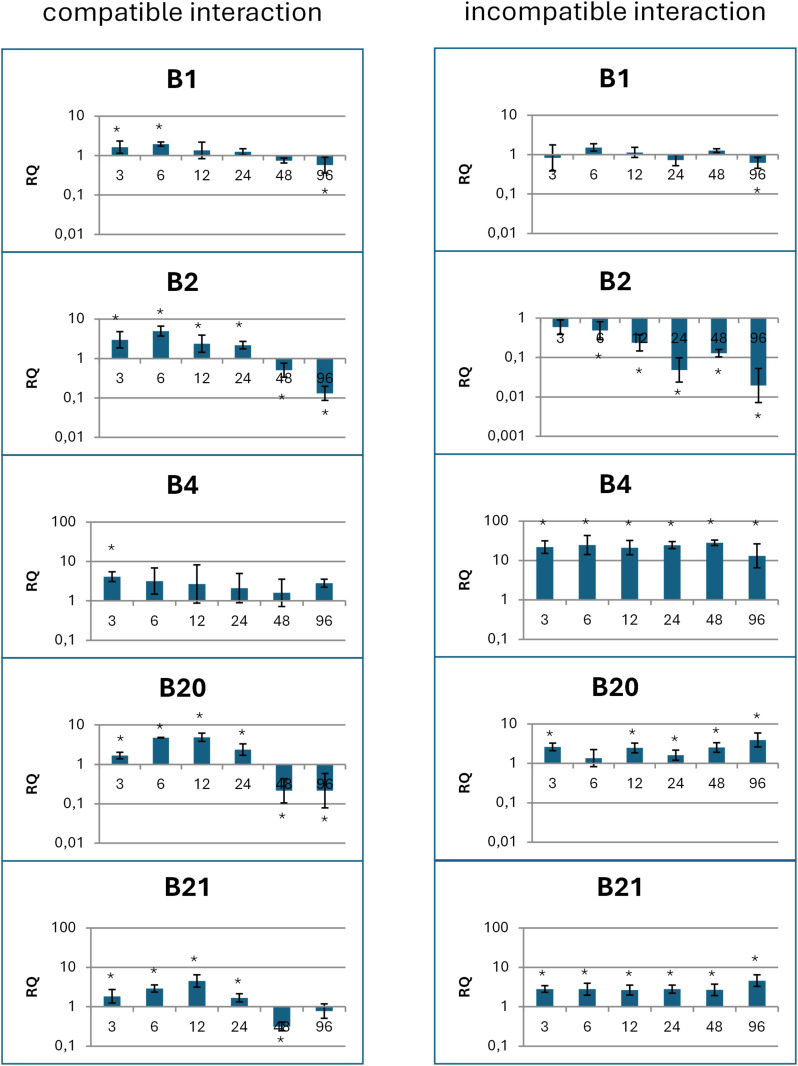
Biomphalysin genes expression in response to *S. mansoni* intrusion. qRTPCR was performed on whole snail organisms exposed to *S. mansoni* either in the case of a compatible (*Bg*BRE2 snail and *Sm*BRE parasite strains) or an incompatible interaction (*Bg*BRE2 snail and *Sm*GH2 parasite strains). Expression was measured at six time points (3, 6, 12, 24, 48 and 96 hours) and was normalized to S19 housekeeping gene expression and compared with the expression obtained in non-exposed snails. Asterisks indicate a significant difference between non-exposed and exposed snails (p < 0.05). RQ: Relative Quantification (2^-ΔΔCT^).

The expression level of the five most transcribed biomphalysins (*B1*, *B2*, *B4*, *B20* and *B21*) [18] after infection with *S. mansoni* miracidia were measured by qRTPCR following two conditions of interaction (compatible: infection of a Brazilian *Bg*BRE2 snail strain by a Brazilian *Sm*BRE parasite strain and incompatible: infection of a Brazilian *Bg*BRE2 snail strain by a Guadeloupean *Sm*GH2 parasite strain, Fig 5). After infection, in a compatible interaction (*Bg*BRE2/ *Sm*BRE), expression levels of *B2*, *B20* and *B21* genes first increase for 24h and then decrease. In an incompatible interaction (*Bg*BRE2/ *Sm*GH2), the upregulation of *B20* and *B21* genes is maintained along the experiment (Fig 5), while it is mostly repressed in the case of *B2*. *B4* is significantly upregulated in a compatible interaction (4-fold) and even more so in an incompatible interaction (27-fold). *B1* gene expression seems to be very stable across the kinetic of infection, in either compatible or incompatible interactions (Fig 5).

To investigate the effects of immune challenge on the expression of each biomphalysin, snails were also exposed to different microorganisms: *E. coli*, *M. luteus*, *S. cerevisiae* or *S. mansoni* (S6 Fig)*.* As a result, most of biomphalysin coding genes are upregulated in the beginning of the exposure and then, downregulated. This pattern could exhibit a generic response to a stress without any specificity to a particular type of microorganism.

### Biomphalysin 1/2 forms oligomers after binding to several microorganisms or cells

**Fig 6:**
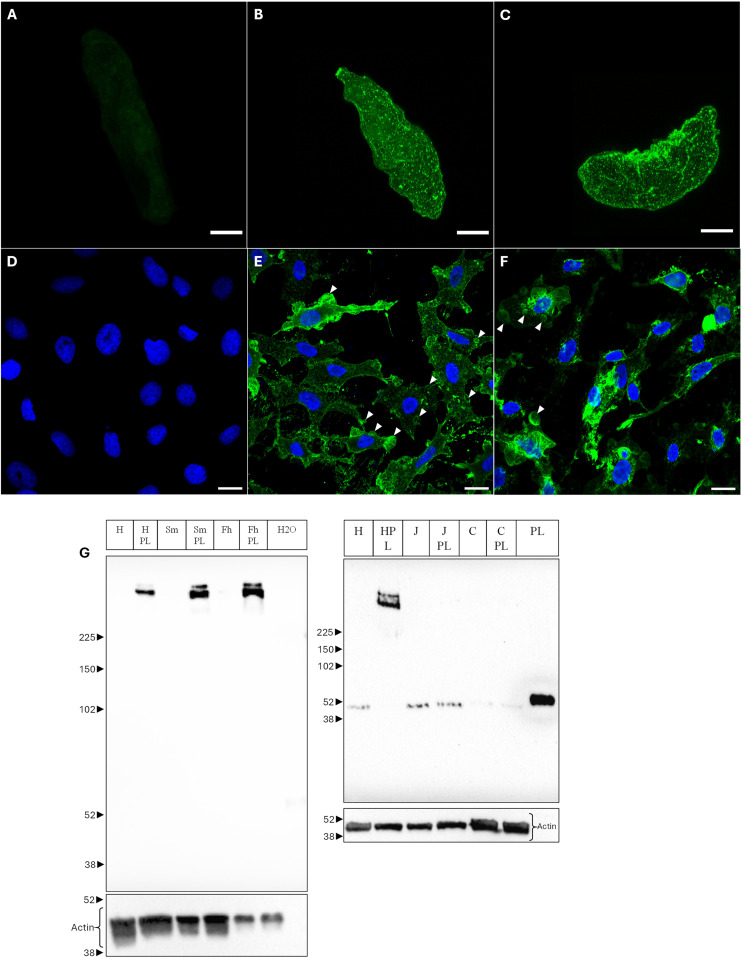
Biomphalysin 1/2 binding to several cell types. A, B, C, D, E, F and G: Confocal observation of biomphalysin 1 binding to *S. mansoni* sporocyst **(A to C)** and HeLa cells **(D to F)**. Z-stacks are shown here for sporocysts and one focal plane for HeLa cells. Unexposed sporocysts were used as control, maintained in CBSS **(A)**. Sporocysts were incubated with plasma for one hour, washed with CBSS and immunolabeled using anti-B1/2 antibody **(B and C)**. Scale bars are 20µm. Labeling is only present at the surface of the sporocysts. HeLa cells were exposed either to CBSS **(D)**, or undiluted ultracentrifuged plasma **(E and F)** for 1 hour. HeLa cells are not lysed following CBSS exposure. Green: Alexa488-coupled secondary antibody (B1/2), Blue: 4′,6-diamidino-2-phenylindole. Sale bars are 20 µm. White arrowheads show some of the multiple vesicles from different sizes visible on and around the lysed HeLa cells. Those vesicles range in diameter from about 1 µm for the smallest to over 10 µm for the largest. Cells or microorganisms were incubated with ultracentrifuged plasma for two hours (plasma final dilution of 1/2), washed and mixed in reducing Laemmli buffer for gel migration **(G)**. For each cell type, one well is dedicated to the plasma-exposed condition (e.g., *HPL*) and one well to an unexposed condition (e.g., *H*). C: C2C12 cells, Fh: *Fasciola hepatica* miracidia, H: HeLa cells, H2O: Water in which *F. hepatica* eggs hatched (control), J: BCL2 Jurkat cells*,* Pl: Plasma, Sm: *Schistosoma mansoni* mother sporocysts (48h transformation).

Binding of B1/2 on sporocysts surface exhibits a particular non-homogenous pattern. This binding pattern is organized into spots on sporocyst membrane. Being syncytial, sporocyst membrane may be likened to the plasma membrane of a single cell [41,42] regarding its external side (Fig 6A–6C), we thus can define the binding pattern of B1/2 on sporocyst surface as spots of enriched biomphalysin binding. Ultracentrifuged *B. glabrata* plasma has a lytic effect against HeLa cells and B1/2 can bind to their membrane (Fig 6D–6F). The binding is particularly abundant, and multiple vesicles can be seen on and around the cells following cell lysis (arrowheads, Fig 6E and 6F). Those vesicles are also shown in S7 Fig, in an optical observation of the cells in white light.

After exposure to snail ultracentrifuged plasma, microorganisms or cells were lysed, their protein content separated by reducing SDS-PAGE, and B1/2 revealed by western blotting. Pairs of bands at high molecular weight forms are detected only when binding is observed (Fig 6G). Such binding is observed for HeLa cells, *S. mansoni* sporocysts and *F. hepatica* miracidia but not for BCL2 Jurkat cells or C2C12 cells. Regarding the three cancer cell lines studied here, we moreover noticed a correlation between the ability of biomphalysin 1/2 to bind and the plasma lytic property towards the exposed cells (HeLa cells were all lysed within a few minutes while C2C12 and BCL2 Jurkat cells did not exhibit such a lytic aspect, shown in S7 Fig).

### Abundance of plasmatic biomphalysins following infection by *Schistosoma mansoni*

The strongest differences observed in biomphalysin gene expressions occur in the context of compatible and incompatible interactions of *B. glabrata* with *S. mansoni*. Hence, we aimed to identify the biomphalysins that are present in the plasma in each case.

Plasma samples were migrated on SDS-PAGE, colored by Coomassie blue and bands corresponding to the biomphalysin monomer size, as seen on Western blot experiments (Fig 1) were cut (S8 Fig). The identity and abundance of each plasmatic biomphalysin was then investigated by mass spectrometry (MS)-based quantitative proteomics. Specific peptides for B1 (B2), B4, B5, B8 and B20 were identified. No significant differences between the compatible and the incompatible conditions could be detected regarding protein identity and abundance.

**Fig 7:**
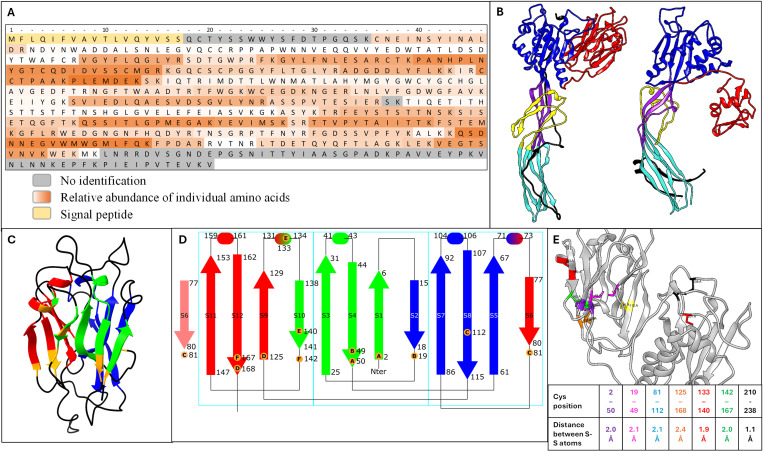
New insights related to predicted structure of biomphalysin 1, based on alphafold2 aminoacyl pair representation and mass spectrometry data support. A: Abundance of biomphalysin 1 individual amino acids, permitted by peptide coverage and determined through mass analysis of plasma compartment. Biomphalysin 1 reference sequence provided by NCBI, NP_001298219. Identified peptides for biomphalysin 1 and 2 following mass spectrometry analysis were aligned on the same sequence of biomphalysin 1. Identified amino acids are colored from white to dark orange depending on their relative representation among identified peptides. Unidentified amino acids are colored in gray (except for the signal peptide colored in yellow). B: Biomphalysin monomer structure prediction (left) and comparison to proaerolysin crystal structure (right). PDB accession number: 3C0N. By analogy with aerolysin structure: domain I (red), domain II (blue), domain III (purple), domain IV (cyan), transmembrane domain containing the insertion loop (yellow) and unfolded Cter residues (black) were defined. Small lobe = domain I (red), large lobe = domains II, III and IV. C: Ribbon representation of biomphalysin 1 domain I (including the Nter residues). Each face of the β-prism is colored in a different color. Cysteines are highlighted in orange. There is an accordance between the non-organized Cter sequence shape in structure prediction and the lack of detection of the Cter sequence shown in A. D: Topological representation of biomphalysin 1 domain I. β-strands are depicted with arrows and α-helices with cylinders. The boundaries of each secondary structure element are indicated, as well as the positions of cysteine residues, depicted with orange circles. The six disulfide bridges are indicated with capitalized identical bold letters. The repeated structural patterns are framed in cyan. E: Position and distance between close cysteines within domain I (small lobe) and domain II.

Several peptides specific to B1 and/or B2 were obtained, which were mapped onto the full sequence of B1 and B2: 87.07% of sequence coverage was obtained, excluding the peptide signal sequence (Figs 7A and S9). Undetected peptides of B1 and B2 are mostly found at the C-terminal part of the protein (Fig 7A). This C-terminal portion could theoretically be detectable by MS-based proteomics following trypsinization (S10 Fig), as the theoretical size of trypsinized peptides is in the range of MS-detectable peptides. Interestingly, the protein devoid of this C-terminal part has a theoretical molecular size of 54,5 kDa which is relevant regarding the size of the protein observed after western blotting (Fig 1). This plasmatic truncated form could be the main active form for B1/2 due to its tremendous abundance in the whole organism and its, *de facto*, ubiquitous distribution through the open circulation hemolymphatic system and the mucus.

### Structural insights on biomphalysins: Small lobe secondary structure predictions, C-terminal peptide and overall architecture prediction

Biomphalysins are challenging to produce as soluble recombinant protein in *Escherichia coli*. Consequently, no experimental structural data is available and AlphaFold2 was used to predict the structure of the 23 proteins with or without the last 57 C-terminal residues (“full size” or “ΔCter”, respectively). For each prediction, this peptide is predicted as unfolded (Figs 7B and S11A).

In the case of B1, these residues correspond to the non-detected C-terminal peptide (residues 493-550) in MS-based proteomic analyses of plasma samples (Figs 7A and S11A). The pLDDT scores obtained were similar for both predictions of B1 (B1 full size: 92.9 and B1 ΔCter: 91.4). The predicted structure of full-size B1 displays two structural domains: the N-terminal domain (residues 1-173), that adopts a β-prism fold, and a large C-terminal aerolysin-like domain (residues 178-499) (Fig 7B). As residues 500-555 are predicted to be mostly non-structured, the structural description will focus on residues 1-499 corresponding to B1 ΔCter. The 55 aminoacyl long C-terminal peptide is structurally equivalent to the C-terminal peptide of aerolysin, which must be cleaved to allow for oligomerization and pore formation (Fig 7B) [43]. The N-terminal domain (residues 1-173) shapes into three 4-strand antiparallel β-sheets that form a β-prism, with a pseudo-3-fold symmetry stabilised by 6 disulphide bridges (Figs 7C, 7D and S12). The prism is constructed of 3 consecutives 4 β-strands motives, with the first, third and fourth strands forming a part of one side of the prism, and the second strand being part of the neighbouring side. Disulfide bonds are predicted to link the second to the third strand in all three motives (labeled B, C, and F in Fig 7D), and another disulfide bond attaches the first strand to the third one on two motives (labeled A and D in Fig 7D).

The aerolysin domain of B1 adopts an identical topology as aerolysin (S13 and S14 Figs). Domains II and III, as defined in the aerolysin (Fig 7B), are highly similar and superimpose onto each other with an RMSD (Root Mean Square Deviation of atomic positions) of 3.9 Å (165 Cα atoms). Domains IV of each protein display largely different orientations, resulting in a rather linear overall form for the aerolysin domain of B1 compared to the curved conformation seen in the structure of the soluble form of aerolysin. The structure of B1 seems closer to the aerolysin protomer in the prepore conformation (Figs 7B and S13). In B1, the aerolysin fold is built around two long twisted central antiparallel β-strands (strands s15 and s20) that encompass the complete length of the protein and serve as a scaffold to build the 3 domains. In domain II, three additional strands (s13, s14 and s15) allow the formation of a 5-strands β-sheet upon which 4 α-helices are packed: the long helix h6 that links the N-terminal domain to the aerolysin fold and helices h7, h8 and h9. In domain III, a 5-strand antiparallel β-sheet is also found, built from the two long central β-sheets and strands s15, s19 and s22, onto which an additional 2-strand β-sheet (s17 and s18) packs diagonally. In domain IV, the 5-strands β-sheet of domain III collapses into a β-sandwich: strands s16 and s19 are twisted and pack onto the 3-strands β-sheet made of the continuation of strands s15, s20 and s22 (details of the topological representation can be seen in S14 Fig). As the structure of B1 resembles that of aerolysin in the prepore state, AlphaFold2 multimer was used to predict a possible heptameric state of B1 full length and B1 ΔCter, that would correspond to a prepore state of biomphalysin 1 (S11 and S13 Figs). In that case, a better pLDDT score was obtained in the case of B1-ΔCter compared to B1-full length (83 and 77.3, respectively). The analysis of interfaces in the resulting heptameric assemblies with PISA also suggests that B1-ΔCter is more likely to form such assembly than B1 full length, as the average buried surface area are 1417 and 581 Å^2^, respectively, to be compared to the 1835 Å^2^ in the prepore structure of aerolysin (5JZH).

When taking a closer look at the B1 predicted model, there is a total of 15 cysteines: 12 grouped in domain I and 3 in domain II. Nine are fully conserved between all biomphalysins, and two more if we exclude biomphalysin 23 (S12 Fig). They form seven pairs with an inter-sulphur distance between 1.1 Å and 2.4 Å (Figs 7E and S11), which suggests they can easily form disulfide bonds. It can explain why recombinant production is difficult, as *E. coli* cytoplasm is a reducing environment in which disulfide bridges cannot form. These potential covalent bonds imply a very stable domain I structure/preserved 3D structure despite low sequence similarity (S11B and S15 Figs). Aerolysin is a pore-forming toxin, which, upon membrane binding, oligomerizes and forms a β-barrel pore. As the structure of B1 is highly similar to the structure of the aerolysin protomer in the prepore conformation, we can hypothesize that strands s16, s17, s18 and s19 of B1 would correspond to the β-barrel forming part of aerolysin (Figs 7D and S13).

## Discussion

As the sole proteinaceous lytic effector in the plasma, biomphalysins, and more specifically, the most abundant B1/2, bear a strong role in the humoral immunity of *B. glabrata*. Our histological and cytological work reveals the production of B1/2 by a specific subpopulation of blast-like cells. These cells can also produce TEP1 [44], which allows us to define a subpopulation of hemocytes unlabeled by phalloidin and dedicated to the production of two critical humoral immune factors. B1/2 is also revealed in potential hemolymphatic vessels across different tissue locations. Some tissue-specific capillaries may host some sessile hemocytes that cannot be identified during the hemolymph extraction, suggesting that immunolabeled tissues observed in Figs 2 and 3 could be the producing sites of B1/2 in the snail. However, the strongest labeling in dorsolateral foot and mantle mucus glands extends the immune function implication of B1/2 to complete the defensive role of the external mucosal barrier. The finding of B1/2 secreted on the external surface of the snail is consistent with Fogarty’s work on the potential involvement of biomphalysins in a chemoattractant role towards miracidia [45], meaning biomphalysins can be secreted on the snail’s epidermis and in extension, in the environment.

There is a correlation between the tissues labeled on histological slides and organs known for their biomphalysin 1 transcriptional activity [18]. Moreover, a specific size pattern of B1/2 protein, revealed by western blotting (Fig 1), can be observed for those tissues: there is a correlation between the tissue distribution of the protein as seen with microscopic approaches and the tissues containing the intermediate monomeric size in western blotting, annotated as band 2 in Fig 1 (mantle edge, posterior part of the foot, circulating hemocytes, plasma, kidney -including paleal cavity floor after dissection-). The detection of the light 38 kDa band is puzzling (annotated as band 3 in Fig 1). Indeed a protein size of 38 kDa doesn’t allow for an overlapping with full predicted functional protein domains of biomphalysin 1 [18]. Moreover, considering that the primary antibody used for these blots is designed on a peptide located in the N-terminal part of the small lobe, if this 38 kDa band constituted a shortened biomphalysin 1 protein form, it would contain the domain I and a truncated part of the aerolysin lobe, which may conduct to a non-functional form of the protein [17,18]. Due to the lack of intronic sequences among the *biomphalysin* genes, a reduction in the size of the true protein can mainly be induced by one or several proteolytic cleavages. The same reasoning can be applied to the 24 kDa band noticed in albumen gland (Fig 1), as this protein size covers the full domain I and a part of the domain II.

From the molecular point of view, it is hard to explain the high molecular weight forms found in tissues (hemocytes, rear of the foot and mantel edge) for B1/2, especially migrated on SDS-PAGE in reducing conditions (Fig 1). Two hypotheses include: the covalent binding between B1/2 and an unknown protein, making it detectable at an unexpected size, or the presence of a tissue-specific oligomeric form resistant to denaturation [21–25]. It has been shown that β-PFT could form some highly resistant oligomers, such as pore- or prepore-like structures. Hence, B1/2 could aggregate in such a resistant oligomeric structure. However, this observed size could also be an artifact due to contact with post-lysis cellular material. In some tissues, the monomeric form of B1/2 exhibits a slightly different size than the plasmatic one, as observed with the western blot experiment (especially for the albumen gland with a larger size and stomach and hepatopancreas as examples with a smaller size). These results are not correlated with a specific immunohistology pattern, perhaps because biomphalysin 1 is not abundant enough to be detected on a slide, as could be the case for albumin gland (except for the presence of hemolymph vessels, Fig 2D–2F). Stomach and hepatopancreatic tissues could contain ectopic plasma due to the open-circulatory system, either for stomach due to the shell removing of the snail (still living at this step and consuming its own hemolymph) or for the convolutions of hepatopancreas, trapping infiltrated plasma. On cuts, those tissues don’t exhibit a positive labeling for B1/2 (S1 Fig)

Apart from B4, for which an incompatible interaction with *S. mansoni* miracidia induces a stronger transcription, *biomphalysins* only display slightly altered transcription levels upon contact with microorganisms (Figs 5 and S6). However, as revealed by MS-based quantitative proteomics, this does not translate into qualitative or quantitative modifications of corresponding proteins in plasma.

The lack of plasma protein changes can be explained by the fact that cellular immunity is mostly implied in the elimination of parasites and other microorganisms. Biomphalysin 2 has been identified in previous protein analyses of hemocyte capsules formed around larval *S. mansoni* (protein id: XP_013070389.1, [46]). However, no differential detection of biomphalysin 2 protein has been revealed between the “hemocytes alone” and “encapsulated” conditions, underlining the absence of biomphalysin concentration within the capsule [46]. Biomphalysins (other than B1 and B2) could still have functions other than immunity and may not be required in abundance within the plasmatic compartment. If biomphalysins are implied in microorganism killing, the need for B1/2 to be assisted by plasmatic cofactors [17] could be linked to an enhanced expression or activation of molecular partners of biomphalysins rather than to a regulation of their own expression during an immunogenic contact.

Only select biomphalysins were detected in the plasma. Biomphalysin 1 and 2 were the most abundant, with only a few specific peptides being detected for the others (B4, B5, B8, and B20). Some peptides of biomphalysin 1 and biomphalysin 2 matched sequences from the NCBI non-redundant database while some others matched theoretical sequences from the Vectorbase database. This indicates that there may be slight variations in biomphalysin sequences from one snail individual to another (S9 and S10 Figs). Furthermore, when looking at the most recent *B. glabrata* genome assembly, which allows for chromosomal discrimination of the genes, performed by the Wellcome Sanger Institute (xgBioGlab47.1, NCBI RefSeq assembly: GCF_947242115.1), new variations of biomphalysin sequences can be found compared to previous biomphalysin sequences (S16 Fig). Also, the gene positions of *biomphalysins* are not completely consistent with the phylogenetic proximities previously established [18]. Some genes are closely related in nucleotide sequence similarity [18] and are in close proximity (as B1, B2 and B3 on chromosome 6 or B4, B6 and B7 on chromosome 14). However, other *biomphalysins* are located in close proximity on chromosome 8, but protein sequences are not necessarily defined as closely related [18] (S16 Fig).

Mapping the identified peptides on B1 sequence showed that a C-terminal portion of the protein may be missing, which is in line with western blotting results and the structural prediction of the protein (absence of secondary structure for this C-terminal portion). The identified plasmatic truncated form of biomphalysin 1 can be linked with the activation pattern of aerolysin (which is the representative for this toxin family). Indeed, aerolysin from *Aeromonas hydrophila* requires proteolytic cleavage of its C-terminal peptide sequence [47], as is the case for some other β-PFT [24,48]. It seems that this cleavage is performed before biomphalysin secretion; the monomeric full form of biomphalysin 1 cannot be detected by SDS-PAGE analysis for the tissular hemocyte or plasmatic compartment. It has been demonstrated that this C-terminal peptide functions to avoid aerolysin aggregation during its synthesis [43], which *de facto* does not seem to be the case for biomphalysin 1.

The abundance and distribution of biomphalysin 1 in *B. glabrata* (abundant in the humoral fraction of the open circulatory system) could be linked to the role of a sentinel protein, ready to act in front of intruder microorganisms. It was demonstrated by an interactomic approach that biomphalysins were able to bind to several microorganisms [20]. However, it was not possible to discriminate which one of the biomphalysin proteins bound to which cell type. Here we demonstrated that native plasmatic biomphalysin 1 can bind to *S. mansoni* sporocysts, *F. hepatica* miracidia, and HeLa cells, only under a denaturation-resistant oligomeric form (Fig 6). There are two ways to understand this range of binding. Firstly, B1/2 binds directly to several cell types, which suggests that either this protein has a certain range of molecular targets, or those cells share a common ligand for B1/2. The other possibility is that other plasmatic biomphalysins can bind those cell types first, and B1/2 acts as a support for oligomerization, potentially forming hetero-oligomers of biomphalysins. The two high molecular weight bands observed (Fig 6) could be considered either as homo- and hetero-oligomers, or as oligomers with a different valency, or as oligomers at a different conformational step (such as pre-pore and fully functional pore). The predicted model of biomphalysin 1 shows several structural features found in the aerolysin family: three long β-strands (s15, s20 and s22) forming the common aerolysin core, a variable loop (s21, h7, h8 and h9) between the last two strands and an insertion loop (s17 and s18). In known pore-forming toxins such as aerolysin or ε-toxin, this portion unfolds to form the transmembrane pore [7,49]. These observations support the idea that biomphalysins, like aerolysin, can oligomerize and their lytic activity may be similar to pore formation after binding to a specific target.

The analysis of the predicted structure also suggests that the structure is conserved due to the preservation of disulfide bonds across the 23 biomphalysins. The small lobe has a typical β-prism shape. This shape resembles the structure of the typical lectin family of jacalin and jacalin-like lectins (Fig 7) [50]. This work updates the structure predictions previously established [18].

Belonging to the family of aerolysin and regarding to the binding pattern of biomphalysin 1/2 observed on sporocysts (Fig 6), assumption can emerge relating to a binding to lipid rafts or membrane microdomains [25,51–53]. The observed binding at sporocysts’ surface is organized into spotted clusters. Because the sporocyst membrane is syncytial, it behaves as one cell. The binding on HeLa cells overall follows a spotted pattern, with a high concentration of B1/2 on vesicles after cell lysis. This cell lysis may arise directly from biomphalysin binding or from other immune factors from the plasma. As there is a correlation between cell lysis, B1/2 binding and oligomerization, this suggests complete plasma activity against HeLa cells, contrary to the BCL-2 and C2C12 cell lines. Therefore, and considering biomphalysins as the only plasma effectors exhibiting a lytic ability [17], we can emit the hypothesis of a causative link between B1/2 binding and HeLa cell lysis. Additionally, that biomphalysin 1/2 can bind to a human cancer cell line is noteworthy. It is of scientific interest but also suggests that future investigations into the functions of biomphalysin 1 may be undertaken in this more defined cell system. It is important to go further in this work to identify the range of cells B1/2 can bind (cancerous or not), the molecular targets present on those cells and its potential lytic activity.

In summary, the findings of this study provide crucial insights into the distribution and role of biomphalysin 1/2 (B1/2) in the medically important snail, *B. glabrata.* Biomphalysin 1/2 is detected in various tissues. Notably, B1/2 exists in multiple molecular forms, with significant differences in size depending on the tissue, suggesting differential post-translational modifications or interactions with other biomphalysin proteins. Histological and immunofluorescence analyses revealed that B1/2 is present within hemolymphatic capillaries, glands of the foot, implying a potential role in tegument protection, and certain subpopulations of hemocytes, particularly blast-like cells, indicating its role in the immune response and providing insight into the immunological function of the blast-like cells. Moreover, structural predictions of B1 highlight its similarity to the aerolysin family, hinting at its potential mechanism of action as a pore-forming toxin. The binding ability of B1/2 to different cell types, its abundance in the plasma compartment of naive snails and its distribution among specific tissues of the organism, added to its low regulation following infections and the fact that biomphalysins are the only plasma proteins in *B. glabrata* known to exhibit a lytic activity [17] underline the potential role of B1/2 as an effector of first line of defense in the humoral fraction of the hemolymph.

## Materials and methods

### Ethics statement regarding snail maintenance and parasite uptake

*Bg*BS-90 and *Bg*BRE2 *B. glabrata* strains were maintained under constant laboratory conditions. Snails were maintained at 26°C in glass aquaria and fed with green leaf lettuce *ad libitum*. Miracidia from *S. mansoni* were uptaken from hamsters’ livers and put in CBSS for transformation into mother sporocysts. Our laboratory holds permit #39910-2022121915564694 (APAFIS number) for experiments on animals from the French Ministry of Agriculture and Fisheries and the DDPP Languedoc-Roussillon (Direction Départementale de Protection des Populations). The housing, breeding, and animal care of the utilized animals followed the ethical requirements of our country. The researchers also possess an official certificate for animal experimentation (Decree # 87–848, October 19, 1987). Animal experimentation followed the guidelines of the French CNRS. The different protocols used in this study had been approved by the French veterinary agency from the DDPP Languedoc-Roussillon (Direction Départementale de Protection des Populations), Montpellier, France (authorization # 007083) and the Ethic committee CEEA-LR (Comité d’Éthique en Expérimentation Animale – Languedoc-Roussillon): # C66-136-01.

### Histological study by western blotting

Five snails from the *Bg*BRE2 strain snails were fasting 2 days before sampling. Then snails were dissected, tissues were briefly rinsed in CBSS (not for the hearts), and tissues were pooled (five individuals for each tissue) and stored at -80°C until use. Eleven different tissues were collected: anterior part of the Foot (aF), Albumen Gland (AG), Heart (H), Hemocytes (He), Hepatopancreas (HP), Kidney (K), Mantle edge (M), Ovotestis (OVO), posterior part of the Foot (pF), Plasma (PL) and Stomach (STO). Tissues of five snails were homogenized, lysed by sonication and protein dosage was performed to normalize deposition on gel. Tissues were lysed by sonication in a lysis buffer (50 mM Tris, 150 mM NaCl, 1% NP-40, 5 mM EDTA), 10 cycles of 30 sec each at 70% intensity at 4°C were performed using a GM mini20 Sonoplus (Bandelin electronics) and a MS1.5 probe. Centrifugation was then performed at 10000 × g at 4°C for 20 min, soluble and insoluble fractions were thus separated. Total protein concentration of the soluble fraction was measured using the 2D-Quant kit (Cytiva, GE80-6483-56) after the making of a standard curve. Before performing SDS-PAGE, soluble fractions were put in Laemmli buffer containing β-mercaptoethanol and heat at 99°C for 10 min. 8.3 µg of protein were deposited for each tissue sample on a pre-cast 12% polyacrylamide BioRad gel (4568046).

The anti-biomphalysin 1/2 antibody used in Western blot was obtained from rabbit using a biomphalysin 1 peptide (INALDRNDVNWADDA) and purified from serum on a peptide-coupled affinity column. This antibody was used to perform all the subsequent immunolabeling. The chosen immune peptide exhibits a strong enough similarity between B1 and B2 for considering the antibody to target both B1 and B2 proteins. An alignment using the Clustalω tool from BioEdit software [54] was performed to highlight the position of the peptide in B1 and B2 and its lack of homology with other biomphalysins (can be seen in S17 Fig).

The second gel was run for higher molecular weight analysis (4–20% gradient gel). Blots were performed on a PVDF membrane using the anti-biomphalysin 1/2 antibody and revealed using anti-rabbit HRP-tagged antibody (ref. 31480, Invitrogen). Anti-H3 antibody was used (ref. ab1791, Abcam) as a loading control. During western blots, primary antibody incubations were performed for 90 min, using the antibody at a 1:5000 dilution (anti-B1/2 antibody initial mass concentration: 1.2mg.ml^-1^), secondary antibody incubations were performed for 70 min (1:5000 dilution). Both incubations were performed at room temperature (21°C).

A control western blot was performed in the same experimental conditions, preincubating the primary antibody with the antigenic peptide at a molar ratio of 1:20 (antibody:peptide), the figure is available in S2 Fig.

### Histological and cytological studies by immunolocalization

*Bg*BS-90 individuals were used. Snails were fasting 4 days before sampling. Then, they were fixed in 4% paraformaldehyde at 4°C for 16 hours. After Phosphate Buffered Saline washes, samples were placed successively in two 70% ethanol baths for 5 and 30 min, in two 95% ethanol baths for 20 and 45 min, then in three consecutive 100% ethanol baths respectively for 60 min, 90 min and 120 min. After those dehydration steps, samples were incubated in three xylene baths consecutively for 45 min, 60 min and 75 min. Paraffin embedding was performed through three successive incubations of 45 min, 75 min and 120 min long. Paraffin embedding was performed with a Leica ASP300 tissue processor for paraffin infiltration and a Leica Arcadia embedding center. Microtome cuts were 3 µm width and two consecutive cuts were used, first for immunofluorescence and the second for histological staining. Briefly, the paraffin was removed from the sections using xylene, sections which were then rehydrated by several washes containing ethanol in decreasing concentrations. Mayer hematoxylin (0,1%) was then used to perform staining of negatively charged cellular sites. Sections were then incubated in increasing ethanol concentrations to enable alcoholic eosin (0,25%) and saffron (1%) staining, respectively for positively charged areas and collagen and extracellular matrix staining (HES staining). Staining steps were performed with a Leica Autostainer XL. Fluorescent labeling was performed using Invitrogen Alexa Fluor 594 anti-rabbit antibody (A-11072) to reveal anti-biomphalysin 1/2 antibody. No antigen unmasking was performed on the slides after xylene paraffin dewaxing and hydration. Observations were made through a Zeiss LSM 700 microscope for immunolabeled slides and through Nikon, TS100 inverted microscope for histologic stained slides. Negative controls were performed, either using pre-immune serum of the same rabbit used to produce anti-B1/2 antibody or incubating the antibody with the antigenic peptide at a molar ratio of 1:10 (antibody:peptide). This control is available in S3 Fig.

Hemocyte immunolabeling was performed on *Bg*BS-90 strain cells and *Bg*BRE2 strain cells. Snails of both strains were fasting 2 days before recovery. Then, hemolymph was recovered through the foot retraction method and placed on a polystyrene slide (Life Science Products, S-5689), letting the hemocytes platting for 30 min. Plasma was removed, hemocytes were washed with CBSS and fixed with 4% paraformaldehyde for 10 min before immunostaining. Cells were permeabilized and saturated using a solution of 0.1% Triton X-100 (v/v) with 1% Bovine Serum Albumin (BSA) (w/v) in PBS. Immunolabeling was performed with biomphalysin 1/2 antibody diluted in 1% BSA solution in PBS. A secondary anti-rabbit antibody diluted in BSA 1% solution in PBS was used to reveal primary antibody (either Alexa Fluor 488-coupled, or Alexa Fluor 594-coupled, respectively: A-11070 or A-11072, Invitrogen).

TEP1 immunolabeling was performed following the same features as biomphalysin 1/2 antibody but using an anti-rat secondary antibody (A-11006, Alexa Fluor 488-coupled antibody). TEP1 antibody was designed on a part of the N-terminal sequence of the protein (VRTETVS…FNATFKV (residues 204-521)) and purified from rat serum. Observations were made through a Zeiss LSM 700 microscope. A negative control was performed using the secondary antibody only, either the anti-rabbit (A-11070 for B1/2) or the anti-rat antibody (A-11006 for TEP1) in the same condition previously described. This control can be seen in S3 Fig.

### Histological study by immunolocalization on cleared specimens

*Bg*BRE2 specimens were used. Clearing procedure was performed using X-CLARITY system (Polymerization System and Clearing System II) located at network facilities RHEM-Biocampus in Montpellier. The whole procedure was carried out through three steps: sample fixation, hydrogel inclusion and delipidation. Briefly, *Biomphalaria glabrata* BRE2 individuals’ shells were removed, and snails were fixed for 24h in 4% paraformaldehyde at 4°C. Regarding infected snails, fixation was done 24h after infection with the parasite. Fixed snails were washed 3 times in Phosphate Buffered Saline solution (PBS), then they were incubated in hydrogel (from logos Biosystems: 4% v/v Acrylamide, 0.25% m/v VA-044, in PBS, no aldehydes were added in the hydrogel solution) for acrylamide penetration during 24h at 4°C. Samples were then stand in hydrogel for 3h at 37°C under vacuum for acrylamide polymerization. Finally, they are rinsed in PBS solution for 24h at 4°C before proceeding to the clearing step as such (at that stage we can leave the specimens at 4°C for a week). Clearing by lipids removal is performed actively through electrophoresis for 120h, before performing a passive step for 48h at 37°C, both steps (active and passive) use the same clearing solution composition (200 mM boric acid, 4% m/v SDS detergent, pH 8.5). Samples were washed for 24h at 37°C with PBS solution. Cleared samples are put until used in a mix solution of 80% glycerol in PBS adjusted to a RI of 1.46 using a refractometer (30PX, Mettler Toledo).

Biomphalysin 1/2 primary antibody staining was performed for 5 days at 37°C in a solution of 1% (v/v) Triton X-100. Samples are washed for 24h at 37°C in a solution of 0.1% Triton X-100 in PBS. Fluorescent labeling was performed using an Invitrogen Alexa Fluor 488 anti-rabbit antibody (ThermoFisher, A-21206). Thereafter, specimens are washed in 0.1% Triton X-100 PBS for 24h at 37°C. Nuclei were labeled using propidium iodide and samples were put in an 80% glycerol solution diluted in PBS to reach a RI of 1.46, adjusted using a refractometer (30PX, Mettler Toledo).

For full-size sample, imaging was performed using a light sheet imaging UltraMicroscope Blaze (Miltenyi Biotec) holding a 2 × MVPLAPO Olympus objective with a numerical aperture of 0.5. The imaging medium was a glycerol solution with a RI of 1.46. We used 561 nm laser and a 620/60 filter for propidium iodide imaging and a 488 nm laser and a 525/50 filter for green fluorescence detection. Z stacks were performed according to the thickness of the light sheet. Confocal imaging was performed on a Zeiss LSM 700 microscope (Bioenvironment platform, University of Perpignan, France). Pictures were treated through Imaris software for light sheet captures and with Zen (Zeiss) software and ImageJ for confocal microscopy acquisitions. For additional details and information related to this procedure, the corresponding method has been published [55].

### Transcriptional response

*B. glabrata* snails (7–9 mm in shell diameter) from Brazil strain (*Bg*BRE2) were exposed to *Escherichia coli* (Gram-negative bacteria), *Micrococcus luteus* (Gram-positive bacteria) or *Saccharomyces cerevisiae* (yeast). For 1h, snails were bathed with a concentration of 10^8^ cells/mL of each microorganism tested. Exposed individuals were then washed abundantly.

Regarding *S. mansoni* infection, each snail was put in 5 mL pond water for 6h with 10 miracidia following two conditions of infection. Either in the context of a “compatible” interaction in which *Bg*BRE2 strain was infected with *Sm*BRE parasite strain or in the context of an “incompatible” interaction in which *Bg*BRE2 strain were infected with *Sm*GH2 (originating from Guadeloupe) strain of the parasites [56,57]. In the case of a “compatible” interaction, the parasites are able to achieve their intramolluscan life cycle inside the snail host with a high prevalence and intensity while in the case of an “incompatible interaction”, parasites are unable to achieve their life cycle due to their elimination by host’s immune system [56]. For each immune challenge, 12 replicates of a pool of three snails were performed at 6, 12, 24 and 48h after exposition. Unexposed snails (control samples) were employed to assay the basal expression level of genes and were constituted by 8 replicates of a pool of 3 snails. All biological samples were stored at -80°C until use.

Extractions of total RNA from exposed and unexposed snails were carried out using the Trizol procedure (ThermoFisher Scientific, Paris, France). Digestion with DNase followed by RNA reverse transcription (4 µg of RNA) was performed using random hexamer primers belonging to the Maxima H minus first strand cDNA synthesis kit (ThermoFisher Scientific, Waltham, MA, USA). Quantitative Real Time Polymerase Chain Reaction analyses were carried out with the Roche LightCycler 480 System, performed as described in [58]. Reactions were performed using a final dilution of cDNA of 1:30 in ultrapure-water, and a final primer concentration of 0,25 µM each. The reaction was performed in No Rox SYBR Master Mix blue dTTP (Takyon Eurogentec). Cycling program was as follows: denaturation step at 95°C for 2 min, 40 cycles of amplification (denaturation at 95°C for 10 s, annealing at 60°C for 15 s, and elongation at 72°C for 22 s). A melting curve was done for each point from 65 to 97°C, with a rate of 0.11°C.s^-1^, measuring fluorescence continuously. Cycle threshold (Ct) was determined using the second derivative method of the LightCycler 480 Software release 1.5 (Roche). The mean value of Ct was calculated. Relative expression of biomphalysins was normalized to the housekeeping *S19* coding gene used as reference. A comparison was then performed between non-exposed (control) and exposed snails with the ΔΔCt method. A Shapiro-Wilks test was achieved to confront data normality. Significant differences were analyzed using a pairwise Mann-Whitney U test. Differences were considered significant and robust for when p < 0.05. Primer list is available in S18 Fig.

### Plasma recovery after hemolymph fractionation and peptide identification

*Biomphalaria glabrata* snails of Brazil (*Bg*BRE2) strain measuring 9–11 mm in diameter were collected and exposed for 6 h to 10 miracidia from Guadeloupe (*Schistosoma mansoni* GH2 -*Sm*GH2-), constituting a host-parasite incompatible interaction) or from Brazil (*Schistosoma mansoni* BRE, compatible interaction) in 5 mL of pond water. Hemolymph was then recovered from not-infected (naive), compatible or incompatible-infected individuals 48h after infection. For each condition, the hemolymph of 5 mollusks was collected, pooled and hemocytes and cell particles were centrifugated (2000 × g, 10 min, 4°C). 5 replicates were prepared this way. The supernatant (plasma) was collected, hemoglobin absorbance was measured at 413 nm through a BioTek Epoch spectrophotometer. Each sample was solubilized in reducing Laemmli buffer and migrated at a constant hemoglobin absorbance on an SDS-PAGE: hemoglobin being the most abundant plasmatic protein and its absorbance peak being 413 nm, we decided to link the volume of plasma to its absorbance at 413 nm. After a Coomassie blue staining (S7 Fig), the gel bands between 45 and 70 kDa were cut out and were placed in 10% ethanol solution before following steps. The containing proteins were digested in-gel using modified trypsin (sequencing purity, Promega), as previously described [59]. The resulting peptides were analyzed by online nanoliquid chromatography coupled to MS/MS (Ultimate 3000 RSLCnano and Q-Exactive HF, Thermofisher Scientific) using a 60 min gradient. For this purpose, the peptides were sampled on a precolumn (300 μm x 5 mm PepMap C18, Thermo Scientific) and separated in a 75 μm x 250 mm C18 column (Aurora Generation 3, 1.7µm, IonOpticks). The MS and MS/MS data were acquired using Xcalibur (version 2.9, Thermo Fisher Scientific).

Peptides and proteins were identified by Mascot (Matrix Science) through concomitant searches against the NCBI database (*Biomphalaria glabrata* taxonomy, 2021 June 29 download), a homemade database containing biomphalysin sequences, Vectorbase database (*Biomphalaria glabrata* BB02) and a homemade database containing the sequences of classical contaminant proteins found in proteomic analyses (human keratins, trypsin, etc.). Trypsin/P was chosen as the enzyme and two missed cleavages were allowed. Precursor and fragment mass error tolerances were set respectively at 10 and 20 ppm. Peptide modifications allowed during the search were: Carbamidomethyl (C, fixed), Acetyl (Protein N-term, variable) and Oxidation (M, variable). The Proline software version 2.2.0 [60] was used for the compilation, grouping, and filtering of the results (conservation of rank 1 peptides, peptide length ≥ 6 residues, false discovery rate of peptide-spectrum-match identifications < 1% [61], and minimum of one specific peptide per protein group). Proline was then used to perform a MS1 label-free quantification of the identified protein groups based on specific peptides.

Statistical analysis was then performed using the Prostar software version 1.30.5 [62]. Proteins identified in the contaminant database, proteins identified by MS/MS in less than three replicates of one condition, and proteins detected in less than four replicates of one condition were discarded. After Log_2_ transformation, abundance values were normalized by VSN (Variance Stabilizing Normalization) method before missing value imputation (slsa algorithm for partially observed values in the condition and DetQuantile algorithm for totally absent values in the condition). Statistical testing was then conducted using Limma, whereby differentially expressed proteins were sorted out using a Log_2_ (fold change) cut-off of 1 and a p-value cut-off of 0.01, leading to FDRs inferior to 5% according to the Benjamini-Hochberg estimator. Proteins found differentially abundant but identified by MS/MS in less than three replicates and detected in less than four replicates in the condition in which they were found to be more abundant were manually invalidated.

In silico trypsinization was performed on both peptide sequences of biomphalysin 1 and biomphalysin 2 (NCBI and Vectorbase) to check the potential non-detectable areas of the protein during mass analysis. This was performed with expasytool (https://web.expasy.org/peptide_mass/), selected enzyme is Trypsin, 2 missed cleavages were allowed and peptides displayed were in a range from 750 Da to 4000 Da (in comparison, the mass spectrometry method applied didn’t allow to identify peptides under a mass of 800 Da).

### Plasma-cell interaction

Miracidia of *S. mansoni* from NMRI strain were hatched in fresh water and put in culture in trehalose-depleted CBSS for 48 hours. Eggs of *Fasciola hepatica* were put in fresh water in the dark for 20 days for proper development and exposed to light to trigger miracidia hatching. HeLa cells were maintained at 37°C in 10% FBS L-15 Glutamax culture medium at atmospheric CO_2_. BCL2 Jurkat cells were maintained in RPMI-1640 and C2C12 cells were maintained in DMEM, all culture media were supplemented with 10% FBS and L-glutamine, culture was performed at 37°C under a 5% CO2 enriched atmosphere. For the analysis in western blot, each cell type was exposed to 200 µL of ultracentrifuged plasma or kept in CBSS (control condition) for 2 hours at room temperature (21°C). For fluorescence microscopy observations, sporocysts and HeLa cells were exposed for 1 hour. After exposure, plasma was discarded, and cells were gently rinsed in glucose-trehalose-depleted CBSS three times at room temperature to discard remaining plasma. Water in which *Fasciola* eggs hatched was also exposed to plasma to check for a potential water effect (no rinsing here). To obtain ultracentrifuged plasma, hemolymph was collected and pooled from *Bg*BRE2 strain and centrifuged at 2000 × g for 10 min at 4°C to eliminate hemocytes. The plasmatic supernatant was then ultracentrifuged at 50 000 rpm for 2 hours at 4°C to eliminate hemoglobin (Optima L-90K Ultracentrifuge from Beckman Coulter using a SW 55 Ti rotor).

Then, each condition was put in reducing Laemmli buffer for denaturation and SDS-PAGE. Migration was performed in a 7.5% and in a 4–20% gradient gel and western blotting was then performed after PVDF membrane transfer using anti-biomphalysin 1 antibody, revealed using anti-rabbit HRP-tagged antibody. Actin was used as control of protein quantity load using anti-actin antibody (ref. MA1-744, Invitrogen). HeLa, C2C12 and BCL2 Jurkat cells exposed conditions were run on the same 4–20% gradient gel. A total protein dosage using 2D-Quant kit (Cytiva, GE80-6483-56) was performed after cell sonication in a lysis buffer (50 mM Tris, 150 mM NaCl, 1% NP-40, 5 mM EDTA), 10 cycles of 30 sec each at 70% intensity at 4°C were performed using a GM mini20 Sonoplus (Bandelin electronics) and a MS1.5 probe.

For confocal microscopy imaging, sporocysts were fixed in 0.4% paraformaldehyde for 10 minutes and observed through a Zeiss LSM 700 microscope. After an exposure time of 4 hours, 1:2 Plasma dilution or 1:4 Plasma dilution using CBSS, HeLa cells were fixed in 4% paraformaldehyde for 10 minutes, B1/2 was labeled using anti-B1/2 antibody and a secondary Alexa-488 coupled antibody, nuclei were stained with DAPI and A633-coupled phalloidin (Abcam, ref. A22284) was used to highlight F-actin.

### Structural features of biomphalysins

Peptide sequences of the 23 biomphalysins, after removal of the signal peptide sequence, were given as input in AlphaFold2 – ColabFold v1.5.3 [63] either with or without last 50–55 unfolded C-terminal residues. No relaxation of the predicted models was performed as comparison of relaxed and unrelaxed models for different biomphalysins showed no difference in RMSD value. Predictions were made without any imposed reference, as it was done previously [18].

Predicted structures were pairwise superimposed, and Root Mean Square deviation (RMSD) for Ca atoms were computed using ChimeraX matchmaker tool for global structure (all residues), small lobe (residues 1 to 165), large lobe (residues 190 to the end), Nter region (residues 1 to 252 and 392 to 475) or Cter region (residues 253 to 391 and 476 to the end), with default parameters.

Distance between sulphur atoms in cysteines was measured in ChimeraX. Biomphalysin sequences were pairwise-aligned in EMBL-EBI Clustal Omega multiple alignment tool [64] and final representation was done with ESPript3 webserver [65].

## Supporting information

S1 Fig Biomphalysin 1/2 distribution in *Biomphalaria glabrata* different organs through immunohistology. A, B: Heart; C, D: Hepatopancreas; E, F: Ovotestis; G, H: Stomach and albumen gland. The HES-stained cuts (A, C, E, G) permit the identification of the immunolabeled tissues. The immunolabeled cuts (B, D, F, H) are stained with the anti-B1/2 antibody revealed through an Alexa 594-labeled secondary antibody. No fluorescence is observed for hepatopancreas, ovotestis, stomach and albumen gland (except in Fig 2 where a structure defined as a hemolymphatic vessel is positive inside the albumen gland). Ac: Acinus, AG: Albumen Gland, Au: Auricle, Lu: Lumen, Lo: Lobule, P: Pericard, STO: Stomach, V: Ventricle.
(TIF)

S2 Fig Control western blotting of the anti-B1/2 antibody. The western blot was performed on different plasma samples (1–4) on a 12% gel. A: Anti-B1/2 antibody. B: Same membrane than in A, using the anti-B1/2 antibody pre-incubated with the antigenic peptide (molar ratio of 1:20, antibody:peptide).
(TIF)

S3 Fig Negative controls for anti-B1/2 and anti-TEP1 antibody in immunohistology and immunocytology experiments. A and B: Pre-immune serum from the rabbit immunized against the B1/2 peptide was used at the same dilution as for the immunohistology experiment (1/750). C and D: Anti-B1 antibody was inhibited with the antigenic peptide at a 1:10 (antibody:peptide) molar ratio and then used as a primary antibody for the immunolabeling on those cuts. E: The secondary anti-rabbit antibody was used alone (without the anti-B1/2 antibody) on *Bg*BS-90 hemocytes, in the same experimental conditions as in Fig 3. F: The secondary anti-rat antibody was used alone (without the anti-TEP1 antibody) on *Bg*BS-90 hemocytes, in the same experimental conditions as in Fig 3.
(TIF)

S4 Fig Biomphalysin 1 distribution in *B. glabrata* hemocytes (*Bg*BRE2 strain) through immunocytology. A, B: Overview of the general population of hemocytes after immunolabeling. Hemocytes of *Bg*BRE2 strain. C, D: The same type of subpopulation is revealed after the labeling, small round cells, unstained by phalloidin (D). Hemocytes of *Bg*BRE2 strain. The same labeling protocol as Fig 3 was applied. Green: Alexa488 coupled secondary antibody (B1/2), Red: Alexa594 coupled phalloidin, Blue: 4′,6-diamidino-2-phenylindole. Yellow arrows: B1/2-positive cells.
(TIF)

S5 Fig Rotation of the foot of a tissue-cleared *B. glabrata* specimen immunolabeled for B1/2. Red: propidium iodide, green: immunolabeling of B1/2. This specimen corresponds to the Fig 4B and 4C specimen.
(AVI)

S6 Fig Expression of biomphalysin genes in response to microorganism exposures. qRTPCR were performed on whole snail organisms exposed either to *Escherichia coli*, *Micrococcus luteus*, *Saccharomyces cerevisiae* and *Schistosoma mansoni* compared to unexposed snails. Expression was measured at four time points (6, 12, 24 and 48 hours after exposure) and was normalized to S19 housekeeping gene expression and compared with the expression obtained in non-exposed snails. Relative expression ratio: 2-ΔΔCT. No signal was obtained for B5, B6, B7, B16 and B19.
(TIF)

S7 Fig Optical microscopy observation (white light) of the effects of an exposure to ultracentrifuged plasma from naive *B. glabrata* on three mammal cell lines. A: Human cervix cancer HeLa cells exposed to CBSS. B: HeLa cells exposed to ultracentrifuged plasma. C: BCL2-Jurkat cells (TCD4 human lymphoma) exposed to CBSS. D: BCL2-Jurkat cells exposed to ultracentrifuged plasma. E: C2C12 cells (mouse myoblastic cells) exposed to CBSS. F: C2C12 cells exposed to ultracentrifuged plasma. The only one of these cell lines to be recognized by B1/2 is the HeLa cell line (shown in Fig 6). These cells are the only ones to immediately be lysed following plasma exposure (< 2 min). After immediate lysis, the aspect of HeLa cells remains the same at the end of 20 min (B). No cell lysis is observed in CBSS condition during the experience despite the low osmolarity of CBSS (about 100 mOsm.L^-1^). Final exposure time: 20 min. Scale bar: 50 µm.
(TIF)

S8 Fig Coomassie blue staining of SDS-PAGE before (A) and after (B) band cuts for mass analyses. BN: Naive *Biomphalaria Bg*BRE2 strain. BB: *Bg*BRE2 snail infected by *Sm*BRE strain. BG: *Bg*BRE2 snail infected by *Sm*GH2 strain.
(TIF)

S9 Fig List of the identified peptides following mass spectrometry analyses for biomphalysin 1 and 2. a: Peptides only matched on NCBI database. b: Peptides only matched on Vectorbase database. Bold: Specific peptides for either Biomphalysin 1 or Biomphalysin 2. Note: The peptide « CTKPANHPLNYGNCQDIDVSSCMGRK » was identified but the number of collected spectra was not sufficient for a proper quantification.
(XLSX)

S10 Fig List of peptides obtained after in silico trypsinization of biomphalysin 1 and 2 sequences from either Vectorbase (BGLB000137 or BGLB000033) or from the 2021 B1 and B2 identified sequences [18]. A: Peptide list. #MC: Missed Cleavage(s) (0 in black, 1 in blue, 2 in red). B: Sequence theoretical coverage for the 4 sequences, respectively BGLB000137, B1, BGLB000033 and B2. Amino acids in bold and capital letters: areas covered by *in silico* obtained peptides (A).
(XLSX)

S11 Fig Structure predictions for all the biomphalysin proteins and small lobe sequence homology. A: Structure predictions of the 23 biomphalysins. Predictions colored in tan were done with the last C-terminal residues and the blue ones without it. Groups are based on the phylogenetic analysis previously done [18] and respect the same color code: B1 to B8 are in the tan box, B11 to B18 are in the green box, B19 to B23 are in the pink box, B9 is the magenta box and B10 is in the purple box. Superimposition of the different predictions was done with ChimeraX matchmaker tool, without imposing any reference region. B: Small lobe sequence homology between the 23 biomphalysins. Domain I sequence identity and sequence similarity were calculated with Clustal Omega alignment tool online [64].
(TIF)

S12 Fig Biomphalysin 1 cysteine position in the small lobe and conservation among biomphalysins. The sequence alignment of all 23 biomphalysins was performed using the Clustal Omega and ESPript 3.0 web servers. Conserved residues are highlighted in red, and similar residues are indicated by a blue frame. The arrows above the alignment indicate the location of the conserved cysteines. The color code by pair corresponds to neighboring cysteines observed in the AlphaFold2 predicted model of biomphalysin 1. The table below the sequence alignment uses the same color code to indicate the number of the cysteines, and the distance measured between neighboring cysteines.
(TIF)

S13 Fig 3D predicted structures of biomphalysin 1 (A), soluble form of aerolysin (B) and aerolysin protomer at the prepore state (C) [66]. From left to right: the predicted biomphalysin 1 model by AlphaFold2 (A); the aerolysin protomer structure in soluble form (PDB: 1PRE) (B); and the aerolysin protomer structure in the prepore state (PDB: 5JZH) (C). The aerolysin small lobe (residues 2–396) has been removed from both B and C structures to lighten the representation. Rainbow coloring from blue to red shows the topology of the three representations from the N- to the C-terminus.
(TIF)

S14 Fig Topological representation of the large lobe of biomphalysin 1 predicted structure. β-strands are depicted with arrows and α helices with cylinders. This representation reuses the same color code used by Podobnik *et al.* [49] and follows the consensus from Szczesny *et al.* [7]. Red: variable loop, blue: β-strands of the conserved aerolysin core, cyan: insertion loop. Correspondence to the work of Podobnik et *al.* [49]: s15 corresponds to β1, s16 corresponds to β2, s19 corresponds to β3, s20 corresponds to β4, s22 corresponds to β5.
(TIF)

S15 Fig Comparison of the RMSD values for AlphaFold biomphalysin predictions. For the two sets of predictions (non-mature and Cter-mature), biomphalysins were compared two by two with ChimeraX matchmaker tool, for different regions in the proteins. When comparing Cα RMSD from ChimeraX matchmaker tool, 89% of the values for small lobe comparison are under 2.0 Å, while only 8% of the values when large lobes are compared (S11 Fig). This indicates very similar/conserved structure organization of all domains I/ small lobes, despite very relatively low sequence identity (15% of values are above 60% identity -S11B Fig). Large lobe organization is conserved, but its orientation changes between each prediction and affects global and large lobe RMSD. Removal of unfolded C-terminal residues also modify the orientation: RMSD are decreased, most of the biomphalysins seem more aligned along a vertical axis. This observation can be an artifact of AlphaFold predictions or a structural rearrangement necessary for the activity. Biomphalysin 20 is a particular case: it is the protein with the highest RMSDs, followed by biomphalysins 19 and 23. In the transmembrane region, there is a long α-helix instead of a loop and the insertion loop is positioned on the other side of the large lobe compared to other predictions. It is also the case for biomphalysin 19, but only when the prediction includes the unfolded C-terminal residues. Either biomphalysin 20 shape is a prediction artifact or a specific structural organization linked to its function.
(XLSX)

S16 Fig Accession numbers for biomphalysin protein sequences (used in [18]) and chromosomal gene positions on the xgBioGlab47.1 genome assembly. Some sequence differences can be noticed between biomphalysin genes originating from the genome assembly and previously defined sequences.
(XLSX)

S17 Fig Clustalω multiple alignment of the antigenic peptide and biomphalysin protein sequences. The protein sequences from BB02 database, xgBioGlab47.1 and Vectorbase (“BGLB” name code) are shown and aligned. The antigenic peptide used to produce anti-B1/2 antibody is “INALDRNDVNWADDA”. The multiple alignment was performed with the BioEdit software [54].
(PDF)

S18 Fig Primer list and sequences used for qRTPCR analyses. (XLSX)

S19 Fig Raw Ct data for qRTPCR analyses shown in Fig 5.
(XLSX)

S20 Fig Raw Ct data for qRTPCR analyses shown in S6 Fig. (XLSX)
