# Supplementary figures and images for: The structural features and immunological role of biomphalysins in the snail Biomphalaria glabrata
Source: PLoS Pathog. 2025 Jun 24;21(6):e1013225. doi: 10.1371/journal.ppat.1013225 (PMC12186885; doi:10.1371/journal.ppat.1013225)

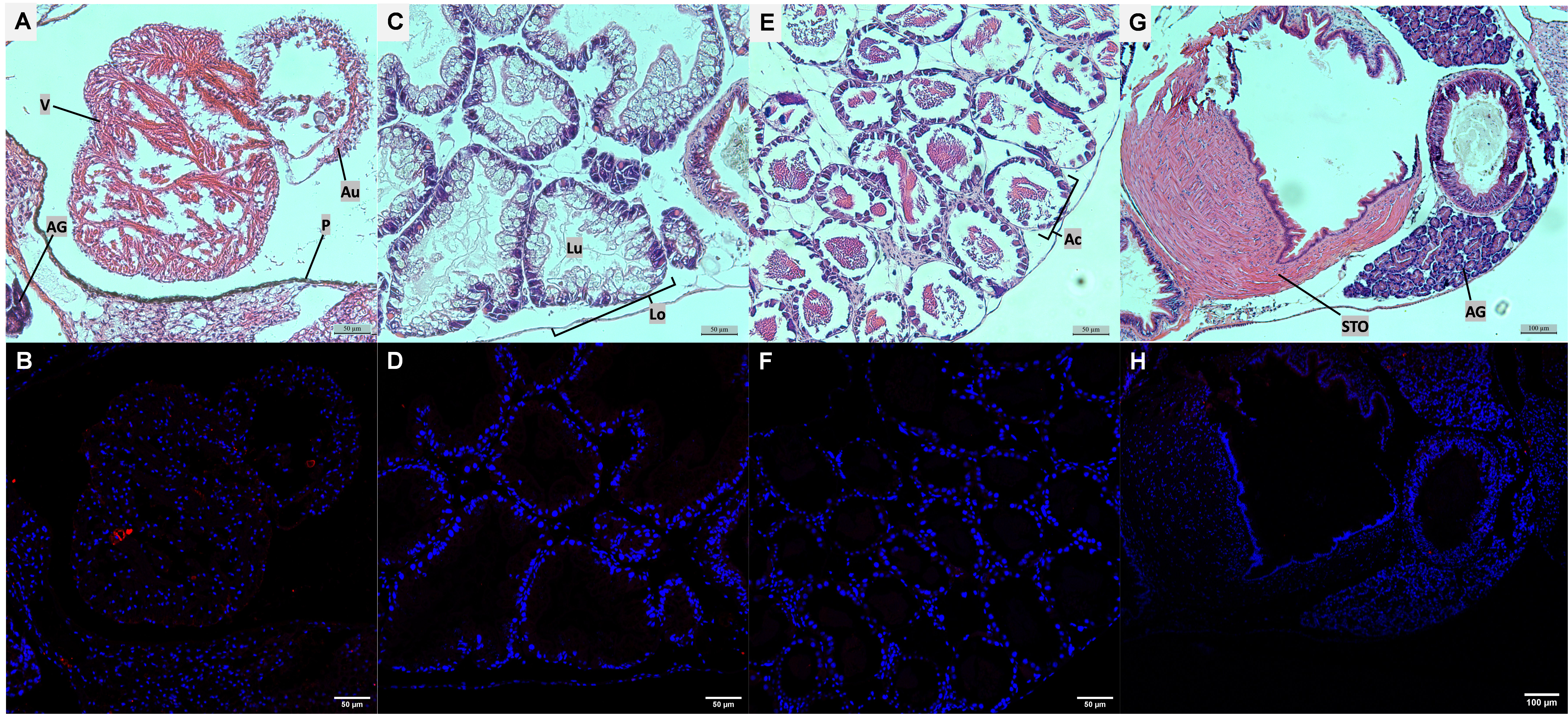

Supplement: S1 Fig — A, B: Heart; C, D: Hepatopancreas; E, F: Ovotestis; G, H: Stomach and albumen gland. The HES-stained cuts (A, C, E, G) permit the identification of the immunolabeled tissues. The immunolabeled cuts (B, D, F, H) are stained with the anti-B1/2 antibody revealed through an Alexa 594-labeled secondary antibody. No fluorescence is observed for hepatopancreas, ovotestis, stomach and albumen gland (except in Fig 2 where a structure defined as a hemolymphatic vessel is positive inside the albumen gland). Ac: Acinus, AG: Albumen Gland, Au: Auricle, Lu: Lumen, Lo: Lobule, P: Pericard, STO: Stomach, V: Ventricle. (TIF) [file ppat.1013225.s001.tif]

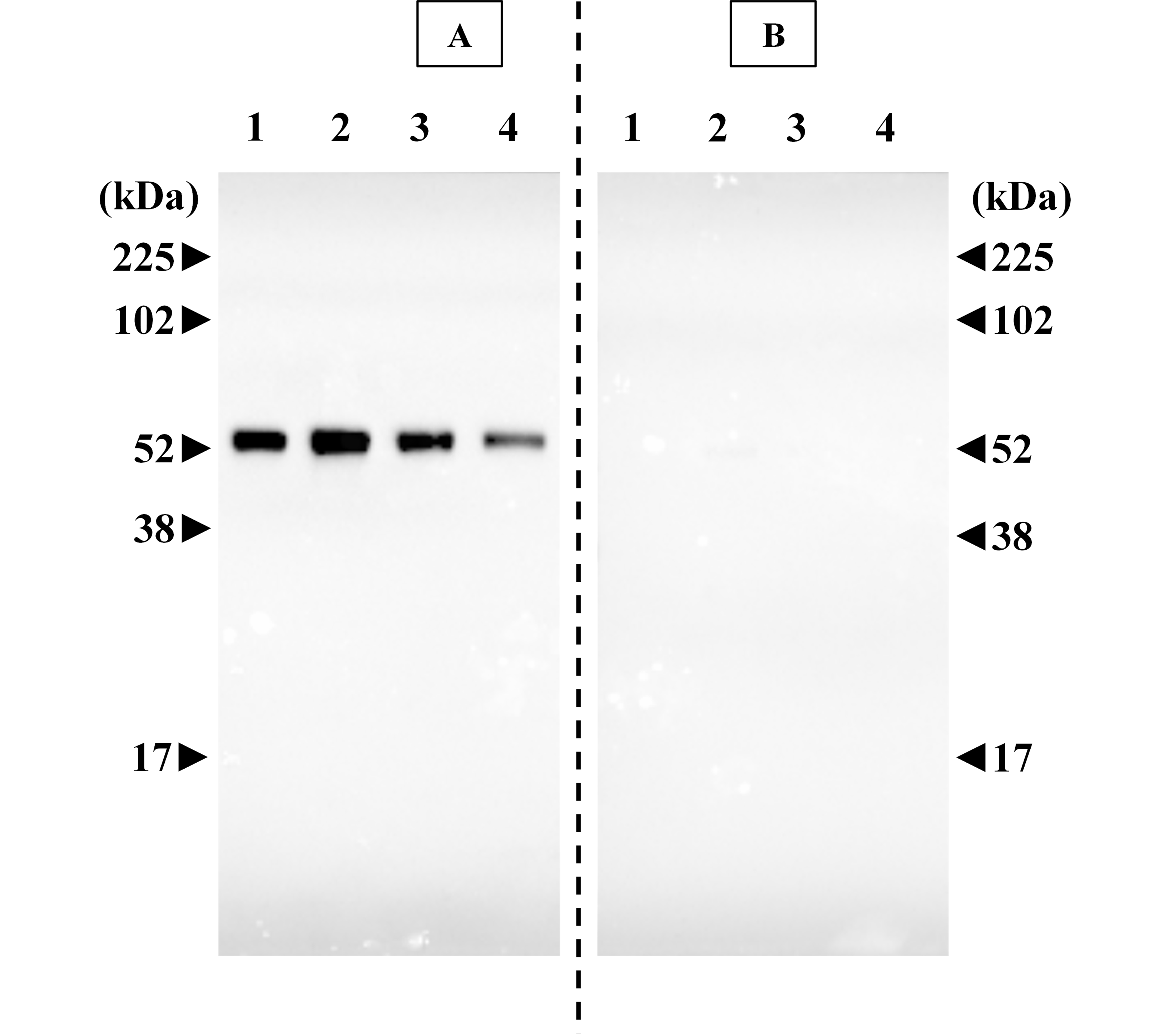

Supplement: S2 Fig — The western blot was performed on different plasma samples (1–4) on a 12% gel. A: Anti-B1/2 antibody. B: Same membrane than in A, using the anti-B1/2 antibody pre-incubated with the antigenic peptide (molar ratio of 1:20, antibody:peptide). (TIF) [file ppat.1013225.s002.tif]

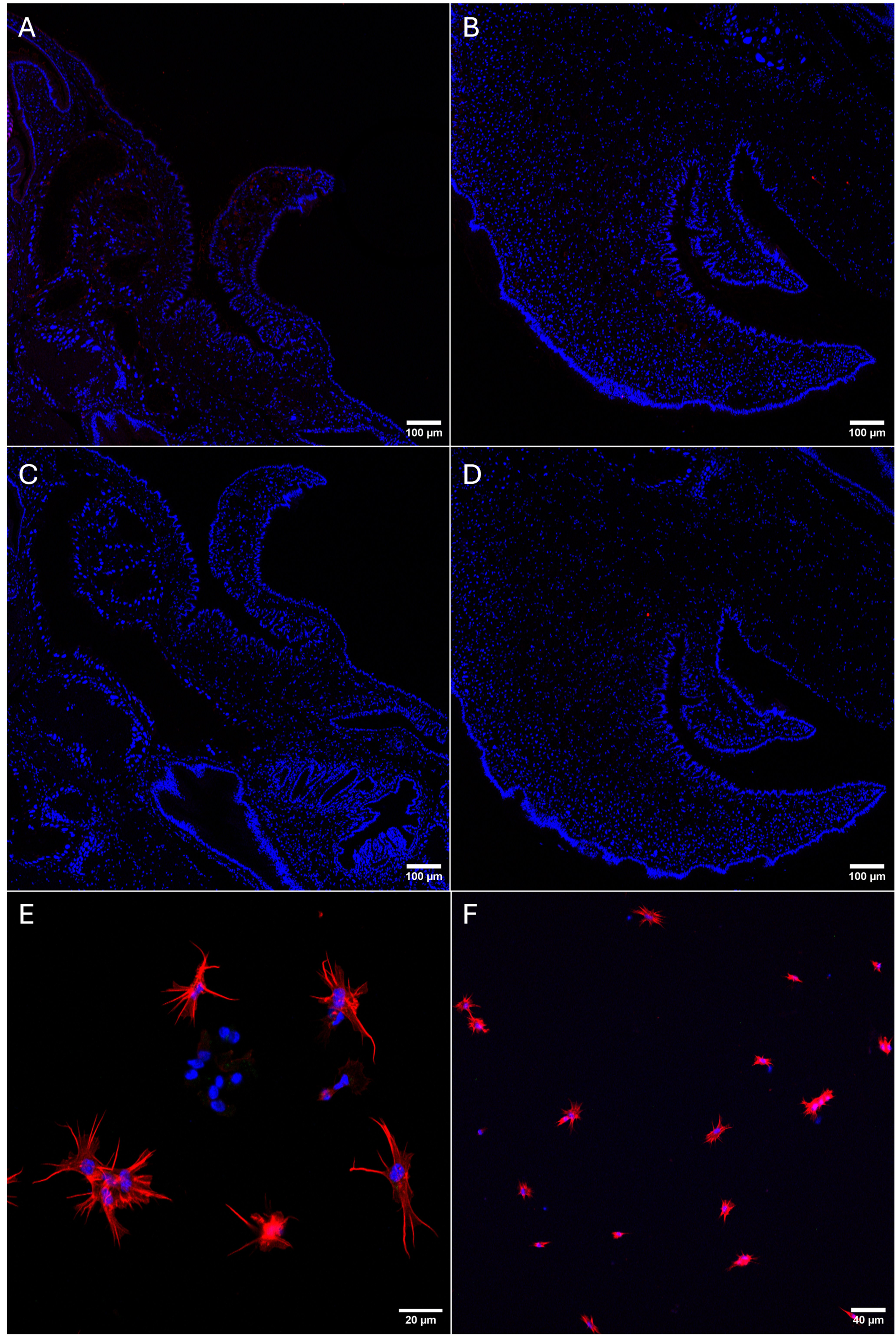

Supplement: S3 Fig — A and B: Pre-immune serum from the rabbit immunized against the B1/2 peptide was used at the same dilution as for the immunohistology experiment (1/750). C and D: Anti-B1 antibody was inhibited with the antigenic peptide at a 1:10 (antibody:peptide) molar ratio and then used as a primary antibody for the immunolabeling on those cuts. E: The secondary anti-rabbit antibody was used alone (without the anti-B1/2 antibody) on BgBS-90 hemocytes, in the same experimental conditions as in Fig 3. F: The secondary anti-rat antibody was used alone (without the anti-TEP1 antibody) on BgBS-90 hemocytes, in the same experimental conditions as in Fig 3. (TIF) [file ppat.1013225.s003.tif]

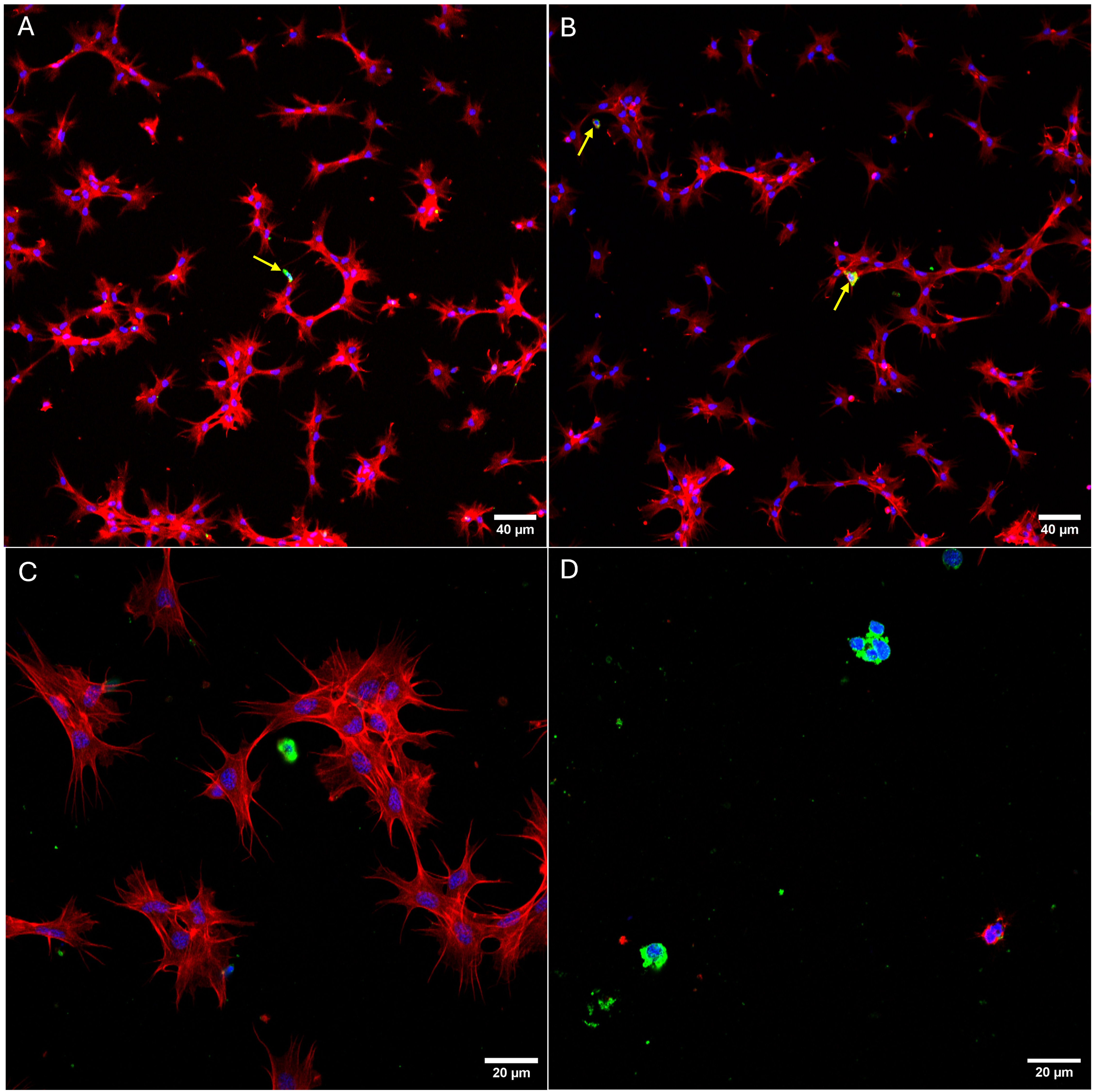

Supplement: S4 Fig — A, B: Overview of the general population of hemocytes after immunolabeling. Hemocytes of BgBRE2 strain. C, D: The same type of subpopulation is revealed after the labeling, small round cells, unstained by phalloidin (D). Hemocytes of BgBRE2 strain. The same labeling protocol as Fig 3 was applied. Green: Alexa488 coupled secondary antibody (B1/2), Red: Alexa594 coupled phalloidin, Blue: 4′,6-diamidino-2-phenylindole. Yellow arrows: B1/2-positive cells. (TIF) [file ppat.1013225.s004.tif]

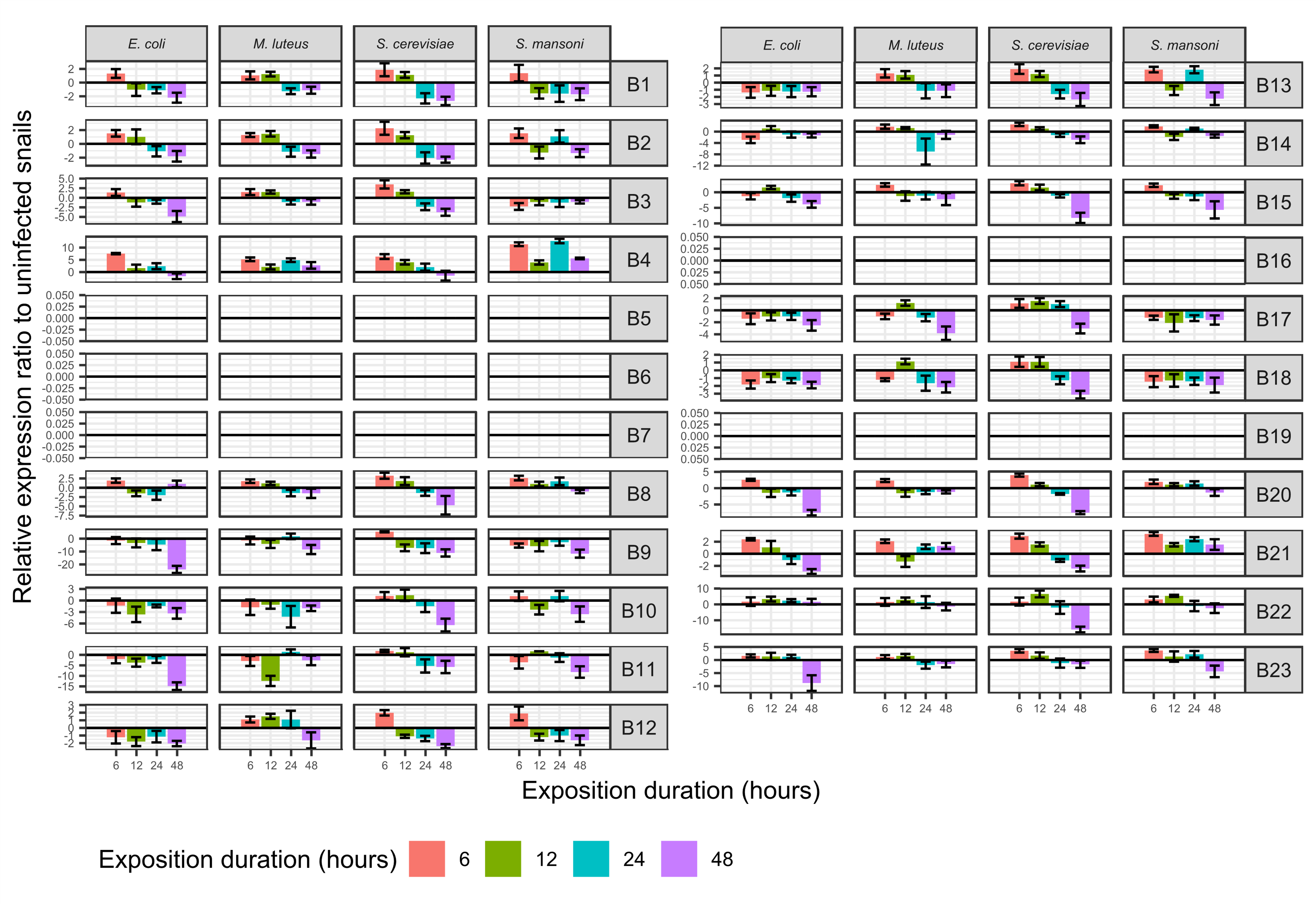

Supplement: S6 Fig — qRTPCR were performed on whole snail organisms exposed either to Escherichia coli, Micrococcus luteus, Saccharomyces cerevisiae and Schistosoma mansoni compared to unexposed snails. Expression was measured at four time points (6, 12, 24 and 48 hours after exposure) and was normalized to S19 housekeeping gene expression and compared with the expression obtained in non-exposed snails. Relative expression ratio: 2-ΔΔCT. No signal was obtained for B5, B6, B7, B16 and B19. (TIF) [file ppat.1013225.s006.tif]

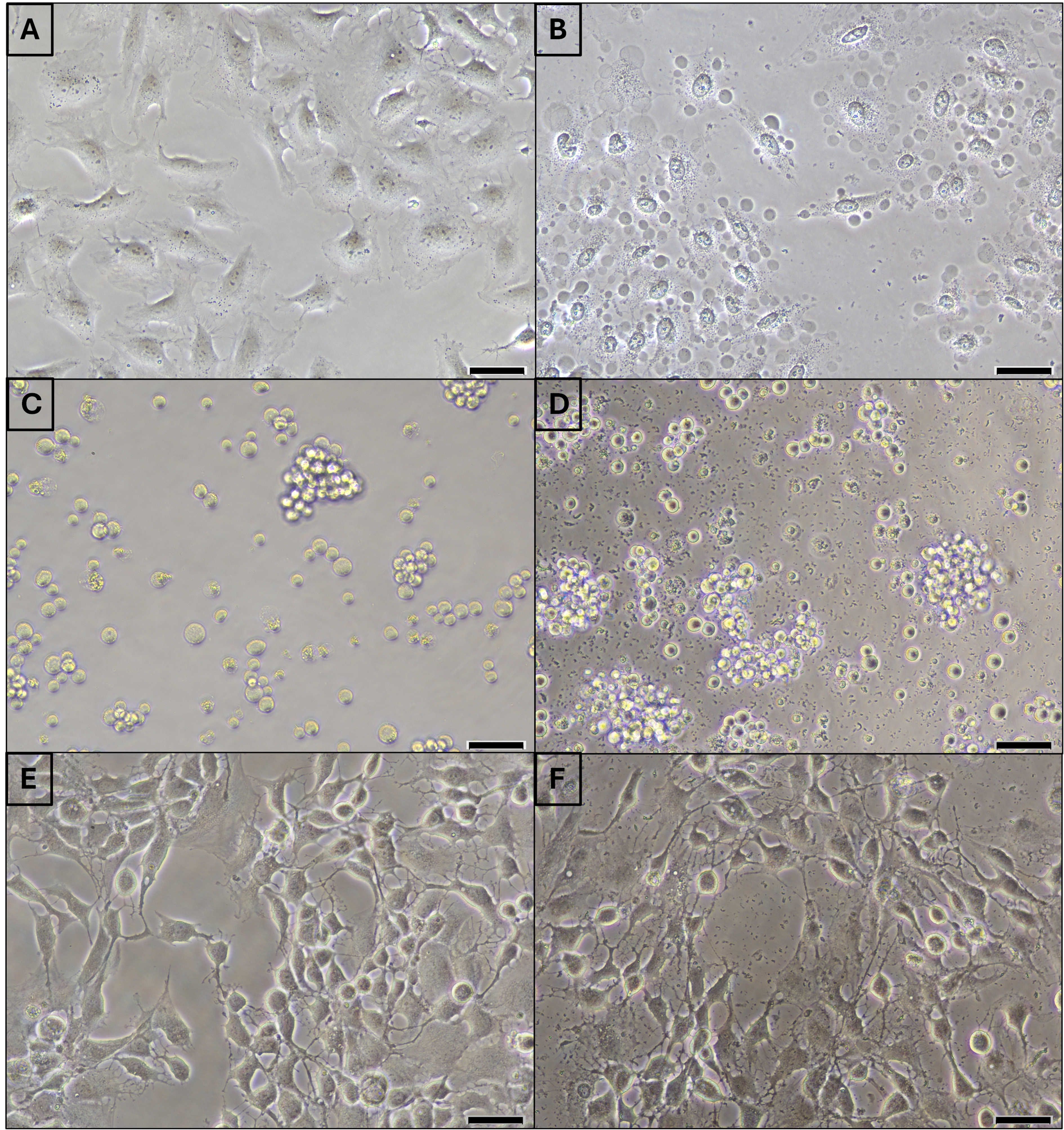

Supplement: S7 Fig — A: Human cervix cancer HeLa cells exposed to CBSS. B: HeLa cells exposed to ultracentrifuged plasma. C: BCL2-Jurkat cells (TCD4 human lymphoma) exposed to CBSS. D: BCL2-Jurkat cells exposed to ultracentrifuged plasma. E: C2C12 cells (mouse myoblastic cells) exposed to CBSS. F: C2C12 cells exposed to ultracentrifuged plasma. The only one of these cell lines to be recognized by B1/2 is the HeLa cell line (shown in Fig 6). These cells are the only ones to immediately be lysed following plasma exposure (< 2 min). After immediate lysis, the aspect of HeLa cells remains the same at the end of 20 min (B). No cell lysis is observed in CBSS condition during the experience despite the low osmolarity of CBSS (about 100 mOsm.L-1). Final exposure time: 20 min. Scale bar: 50 µm. (TIF) [file ppat.1013225.s007.tif]

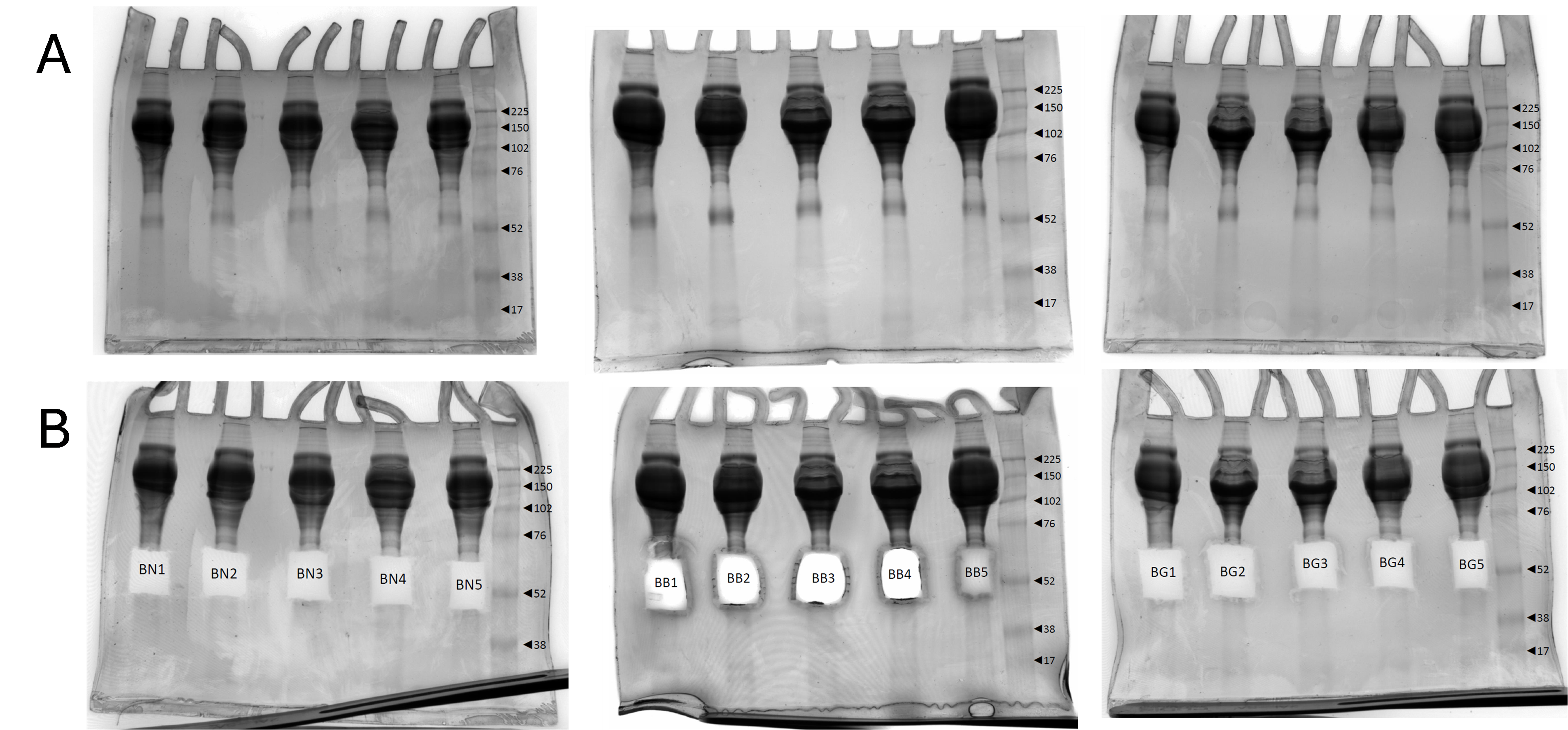

Supplement: S8 Fig — BN: Naive Biomphalaria BgBRE2 strain. BB: BgBRE2 snail infected by SmBRE strain. BG: BgBRE2 snail infected by SmGH2 strain. (TIF) [file ppat.1013225.s008.tif]

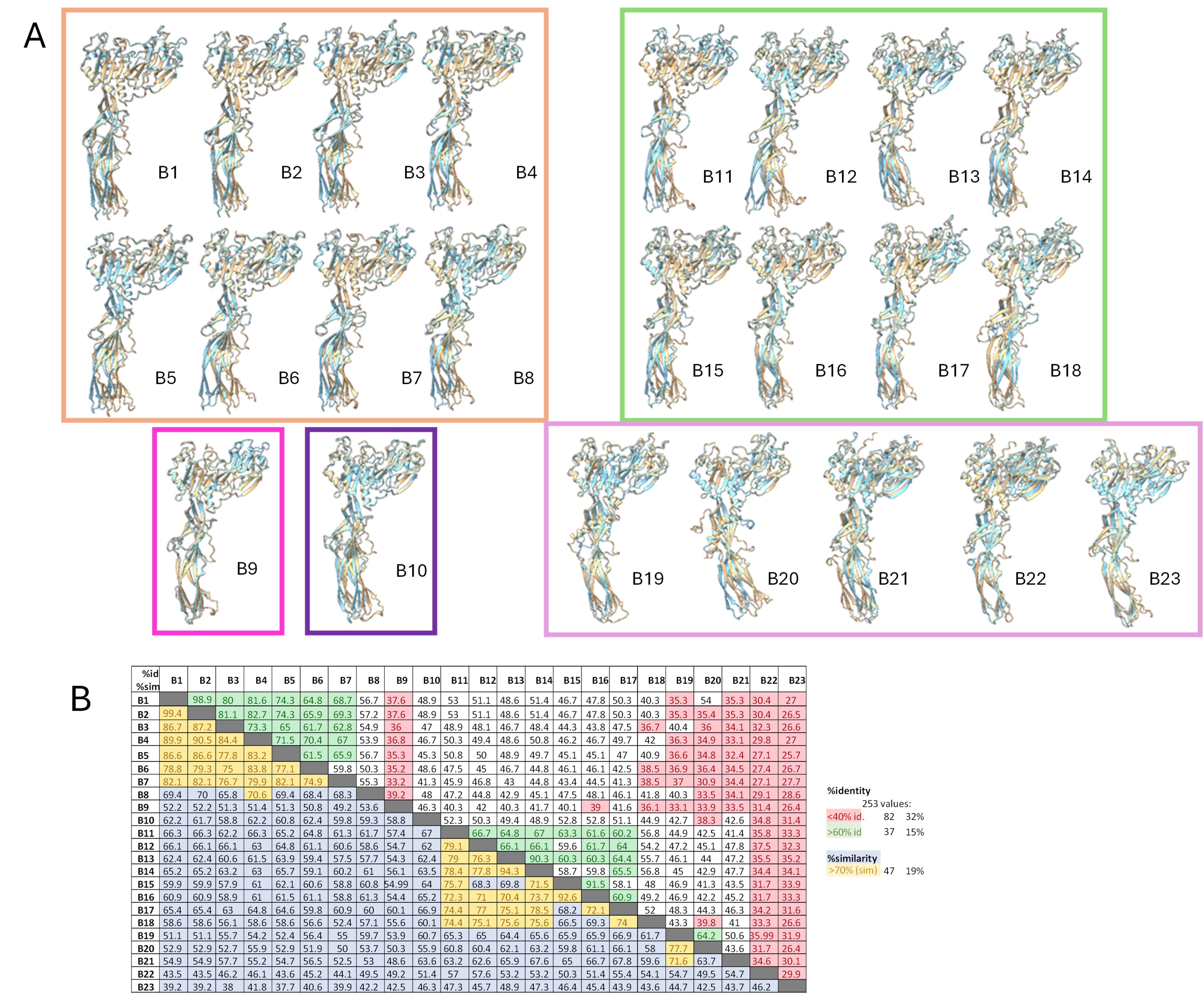

Supplement: S11 Fig — A: Structure predictions of the 23 biomphalysins. Predictions colored in tan were done with the last C-terminal residues and the blue ones without it. Groups are based on the phylogenetic analysis previously done [18] and respect the same color code: B1 to B8 are in the tan box, B11 to B18 are in the green box, B19 to B23 are in the pink box, B9 is the magenta box and B10 is in the purple box. Superimposition of the different predictions was done with ChimeraX matchmaker tool, without imposing any reference region. B: Small lobe sequence homology between the 23 biomphalysins. Domain I sequence identity and sequence similarity were calculated with Clustal Omega alignment tool online [64]. (TIF) [file ppat.1013225.s011.tif]

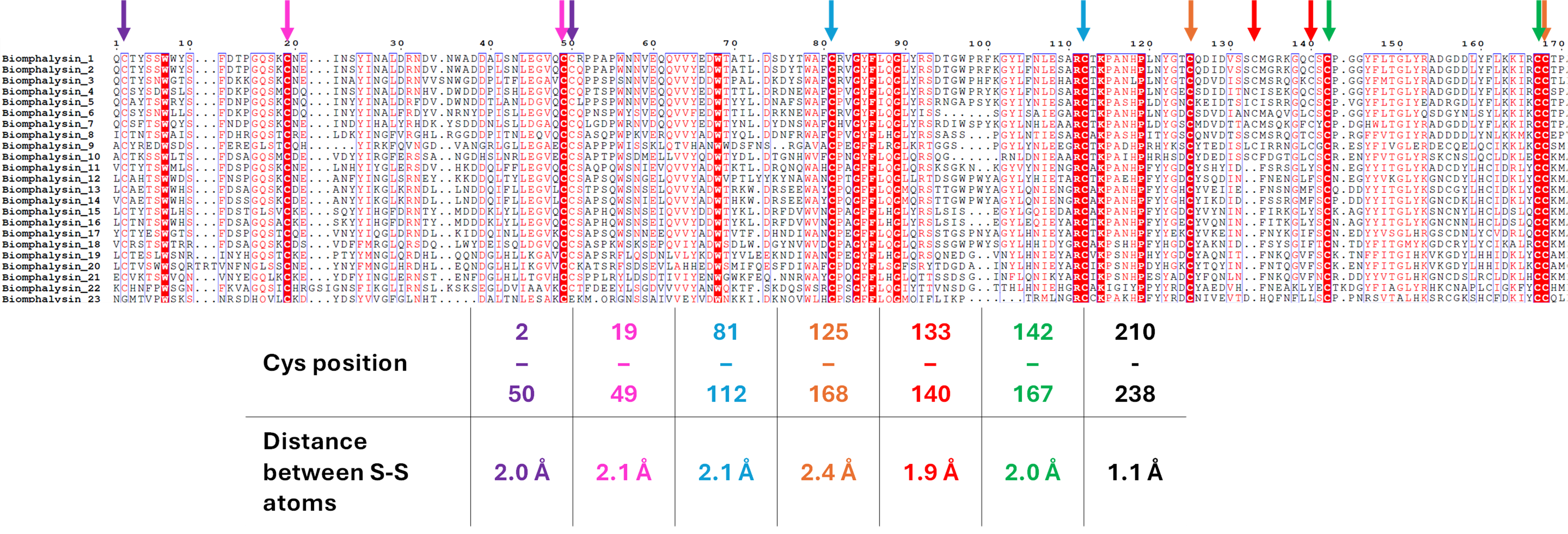

Supplement: S12 Fig — The sequence alignment of all 23 biomphalysins was performed using the Clustal Omega and ESPript 3.0 web servers. Conserved residues are highlighted in red, and similar residues are indicated by a blue frame. The arrows above the alignment indicate the location of the conserved cysteines. The color code by pair corresponds to neighboring cysteines observed in the AlphaFold2 predicted model of biomphalysin 1. The table below the sequence alignment uses the same color code to indicate the number of the cysteines, and the distance measured between neighboring cysteines. (TIF) [file ppat.1013225.s012.tif]

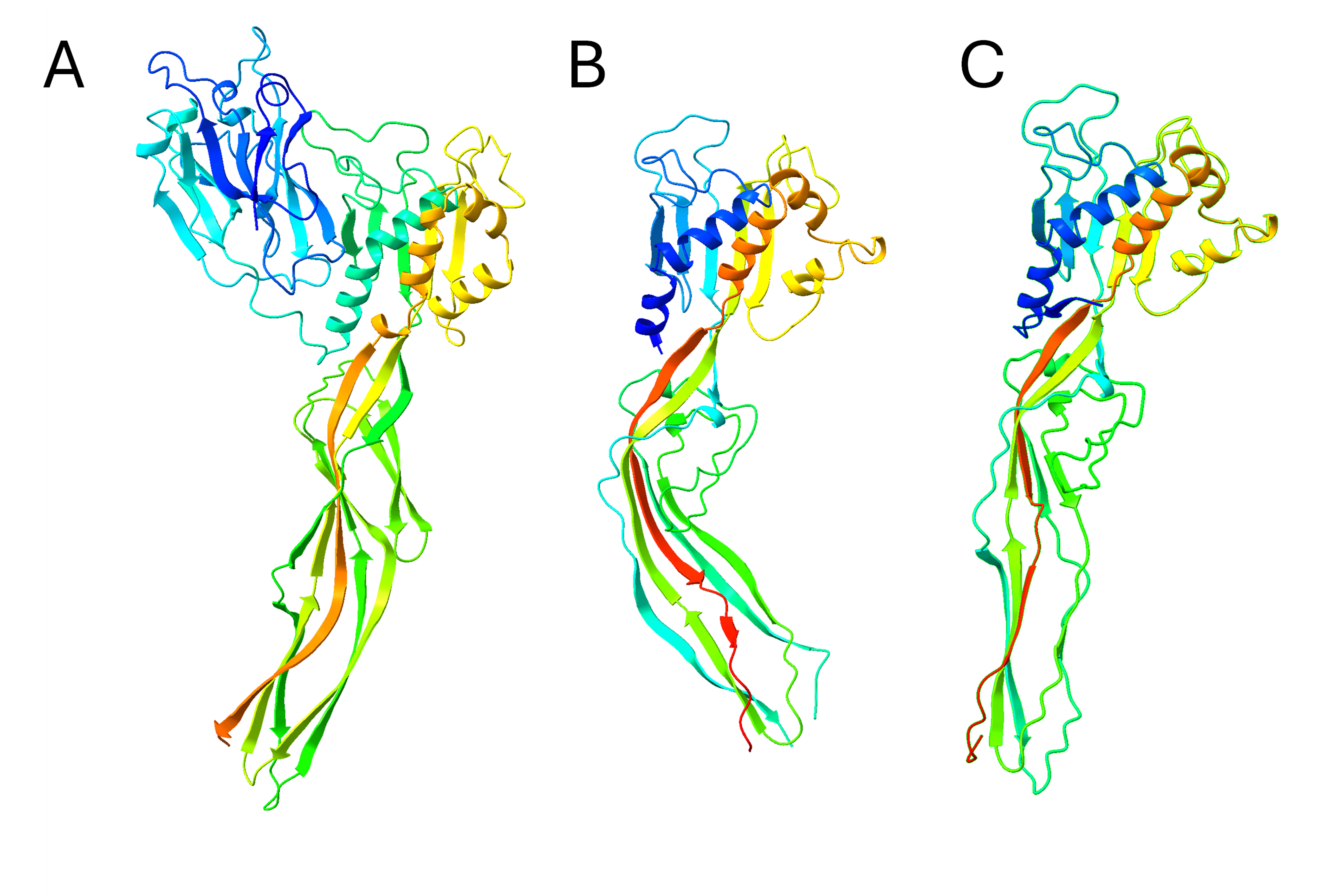

Supplement: S13 Fig — From left to right: the predicted biomphalysin 1 model by AlphaFold2 (A); the aerolysin protomer structure in soluble form (PDB: 1PRE) (B); and the aerolysin protomer structure in the prepore state (PDB: 5JZH) (C). The aerolysin small lobe (residues 2–396) has been removed from both B and C structures to lighten the representation. Rainbow coloring from blue to red shows the topology of the three representations from the N- to the C-terminus. (TIF) [file ppat.1013225.s013.tif]

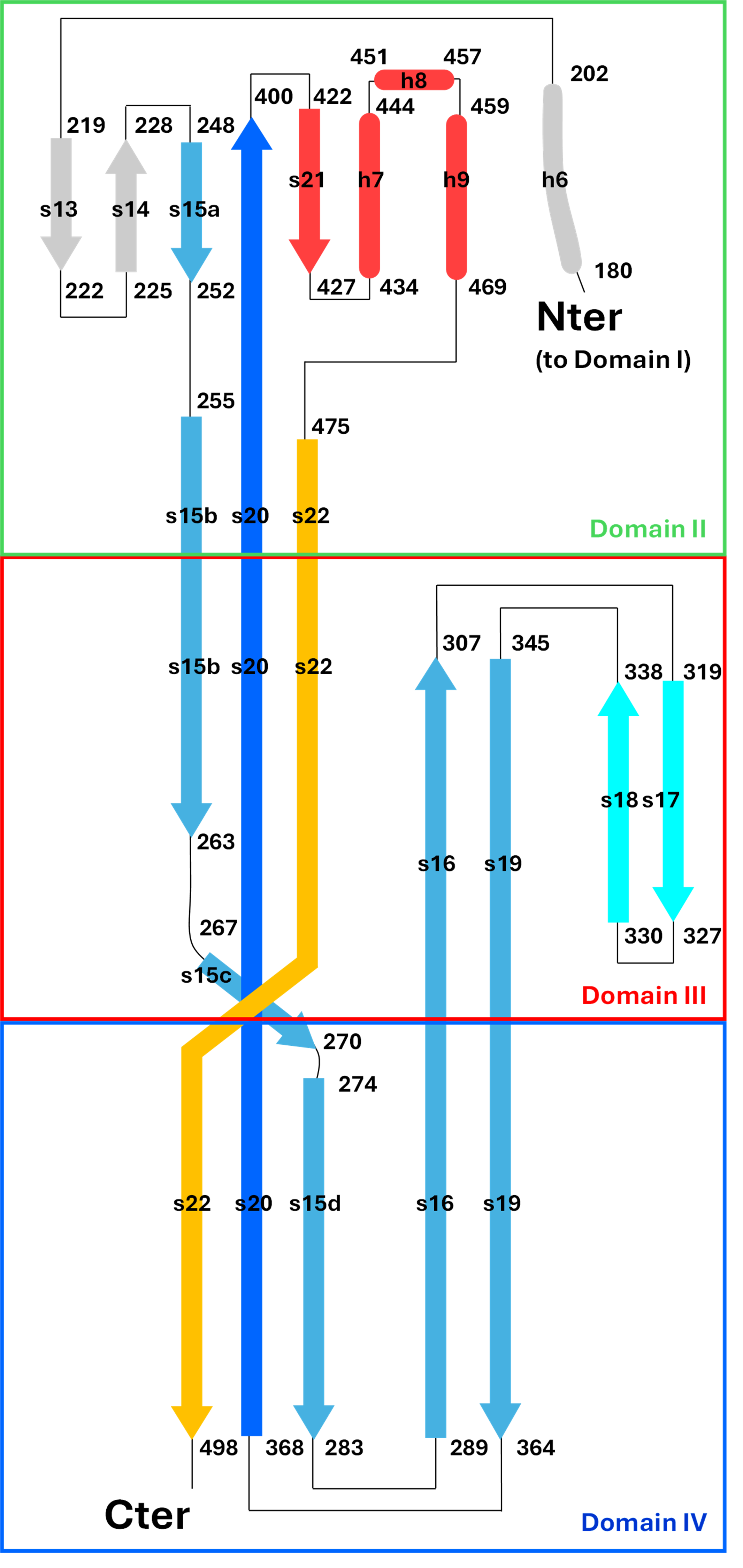

Supplement: S14 Fig — β-strands are depicted with arrows and α helices with cylinders. This representation reuses the same color code used by Podobnik et al. [49] and follows the consensus from Szczesny et al. [7]. Red: variable loop, blue: β-strands of the conserved aerolysin core, cyan: insertion loop. Correspondence to the work of Podobnik et al. [49]: s15 corresponds to β1, s16 corresponds to β2, s19 corresponds to β3, s20 corresponds to β4, s22 corresponds to β5. (TIF) [file ppat.1013225.s014.tif]
