# Supplementary material for: The structural features and immunological role of biomphalysins in the snail Biomphalaria glabrata
Source: PLoS Pathog. 2025 Jun 24;21(6):e1013225. doi: 10.1371/journal.ppat.1013225 (PMC12186885; doi:10.1371/journal.ppat.1013225)
Supplement: S17 Fig — The protein sequences from BB02 database, xgBioGlab47.1 and Vectorbase (“BGLB” name code) are shown and aligned. The antigenic peptide used to produce anti-B1/2 antibody is “INALDRNDVNWADDA”. The multiple alignment was performed with the BioEdit software [54]. (PDF) [file ppat.1013225.s017.pdf]

```
                                    10         20         30         40         50         60         70         80         90        100        110        120        130        140        150        160        170
B1-Antibody_PEP       1   ---------- ---------- ---------- ---------- ---------I NALDRNDVN- WADDA----- ---------- ---------- ---------- ---------- ---------- ---------- ---------- ---------- ---------- ----------  15  B1-Antibody_PEP
xgBioGlab47.1-B1      1   ---MFLQIFV AVTLVQYVSS Q-CTYSSWWY S---FDTPGQ SKCNEINSYI NALDRNDVN- WADDALSNLE GVQCCRPPAP WNNVEQQVVY EDWTATLD-S DYTWAFCRVG YFLQGLYRSD TGWPRFKGYL FNLESARCTK PANHPLNYGT CQDIDVSSCM GRKGQCS-CP 160  xgBioGlab47.1-B1
BGLB000137            1   ---MFLQIFL AVTLVQYVSS Q-CTYSSWWY S---FDTPGQ SKCNDINSYI NALDRNDVN- WADDALSNLE GVQCCRPPAP WNNVEQQVVY EDWTATLD-S DYTWAFCRVG YFLQGLYRSD TGWPRFKGYL FNLESARCTK PANHPLNYGT CQDIDVSSCM GRKGQCS-CP 160  BGLB000137
BB02-B1               1   ---MFLQIFV AVTLVQYVSS Q-CTYSSWWY S---FDTPGQ SKCNEINSYI NALDRNDVN- WADDALSNLE GVQCCRPPAP WNNVEQQVVY EDWTATLD-S DYTWAFCRVG YFLQGLYRSD TGWPRFKGYL FNLESARCTK PANHPLNYGT CQDIDVSSCM GRKGQCS-CP 160  BB02-B1
xgBioGlab47.1-B2      1   ---MLVKFLF AATLVQYVSS Q-CTYSSWWY S---FDTPGQ SKCNEINSYI NALDRNDVN- WADDALSNLE GVQCCRPPAP WNNVEQQVVY EDWTATLD-S DYTWAFCRVG YFLQGLYRSD TGWPRFKGYL FNLESARCTK PANHPLNYGT CQDIDVSSCM GRKGQCS-CP 160  xgBioGlab47.1-B2
BGLB000033            1   ---MLVQFLF AATLLQYVSS Q-CTYSSWWY S---FDTPGQ SKCNEINSYI NALDRNDVN- WADDALSNLE GVQCCRPPAP WNNVEQQVVY EDWTATLD-S DYTWAFCRVG YFLQGLYRSD TGWPRFKGYL FNLESARCTK PANHPLNYGN CQDIDVSSCM GRKGQCS-CP 160  BGLB000033
BB02-B2               1   ---MLVQFLF AATLVQYVSS Q-CTYSSWWY S---FDTPGQ SKCNEINSYI NALDRNDVN- WADDPLSNLE GVQCCQPPAP WNNVEQQVVY EDWTATLD-S DYTWAFCRVG YFLQGLYRSD TGWPRFKGYL FNLESARCTK PANHPLNYGT CQDIDVSSCM GRKGQCS-CP 160  BB02-B2
xgBioGlab47.1-B3      1   ---MILQIFV AVSLMQYVRP Q-CTYSNWGT S---FDKKGQ SKCNEINAYI NGLDRNVVSN WGDDPLTFLE GAVCCQPPSP SNNVEQQVVY DDWTPALD-K DYSWAFCRVG YFLQGLYRSD TGWPHFKGYL FNLEHARCTK PANLPLNYGT CQDVDISSCM SRQGKCS-CP 161  xgBioGlab47.1-B3
BGLB000003            1   ---MILHIFV AVSLMQYVRP Q-CTYSNWGT S---FDKKGQ SKCNEINAYI NGLDRNVVSN WGDDPLTFLE GAVCCQPPSP WNNVEQQVVY DDWTSALD-K DYSWAFCRVG YFLQGLYRSD TGWPNYKGYL FNLEHARCTK PANLPLNYGT CQDVDISSCM SRQGKCS-CP 161  BGLB000003
BB02-B3               1   ---MILHIFV AVSLMQYVRP Q-CTYSNWGT S---FDKKGQ SKCNEINAYI NGLDRNVVSN WGDDPLTFLE GAVCCQPPSP SNNVEQQVVY DDWTPALD-K DYSWAFCRVG YFLQGLYRSD TGWPHFKGYL FNLEHARCTK PANLPLNYGT CQDVDISSCM SRQGKCS-CP 161  BB02-B3
xgBioGlab47.1-B4      1   ---MLYQIFV VVSLMPYVRS Q-CSYSDWSL S---FDKTGQ SMCDQINSYI NALDRNHVD- WDDDPISHLE GVKCCQPTSP WNNVEQQVVY EDWTTTLD-R DNEWAFCPVG YFLQGLYRSD TGWPRYKGYL FNLDSARCTK PANHPLNYGE CSDIDITNCI SEKRQCS-CP 160  xgBioGlab47.1-B4
BGLB000104            1   ---MLYQIVV VVSLMSYVRS Q-CSYSDWSL S---FDKPGQ SMCDQINSYI NALDRNHVD- WDDDPISHLE GVQCCQPTSP WNNVEQQVVY EDWTTTLD-R DNEWAFCPVG YFLQGLYRSD TGWPRYKGYL FNLDSARCTK PANHPLNYGE CSDIDITNCI SEKGQCS-CP 160  BGLB000104
BB02-B4               1   ---MLYQIVV VVSLMSYVRS Q-CSYSDWSL S---FDKPGQ SMCDQINSYI NALDRNHVD- WDDDPISHLE GVQCCQPTSP WNNVEQQVVY EDWTTTLD-R DNEWAFCPVG YFLQGLYRSD TGWPRYKGYL FNLDSARCTK PANHPLNYGE CSDIDITNCI SEKGQCS-CP 160  BB02-B4
BGLB000002            1   ---MLLQIYG AVTLIHYVTS Q-CAYTSWRY S---FDNPGQ SKCNQINYYI NALDRFDVD- WNDDTLANLD GVQCCLPPSP WNNVEQQVVY EDWTYYLD-N AFSWAFCPVG YFIQGLYRSR NGAPSYKGYI YNIESARCTK PASHPLDYGN CKEIDTSICI SRRGQCS-CP 160  BGLB000002
BB02-B5               1   ---MLLQIYG AVTLIHYVTS Q-CAYTSWRY S---FDNPGQ SKCNQINYYI NALDRFDVD- WNDDTLANLD GVQCCLPPSP WNNVEQQVVY EDWTYYLD-N AFSWAFCPVG YFIQGLYRSR NGAPSYKGYI YNIESARCTK PASHPLDYGN CKEIDTSICI SRRGQCS-CP 160  BB02-B5
xgBioGlab47.1-B6      1   ---MLYQIFV VVSLMPYVRS Q-CSY-NWLL S---FDKPGQ SKCDQINYYI NALFRDYVN- RNYDPISLLE GVQCCQPNSP WYSVEQQVVF EDWTTILD-R KNEWAFCRVG YFLQGLYISS S------GYI SAIEGARCTK PANHPLNYGD CSDVDIANCM AQVGLCS-CP 153  xgBioGlab47.1-B6
BGLB000004            1   ---MLYQIFV VVSLMPYVRS Q-CSYSNWLL S---FDKPGQ SKCDQINYYI NALFRDYVN- RNYDPISLLE GVQCCQPNSP WYSVEQQVVF EDWTTILD-R TNEWAFCRVG YFLQGLYRSS S------GYI SAIEGARCTK PANHPLNYGD CTDLDIANCM AQVGLCS-CP 154  BGLB000004
BB02-B6               1   ---MLYQIFV VVSLMPYVRS Q-CSYSNWLL S---FDKPGQ SKCDQINYYI NALFRDYVN- RNYDPISLLE GVQCCQPNSP WYSVEQQVVF EDWTTILD-R KNEWAFCRVG YFLQGLYISS S------GYI SAIEGARCTK PANHPLNYGD CSDVDIANCM AQVGLCS-CP 154  BB02-B6
xgBioGlab47.1-B7      1   ---MLLQILV AVSVMHYVQS Q-CSFTSWQY S---FNDLGQ SKCNEINDYI HALYRHDKY- SDDDDLSLLD GAQCCPLGDP WRNVDQQVVY EDWTYDLD-Y DYSWAFCRVG YFLQGLYRSR DIWSPYKGYL NHLEAARCTK PANHPLDYGS CIDVDTSACM SQKGFCY-CP 160  xgBioGlab47.1-B7
BGLB000119            1   ---MLLQILV AVSVMHYVQS Q-CSFTSWQY S---FNDLGQ SKCNEINDYI HALYRHDKY- SDDDDLSLLD GAQCCPLGDP WRNVDQQVVY EDWTYDLD-Y DYSWAFCRVG YFLQGLYRSR DIWSPYKGYL NHLEAARCTK PANHPLDYGS CIDVDTSACM SQKGFCY-CP 160  BGLB000119
BB02-B7               1   ---MLLQILV AVSVMHYVQS Q-CSFTSWQY S---FNDPGQ SKCNEINDYI HALYRHDKY- SDDDNLSLLD GAQCCQLGDP WRNVDQQVVY EDWTYNLD-Y DNSWAFCHVG YFLQGLYRSR DIWSPYKGYL NHLEAARCTK PANHPLDYGS CMDVDTTACM SQKGFCY-CP 160  BB02-B7
xgBioGlab47.1-B8      1   --MVLVHAVV IVTVMSLAKS QICTNTSWAI S---FDHRGQ STCRELDKYI NGFLRGNLR- GRNDPITNLE QVQCCSASEP WPKVERQVVY ADWTYQLD-D NFRWAFCPVG YFLHGLYRSS ASSP---GYL NTIESARCAK PASHPITYGS CQNVDTSSCM SRQGTCS-CP 159  xgBioGlab47.1-B8
BGLB000016            1   --MVLVHAVV IVTVLSLAKS QICTNTSWAI S---FDHLGQ STCNELDKYI NGFVRGHLR- GGDDPITNLE QVQCCSASEP WPNVERQVVY ADWTYQLD-D NFRWAFCPVG YFLHGLYRSS ASSP---GYL NTIEGARCAK PASHPITYGS CQNVDTSSCM SRQGTCS-CP 159  BGLB000016
BB02-B8               1   --MVLVHAVV IVTVMSLAKS QICTNTSWAI S---FDHRGQ STCRELDKYI NGFVRGHLR- GGDDPITNLE QVQCCSASQP WPKVERQVVY ADWTYQLD-D NFRWAFCPVG YFLHGLYRSS ASSP---GYL NTIESARCAK PASHPITYGS CQNVDTSSCM SRQGTCS-CP 159  BB02-B8
xgBioGlab47.1-B9      1   -MLKALLLAV LMGLKSLGVN AACYREDWSD S---FEREGL STC---QHYI RKFQVNGDV- -ANGRLGLLE GAECCSAPPP WISSKLQTVH ANWWDSFN-S -RGAVACPEG FFLRGLKRTG GSP----GYL YNLEEGRCTK PADHPRHYES CYTEDISLCI RRNGLCG-CR 154  xgBioGlab47.1-B9
BGLB000044            1   -MLKALLLAL LMGLKSLGVN AVCYREDWSD S---FEREGL STC---QHYI RKFQVNGDV- -ANGRLGLLE GAECCSAPPP WISSKLQTVH ANWWDSFN-S -RGAVACPEG FFLRGLKRTG GSP----GYL YNLEEGRCTK PADHPRHYKS CYTEDISLCI RRNGLCG-CR 154  BGLB000044
BB02-B9               1   -MLKALLLAL LMGLKSLGVN AACYREDWSD S---FEREGL STC---QHYI RKFQVNGDV- -ANGRLGLLE GAECCSAPPP WISSKLQTVH ANWWDSFN-S -RGAVACPEG FFLRGLKRTG GSP----GYL YNLEEGRCTK PADHPRHYKS CYTEDISLCI RRNGLCG-CR 154  BB02-B9
xgBioGlab47.1-B10     1   ---------- ---------- ---------- ---------- -MCDEVDYYI RGFERSSAN- -GDHSLNRLE GVECCSAPTP WSDMELLVVY QDWTYDLD-T GNHWVFCPNG YFLQGLQRSQ GR------NL DNIEAARCTK PAIHPHRHSD CYDEDISSCF DGTGLCS-CR 119  xgBioGlab47.1-B10
BGLB000056            1   -MVLQLIVFF VLVKTT---H TACTKSSWLT S---FDSAGQ SMCDEVDYYI RGFERSSAN- -GDHSLNRLE GVECCSAPTP WSDMELLVVY QDWTYDLD-T GNHWVFCPNG YFLQGLQRSQ GR------NL DNIEAARCTK PAIHPHRHSD CYDEDISSCF DGTGLCS-CR 153  BGLB000056
BB02-B10              1   -MVLQLIVFF VLVKTT---H TACTKSSWLT S---FDSAGQ SMCDEVDYYI RGFERSSAN- -GDHSLNRLE GVECCSAPTP WSDMELLVVY QDWTYDLD-T GNHWVFCPNG YFLQGLQRSQ GR------NL DNIEAARCTK PAIHPHRHSD CYDEDISSCF DGTGLCS-CR 153  BB02-B10
xgBioGlab47.1-B11     1   ---MMFVKVW VAMTLLGVAD AVCTYTSWML S---FDSPGQ SKCNELNHYI YGLERSDVH- -KDDQLFFLE GVHCCSAPPQ WSKIEVQVVY ADWTKTLD-R PNQWAHCPAG FFLQGLQRSK SGK--NKGYV YNIENGRCAK PANHPFYYGD CYSHYID--F SRSGLYS-CN 156  xgBioGlab47.1-B11
BGLB000108            1   ---------- ---------- ---------- ---------- ---------- ---------- ---------- ---------- ---------- ---------- ---------- ---------- ---------- ---------- ---------- ---------- ----------   1  BGLB000108
BB02-B11              1   ---MMFVKVW VAMTLLGVAD AVCTYTSWML S---FDSPGQ SKCNELNHYI YGLERSDVH- -KDDQLFFLE GVQCCSAQPQ WSNIEVQVVY ADWTKTLD-R QNQWAHCPAG FFLQGLQRSK SGK--NKGYV YNIENGRCAK PANHPFYYGD CYSHYID--F SRSGLYS-CN 156  BB02-B11
xgBioGlab47.1-B12     1   ---MFVLKFG VLLALIGKGS ALCAHTSWWD S---FNSPGQ SKCNEANFYI NGLSRNEYK- -KDDQLTYLE GVQCCSAPSQ WSNGELQVVY ADWVPTLYYK YNAWANCPTG FFLQGLLRTD SGWPWYAGYL YHIETARCSK PAEHPFYYGD CYSQDIN--F NENGLFS-CN 159  xgBioGlab47.1-B12
BGLB021232-RA         1   ---MFVLKFG VLLALIGKGS ALCAHTSWWD S---FNSPGQ SKCNEANFYI NGLSRNEYK- -KDDQLTYLE GVQCCSAPSQ WSNGELQVVY ADWVPTLYYK YNTWANCPTG FFLQGLLRTD SGWPWYAGYL YHIETARCTK PAEHPFYYGD CYSQDIN--F NENGLFS-CN 159  BGLB021232-RA
BB02-B12              1   ---MFVLKFG VLLALIGKGS ALCAHTSWWD S---FNSPGQ SKCNEANFYI NGLSRNEYK- -KDDQLTYLE GVQCCSAPSQ WSNGELQVVY ADWVPTLYYK YNAWANCPTG FFLQGLLRTD SGWPWYAGYL YHIETARCTK PAEHPFYYGD CYSQDIN--F NENGLFS-CN 159  BB02-B12
xgBioGlab47.1-B13     1   ---MLLLNVC VSLTLIGLAN ALCAETSWWH S---FDSAGQ SKCDEANYYI KGLKRNDLL- -NDDQIFLLE GVLCCSAPSQ WSNSELQVVY ADWTRKWD-R SEEWAYCPQG FFLQGMQRST TGWPWYAGYL QNIENGRCAK PANHPFYYGH CYVEIIE--F NSNGMFS-CQ 158  xgBioGlab47.1-B13
BGLB000122            1   ---MLLLNVC VSLTLIGLAN ALCAETSWWH S---FDSAGQ SKCDEANYYI KGLKRNDLL- -NDDQIFLLE GVLCCSAPSQ WSNSELQVVY ADWTRKWD-R SEEWAYCPQG FFLQGMQRST TGWPWYAGYL QNIENGRCAK PANHPFYYGH CYVKIIE--F NSNGMFS-CQ 158  BGLB000122
BB02-B13              1   ---MLLLNVC VSLTLIGLAN ALCAETSWWH S---FDSAGQ SKCDEANYYI KGLKRNDLL- -NDDQIFLLE GVLCCSTPSQ WSNSELQVVY ADWTRKWD-R SEEWAYCPQG FFLQGMQRST TGWPWYAGYL QNIENGRCAK PANHPFYYGH CYVEIIE--F NSNGMFS-CQ 158  BB02-B13
BGLB000086            1   ---------- ---------- ---------- ---------- ---------- ---------- ---------- ---------- ---------- ---------- ---------- ---------- ---------- ---------- ---------- ---------- ----------   1  BGLB000086
BB02-B14              1   ---MLLLSLC VCVTLIGLTN AVCAETSWWH S---FDSSGQ SKCDEANYYI KGLKRNDLL- -NDDQIFLLE GVLCCSAPSQ WSNIELQVVY ADWTHKWD-R SEEWAYCPQG FFLQGMQRST TGWPWYAGYL QNIENGRCAK PANHPFYYGH CYIKDID--F SSRGMFS-CP 158  BB02-B14
xgBioGlab47.1-B15     1   --MKLFLFIC VSVTLLKTAN PLCTYTSWLH S---FDSTGL SVCKESKYYI HGFDRNTYM- -DDDKLYLLE GVQCCSAPHQ WSNSEIQVVY ADWTYKLD-R FDDWVNCPAG FFLHGLYRSL SIS---EGYL GQIEDARCAK PANHPFYYGD CYVYNIN--F ITKGLYS-CK 156  xgBioGlab47.1-B15
BGLB000076            1   --MKLFLFIC VSVTLIKTAN PLCTYTSWLH S---FDSTGL SVCKESQYYI HGFDRNTYM- -DDDKLYLLE GVQCCSAPHQ WSNSEIQVVY DDWTYKLD-R FDVWVNCPAG FFLHGLYRSL SIS---EGYL GQIEDARCAK PANHPFYYGD CYVYNIN--F IRKGLYS-CK 156  BGLB000076
BB02-B15              1   --MKLFLFIC VSVTLIKTAN PLCTYTSWLH S---FDSTGL SVCKESQYYI HGFDRNTYM- -DDDKLYLLE GVQCCSAPHQ WSNSEIQVVY DDWTYKLD-R FDVWVNCPAG FFLHGLYRSL SIS---EGYL GQIEDARCAK PANHPFYYGD CYVYNIN--F IRKGLYS-CK 156  BB02-B15
xgBioGlab47.1-B16     1   --MRLFLFFC VSVTLIKTAN LLCTNSSWVH S---FDSAGQ SVCRESKYYI NGFDRNTYM- -DDDKLYLLE GVQCCSAPHQ WSDSEIQVNY ADWTNIFD-R FDEWAYCPAG FFLHGLYRSL SIS---EGYL GHIEDARCTK PANHPFYYGE CYVQNIN--F ITKGLYS-CK 156  xgBioGlab47.1-B16
BGLB000125-RA         1   --MRLFLFFC VSVTLIKTAN PLCTNTSWWH S---FDSAGQ SACKESKYYI HGFDRNTYM- -DDDKLYLLE GVQCCSAPHQ WSNSEIQVVY DDWTYKLD-R FDVWVNCPAG FFLHGLYRSL SIS---EGYL EQIEYARCTK PANHPFYYGE CYVQNIN--F ITKGLYS-CN 156  BGLB000125-RA
BB02-B16              1   --MRLFLFFC VSVTLIKTAN PLCTNTSWWH S---FDSAGQ SACKESKYYI HGFDRNTYM- -DDDKLYLLE GVQCCSAPHQ WSNSEIQVVY DDWTYKLD-R FDVWVNCPAG FFLHGLYRSL SIS---EGYL EQIEYARCTK PANHPFYYGE CYVQNIN--F ITKGLYS-CN 156  BB02-B16
xgBioGlab47.1-B17     1   ---MHFLKAC AIMTSIEITN AYCTYESWGT S---FDSPGQ STCQEVNYYI QGLDRNDLK- -IDDQINLLE GVKCCSAPSQ WSNNEEQVVY GDWTVTFD-H NDIWANCPEG FFLQGLQRSS TGSPNYAGYL HNIEYARCTK PANHPFYYEK CYVKEIN--F NYKGIFS-CN 158  xgBioGlab47.1-B17
BGLB000064            1   ---MHFLKAF VIMTLIVITN AYCTYESWGT S---FDSPGQ STCQEVNYYI QGLDRNDLK- -IDDQINLLE GVKCCSAPSQ WSNNEEQVVY GDWTVTFD-H NDIWANCPEG FFLQGLQRSS TGSPNYAGYL HNIEYARCTK PANHPFYYEK CYVKEIN--F NYKGIFS-CN 158  BGLB000064
BB02-B17              1   ---MHFLKAC AIMTLIEITN AYCTYESWGT S---FDSPGQ STCQEVNYYI QGLDRNDLK- -IDDQINLLE GVKCCSAPSQ WSNNEEQVVY ADWTVTFD-H NDIWANCPEG FFLQGLQRSS TGSPNYAGYL HNIEYARCTK PANHPFYYEK CYVKEIN--F NYKGIFS-CN 158  BB02-B17
xgBioGlab47.1-B18     1   ---MFFFKVC ITMTLLEIAN AVCRSTSWTR S---FDSAGQ SKCDRVDFFM RGLQRSDQL- -WYDEISQLD GVQCCSASPK WSKSEPQVIY ADWSDLWD-G YNVWVNCPAG YFLQGLQRSS SGWPWYSGYL HHIDYGRCAK PSHHPFYHGD CYDKNID--F SYSGIFT-CN 158  xgBioGlab47.1-B18
BGLB000010            1   ---MMFFKVC ITMTLFEIAN AVCRSTSWTR S---FDSAGQ SKCDSVDFFM RGLQRSDQL- -WYDEISQLD GVQCCSASPK WSKSEPQVIY ADWSDLWD-G YNVWVDCPAG YFLQGLQRSS SGWPWYSGYL HHIDYGRCAK PSHHPFYHGD CYAKNID--F SYSGIFN-CN 158  BGLB000010
BB02-B18              1   ---MMFFKVC ITMTLFEIAN AVCRSTSWTR R---FDSAGQ SKCDSVDFFM RGLQRSDQL- -WYDEISQLD GVQCCSASPK WSKSEPQVIY ADWSDLWD-G YNVWVDCPAG YFLQGLQRSS SGWPWYSGYL HHIDYGRCAK PSHHPFYHGD CYAKNID--F SYSGIFT-CN 158  BB02-B18
BGLB000001-RA         1   --MMLFSKLW VALTILGIVD ALCTESLWSN R---INYHGQ STCKEPTYYM NGLQRDHLQ- -QNDGLHLLK GAVCCSAPSR FLQSDNLVLY KDWTYVLEEK NDIWANCPEG YFLHGLQRSQ NEDG--VNYL HNIEYARCVK PSNHPHYYGD CYAQNIT--F NKQGVFS-CK 158  BGLB000001-RA
BB02-B19              1   --MMLFSKLW VALTILGIVD ALCTESLWSN R---INYHGQ STCKEPTYYM NGLQRDHLQ- -QNDGLHLLK GAVCCSAPSR FLQSDNLVLY KDWTYVLEEK NDIWANCPEG YFLHGLQRSQ NEDG--VNYL HNIEYARCVK PSNHPHYYGD CYAQNIT--F NKQGVFS-CK 158  BB02-B19
xgBioGlab47.1-B20     1   ---MLFSKLC VTVTLLGIVD ALCTVSWWSQ RTRTVNFNGL SSCNEYNYFM NGLHRDHLE- -QNDGLHLIK GVVCCKATSR FSDSEVLAHH EDWSIIFQES FDIWAFCPDG YFLSGFSRYT DGDA--INYL HNIEYARCIK PSNHPDYHGK CYTQYIN--F NTQGVFS-CK 160  xgBioGlab47.1-B20
BGLB000202            1   ---------- ---------- ---------- ---------- ---------M NGLHRDHLE- -QNDGLHLIK GVVCCKATSR FSDSEVLAHH EDWSIIFQES FDIWAFCPDG YFLSGFSRYT DGDA--INYL HNIEYARCIK PSNHPDYHGK CYTQYIN--F NTQGVFS-CK 114  BGLB000202
BB02-B20              1   ---MLFSKLC VTVTLLGIVD ALCTVSWWSQ RTRTVNFNGL SSCNEYNYFM NGLHRDHLE- -QNDGLHLIK GVVCCKATSR FSDSEVLAHH EDWSMIFQES FDIWAFCPDG YFLSGFSRYT DGDA--INYL HNIEYARCIK PSNHPDYHGK CYTQYIN--F NTQGVFS-CK 160  BB02-B20
xgBioGlab47.1-B21     1   --MMLFSKLY VTMTLLGIVD GECVKTSWVQ N---INYEGQ LKCKEYDFYI NGLERNSTE- -NFDGLHLLT GVHCCSPPLR YLDSDTIVIY ENWAWKFEQ- NNRWAYCPQG FFLHGLQTTS SDSG--INYL QNIKYARCIK PSNHPESYAD CYYQNLN--F NKQGVFN-CR 157  xgBioGlab47.1-B21
BGLB000109            1   --MMLFSKLC VTMTLLGIVD GECVKNSWVQ N---INYEGQ LKCKEYDFYI NGLERNSTD- -NFDGLHLLT GVHCCSPPLR YLDSDTIVIY ENWAWKFEQ- NNRWAYCPQG FFLHGLQTTS SDSG--INYL QNIKYARCIK PSNHPESYAD CYYQNLN--F NKQGVFN-CR 157  BGLB000109
BB02-B21              1   --MMLFSKLC VTMILLGIVD GECVKTSWVQ N---VNYEGQ LKCKEYDFYI NGLERNSTE- -NFDGLHLLT GVHCCSPPLR YLDSDTIVIY ENWGWKFEQ- NNRWAYCPQG FFLHGLQTTS SDSG--INFL QNIKYARCIK PSNHPESYAD CYFQNLN--F NKQGVFN-CR 157  BB02-B21
xgBioGlab47.1-B22     1   ---MLFLKFC FIMTLLEIVD GKCHNFPWSG NFKVAGQSIC HRGSIGNSFI KGLIRNSLKS -KSEGLDVIA AVKCCTFDEE YLSGDVVVVY ANWQKTFSK- DQSWSRCPSG YFLQGIYTTV NSDG--TTQL HNIEHGRCAK IGIYPPYYRD CYAEDVH--F NEAKLYECTK 161  xgBioGlab47.1-B22
BGLB028593-RA         1   ---MLFLKFC FIMTLLEIVD GKCHNFPWSG NFKVAGQSIC HRGSIGNSFI KGLIRNSLKS -KSEGLDVIA AVKCCTFDEE YLSGDVVVVY ANWQKTFSK- DQSWSRCPSG YFLQGIYTTV NSDG--TTHL HNIEHGRCAK IGIYPPYYRD CYAEDVH--F NEAKLYECTK 161  BGLB028593-RA
BB02-B22              1   ---MLFLKFC FIMTLLEIVD GKCHNFPWSG NFKVAGQSIC HRGSIGNSFI KGLIRNSLKS -KSEGLDVIA AVKCCTFDEE YLSGDVVVVY ANWQKTFSK- DQSWSRCPSG YFLQGIYTTV NSDG--TTHL HNIEHGRCAK IGIYPPYYRD CYAEDVH--F NEAKLYECTK 161  BB02-B22
xgBioGlab47.1-B23     1   MTNMLLSKLR VFMALLEVVN VNGMTVPWSK SNRSDHQVLC KD---YDSYV VGFGLN---- -HTDALTNLE SAKCEKMQRG NSS-AIVVEY VDWNKKIDK- NQVWLHCPSG FFLQGMQIFL IKP----TRM LN---GRCCK PAKHPFYYRD CNIVEVT--D HQFNFLLSCP 151  xgBioGlab47.1-B23
BGLB021098-RA         1   MTNMLLSKLR VFMTLLGVVN VNGMTVPWSK SNRSDHQVLC KD---YDSYV VGFGLN---- -HTDALTNLE SAKCEKMQRG NSS-AIVVEY VDWNKKIDK- NQVWLHCPSG FFLQGMQIFL IKP----TRM LN---GRCCK PAKHPFYYRD CNIVEVT--D HQFNFLLSCP 151  BGLB021098-RA
BB02-B23              1   MTNMLLSKLR VFMTLLGVVN VNGMTVPWSK SNRSDHQVLC KD---YDSYV VGFGLN---- -HTDALTNLE SAKCEKMQRG NSS-AIVVEY VDWNKKIDK- NQVWLHCPSG FFLQGMQIFL IKP----TRM LN---GRCCK PAKHPFYYRD CNIVEVT--D HQFNFLLSCP 151  BB02-B23

                                   180        190        200        210        220        230        240        250        260        270        280        290        300        310        320        330        340
B1-Antibody_PEP      15   ---------- ---------- ---------- ---------- ---------- ---------- ---------- ---------- ---------- ---------- ---------- ---------- ---------- ---------- ---------- ---------- ----------  15  B1-Antibody_PEP
xgBioGlab47.1-B1    161   GGYFLTGLYR ADGDDLYFLK KIRCCTPAAK PLEMDEKSKI QTRIMDTTLW NMATLAHYMG YGWCYGCHGL AVGEDFTRNG FTWAADTRTF WG-KWCEGDK NGERLNLVFG DWGFAVKEII YGKSVIEDLQ AESVDSGVLY NRASSPVTES IERSKTIQET ITHSTTSTFT 329  xgBioGlab47.1-B1
BGLB000137          161   GGYFLTGLYR ADGDDLYFLK KIRCCTPAAK PLEMDEKSKI QTRIMDTTLW NMATLAHYMG YGWCYGCHGL AVGEDFTRNG FTWAADTRTF WG-KWCEGDK NGERLNLVFG DWGFAVKEII YGKSVIEDLQ AESVDSGVLY NRASSPVTES IERSKTIQET ITHSTTSTFT 329  BGLB000137
BB02-B1             161   GGYFLTGLYR ADGDDLYFLK KIRCCTPAAK PLEMDEKSKI QTRIMDTTLW NMATLAHYMG YGWCYGCHGL AVGEDFTRNG FTWAADTRTF WG-KWCEGDK NGERLNLVFG DWGFAVKEII YGKSVIEDLQ AESVDSGVLY NRASSPVTES IERSKTIQET ITHSTTSTFT 329  BB02-B1
xgBioGlab47.1-B2    161   GGYFLTGLYR ADGDDLYFLK KIRCCTPAAK PLEMDEKSKI QTRIMDTTLW NMATLAHYMG YGWCYGCHGL AVGEDFTRNG FTWAADTRTF WG-KWCEGDK NGERLNLVFG DWGFAVKEII YGKSVIEDLQ AESVDSGVLY NRASSPVTES IDRTKTIQET ITHSTTSTFT 329  xgBioGlab47.1-B2
BGLB000033          161   GGYFLTGLYR ADGDDLYFLK KIRCCTPAAK PLEMDEKSKI QTRIMDTTLW NMATLAHYLG YGWCYGCRGV AVGEDFTRNG FTWAADTRSF WG-KWCEGDK NGERLNLVFG DWGFAVKEII YGKSVIEDLQ AESVDSGVLY NRASSPVTES IDRTKTIEET VTHSTTSTFT 329  BGLB000033
BB02-B2             161   GGYFLTGLYR ADGDDLYFLK KIRCCTPAAK PLEMDEKSKI QTRIMDTTLW NMATLAHYMG YGWCYGCHGL AVGEDFTRNG FTWAADTRSF WG-KWCEGDK NGERLNLVFG DWGFAVKEII YGKSVIEDLQ AESVDSGVLY NRASSPVTES IDRTKTIQET ITHSTTSTFT 329  BB02-B2
xgBioGlab47.1-B3    162   GGYFMTGLYR ADGDDLYFLK KIRCCTLPAK ELVMDEKSKI QTRIMDTTLW NMATLAHYLG YGWCYGCHGM AVGEDFNRNG DTWAADTRSF WG-TSCDGDK SGERLNLVFG DWGFAVKEII YGKSVIEDLQ AESVDSGVVY NRASSPVTES IERTKTIQET ITHSTTSTFT 330  xgBioGlab47.1-B3
BGLB000003          162   DGYFMTGLYR ADGDDLYFLK KIRCCTLPAK ELVMDEKSKI QTRIMDTTLW NMATLAHYLG YGWCYGCHGM AVGEDFNRNG DTWAADTRSF WG-TSCDGDK SGERLNLVFG DWGFAVKEII YGKSVIEDLQ AESVDSGVVY NRASSPVTES IERTKTIQET ITHSTTSTFT 330  BGLB000003
BB02-B3             162   GGYFMTGLYR ADGDDLYFLK KIRCCTLPAK ELVMDEKSKI QTRIMDTTLW NMATLAHYLG YGWCYGCHGM AVGEDFNRNG DTWAADTRSF WG-TSCDGDK SGERLNLVFG DWGFAVKEII YGKSVIEDLQ AESVDSGVVY NRASSPVTES IERTKTIQET ITHSTTSTFT 330  BB02-B3
xgBioGlab47.1-B4    161   GGYFLTGLYR ADGDDLYFLK KIRCCSPAAK PLEMDEKSKI ETRIMDTTLW NMAYLAHYLG YGWCYGCRGV DVGEDFTRIG DSWEAAKTPF KG-KSCEGDK SEERLNLVFG DWGFAVKKII YGESVIEKLQ PETIDSGELN NRASKPVTES ISRSKTIVES ITHTTTSSFT 329  xgBioGlab47.1-B4
BGLB000104          161   GGYFLTGLYR ADGDDLYFLK KIRCCSPAAK PLEMDEKSKI ETRIMDTTLW NMANLAHYLG YGWCYGCRGV DVGEDFTRIG DSWEAAKTPF KG-KSCEGDK SEERLNLVFG HWGFAVKKII YGESVIEKLQ PETIDSGELN NRASKPVTES ISRSKTIVES ITHTTTSSFT 329  BGLB000104
BB02-B4             161   GGYFLTGLYR ADGDDLYFLK KIRCCSPAAK PLEMDEKSKI ETRIMDTTLW NMAYLAHYLG YGWCYGCRGV DVGEDFTRIG DSWEAAKTPF KG-KSCEGDK SEERLNLVFG DWGFAVKKII YGESVIEKLQ PETIDSGELN NRASKPVTES ISRSKTIVES ITHTTTSSFT 329  BB02-B4
BGLB000002          161   VGYFLTGIYE ADRGDLYFLK KIKCCTPAAK PLEMDEKSKI QTRIMDITLW NMANLAHYLG YGWCLGCRGV DVGEDFIKNG NTWKAATTPF WD-SRCDGDK NWERLNLVFD NWGFGVKEIV YGKTVIDNLQ PETVDSGVIY NKASSPVTES IERSKTIQDT ITHTTTSTFT 329  BGLB000002
BB02-B5             161   VGYFLTGIYE ADRGDLYFLK KIKCCTPAAK PLEMDEKSKI QTRIMDITLW NMANLAHYLG YGWCLGCRGV DVGEDFIRNG NTWKAATTPF WD-SRCDGDK NWERLNLVFD NWGFGVKEIV YGKTVIDNLQ PETVDSGVIY NKASSPVTES IERSKTIQDT ITHTTTSTFT 329  BB02-B5
xgBioGlab47.1-B6    154   GGYFLTGLYR SDGNNLSYLK KIKCCTPADK PLDLDEKSKI QTHIMDTTLW NMANLAHYLG YGWCLGCRGV DVGEDFIRNG NTWKAATTPF WD-SRCGGDK NWERLNLVFD NWGFGVKEIV YGKTVIENLQ PETVDSGVIY NKASSPVTES IERSKTIQDT ITHTTTSTFT 322  xgBioGlab47.1-B6
BGLB000004          155   GGYFLTGLYR SDGNNLSYLK KIKCCTPADK PLDLDEKSKI QTHIMDTTLW N-GFLSQFLS P--------- ---------- ---------- ---------- ---------- ---------- ---------- ---------- ---------- ------PQHT ITHTTTSTFT 228  BGLB000004
BB02-B6             155   GGYFLTGLYQ SDGYNLSYLK KIKCCTPADK PLDLDEKSKI QTHIMDSTLW NMANLAHYLG YSWCLGCRGV DVGEDFIRNG NTWKAATTPF WD-SRCDGDK NWERLNLVFD NWGFGVKEIV YGKTVIENLQ PETVDSGVIY NKASSPVTES IERSKTIQDT ITHTTTSTFT 323  BB02-B6
xgBioGlab47.1-B7    161   GGHWLTGIYR ADGDDLYFLK KIRCCTPAAK PLDMDEKSRI QTRIMDTTLW NMANLAHFLG YGWCYGCRGL AVGEDFIRNG NTWAAATTLF WD-SRCDGDK NGERLNLFFD NWGFGVKEIV YGQSVIEDLQ PETVDSGVIY NKASSPVTES IERSKTIQDT ITHTTTSTFT 329  xgBioGlab47.1-B7
BGLB000119          161   GEHWLTGIYR ADGDDLYFLK KIRCCTPAAK PLDMDEKSRI QTRIMDTTLW NMANLAHFLG YGWCYGCRGL AVGEDFIRNG NTWAAATTPF WD-SRCDGDK NGERLNLFFD NWGFGVKEIV YGQSVIEDLQ PETVDSGVIY NKASSTVTES IERSKTIQDT ITHTTTSTFT 329  BGLB000119
BB02-B7             161   DGHWLTGIYR ADGDDLYFLK KIRCCTPAAK PLDLDEKSRI QTHIMDTTLW NMANLAHFLG YGWCYGCRGL AVGEDFIRNG NTWAAATTPF WD-SRCDGDK NGERLNLFFD NWGFGVKDIV YGQSVIEDLQ PETVDSGVIY NKAASPVTES IERSKTIQDT ITHTTTSTFT 329  BB02-B7
```

```
xgBioGlab47.1-B8    160 RGFFVTGIYR ADDDDLYNLK KMKCCEPVDK PDEINSLSKI QTRIMDTTLW NLANLAYYLG YEWSYGCRAL NVGEDFYKDG FTWVAETKIF HH-GSCNGYN RYNRLKLVFD HWGLSVKEIK YGPKVTESLQ PESFDSGIIR NTASSSITES IERTRTVQDT ITHIATTSFT 328 xgBioGlab47.1-B8
BGLB000016          160 RGFFVTGIYR ADDDDLYNLK KMKCCEPVDK PDEIDSLSKI QTRIMDTTLW NLANLAYYLG YEWSYGCRAL NVGEDFYKDG FTWVAETKIF HH-GSCDGYN RYNRLKLVFD HWGLSVKEIK YGPKVTESLQ PESFDSGIIR NTASSSITES IERTRTVQDT ITHIATTSFT 328 BGLB000016
BB02-B8             160 RGFFVTGIYR ADDDDLYNLK KMKCCEPVDK PDEINSLSKI QTRIMDTTLW NLANLAYYLG YEWSYGCRAL NVGEDFYKDG FTWVAETKIF HH-GSCDGYN RYNRLKLVFD HWGLSVKEIK YGPKVTESLQ PESFDSGIIR NTASSSITES IERTRTVQDT ITHIATTSFT 328 BB02-B8
xgBioGlab47.1-B9    155 ESYFIVGLER DECQELQCIK KLKCCSMLDQ IEPLTGLGKV KERIMDVTQS NMALLGHILG YGWAGGCREP YVGEGYNRVG DTWEADES-- ---SGCNGYM SDYRLNMVYG NWDFELVNMN KGIQVTTQLE PETIDSGTSI NNDSTPLTVQ LSKMFSITRQ VTHTVTDTWE 319 xgBioGlab47.1-B9
BGLB000044          155 ESYFIVGLER DECQELQCIK KLKCCSMLDQ IEPLTGLGKV KERIMDVTQS NMALLGHILG YGWAGGCREP YVGEGYNRVG DTWEADES-- ---SGCNGYM SDYRLNMVYG NWDFELVNMN KGIQVTTQLE PETIDSGTSI NNDSTPLTVQ LSKMFTVTRQ VTHTVTDTWE 319 BGLB000044
BB02-B9             155 ESYFIVGLER DECQELQCIK KLKCCSMLDQ IEPLTGLGKV KERIMDVTQS NMALLGHILG YGWAGGCREP YVGEGYNRVG DTWEADES-- ---SGCNGYM SDYRLNMVYG NWDFELVNMN KGIQVTTQLE PETIDSGTSI NNDSTPLTVQ LSKMFSITRQ VTHTVTDTWE 319 BB02-B9
xgBioGlab47.1-B10   120 ENYFVTGLYR SKCNSLQCLD KLECCKMADK LEELDGVSKV KTRIMDATLS NMANLAHLMG YGWCSGCMSN YVGEDFYRDG ETWKANQN-- ---GTCNGYK SDQRLSLVFS NWKFTIKNIK YGESVTDEIQ PSTLDKGMTR NNDSETATDT YEISETIMET ITHSSTSSWT 284 xgBioGlab47.1-B10
BGLB000056          154 ENYFVTGLYR SKCNSLQCLD KLECCKMADK LEELDGVSKV KTRIMDATLS NMANLAHLMG YGWCSGCMSN YVGEDFYRDG ETWKANQN-- ---GTCNGYK SDQRLSLVFS NWKFTIKNIK YGESVTDEIQ PSTLDKGMTR NNDSETATDT YEISETIMET ITHSSTSSWT 318 BGLB000056
BB02-B10            154 ENYFVTGLYR SKCNSLQCLD KLECCKMADK LEELDGVSKV KTRIMDATLS NMANLAHLMG YGWCSGCMSN YVGEDFYRDG ETWKANQN-- ---GTCNGYK SDQRLSLVFS NWKFTIKNIK YGESVTDEIQ PSTLDKGMTR NNDSETATDT YEISETIMET ITHSSTSSWT 318 BB02-B10
xgBioGlab47.1-B11   157 EGYYITGLYK ADCDYLHCID RLYCCKMADT PQVVDDPETL KTRIMDTTLW NLANLAAGLG YGWCSGTKGM NVGEDFYRSG DSWVADSRLF WPNKKCDGYK CKERLAIDYS GWTFVVKEIR YGESVIDELI PVSIDGGMRV NEGDSTVNAT FERSQEVTET IVHSSTNSWK 326 xgBioGlab47.1-B11
BGLB000108          1   ---------- ---------- ------MADT PQVVDDPETL KTRIMDTTLW NLAYLAAGLG YGWCSGTKGM NVGEDFYRSG DSWVADSRLF WPNKKCDGYK CKGRLAIDYS GWTFVVKEIR YGESVIDELI PVSIDGGMRV NEGDSTENAT LERSQEVTET IVHSSTNSWK 144 BGLB000108
BB02-B11            157 EGYYITGLYK ADCDYLHCID RLYCCKMADT PQVVDDPETL KTRIMDTTLW NLANLAAGLG YGWCSGTKGM NVGEDFYRSG DSWVADSRLF WPNKKCDGYK CKERLAIDYS GWTFVVKEIR YGESVIDELI PVSIDGGMRV NEGDSTVNAT FERSQEVTET IVHSSTNSWK 326 BB02-B11
xgBioGlab47.1-B12   160 EGYYVKGLYK GNCDYLHCID KLYCCKMADK PEMIDDVDKL KTKIMDTTLT NLANLADKLG YGYCYGTKGV NVGEDFRREH DSWVANTEYY -----CKGDK CDKRLAIDYR NWNLAVKEIK YGQKVIDKLN TENIESGQKE NHLNTSSTVA VERSYAVMDT IEHSMTSSWK 324 xgBioGlab47.1-B12
BGLB021232-RA       160 EGYYVKGLYK GNCDYLHCID KLYCCKMADK PEIIDDVDKL KTKIMDTTLT NLANLADKLG YGYCYGTKGV NVGEDFRREH DSWVANTEYY -----CKGDK CDKRLAIDYR NWNLAVKEIK YGQKVIDKLN TENIESGQKE NHLNTSSTVA VERSYAVMDT IEHSMTSSWK 324 BGLB021232-RA
BB02-B12            160 EGYYVKGLYK GNCDYLHCID KLYCCKMADK PEIIDDVDKL KTKIMDTTLT NLANLADKLG YGYCYGTKGV NVGEDFRREH DSWVANTEYY -----CKGDK CDKRLAIDYR NWNLAVKEIK YGQKVIDKLN TENIESGQKE NHLNTSSTVA VERSYAVMDT IEHSMTSSWK 324 BB02-B12
xgBioGlab47.1-B13   159 DDYYITGLYK SDCGYLHCID KLYCCKMAAG PEVIDNADKV KTKIMETTLF NLANLADKLG YGWCYGTKGV NVGEDFYRSG DSWIADRRLF WPNRWCDGYM CNERLAIDYT GWTLAVKEII YGESVIDELQ PETVHTGVRY NHLNESSSAT FEYSRDVTET VEHSTASSWS 328 xgBioGlab47.1-B13
BGLB000122          159 DDYYITGLYK SDCGYLHCID KLYCCKMATG PEVIDNADKV KTKIMETTLF NLANLADKLG YGWCYGTKGV NVGEDFYRSG DSWIADRRLF WPNRWCDGYM CNERLAIDYT GWTLAVKEII YGESVIDELQ PETVHTGVRY NHLNESSSAT FEYSRDVTET VEHSTTSSWS 328 BGLB000122
BB02-B13            159 DDYYITGLYK SDCGYLHCID KLYCCKMATG PEVIDNADKV KTKIMETTLF NLANLADKLG YGWCYGTKGV NVGEDFYRSG DSWIADRRLF WPNRWCDGYM CNERLAIDYT GWTLAVKEII YGESVIDEY- ------GVRY NHLIESSSAT FEYSRDVTET VEHSTTSSWS 321 BB02-B13
BGLB000086          1   ---------- ---------- ---------- ---------- ----METTLF NLANLAHKLG YGWCFGTKGV NIGDDFYRSG DSWIADRRLF LPNGWCDGYM CNERLAIDYT GWTLAVKKII YGESVIDELQ PEAVHTGVEY NHRNESSSKT FEYSRTVTET IEHSRTSSWS 126 BGLB000086
BB02-B14            159 DDYYITGLYK GNCDKLHCID KLYCCKMAAG PEVIDNADKV KTKIMETTLF NLANLAHKLG YGWCFGTKGV NIGDDFYRSG DSWIADRRLF LPNGWCDGYM CNERLAIDYT GWTLAVKEII YGESVIDELQ PEAVHTGVEY NHRNESSSKT FEYSRTVTET IEHSRTSSWS 328 BB02-B14
xgBioGlab47.1-B15   157 AGYYITGLYK SNCNYLHCLD SLQCCKMADK PMVIDDVHKL KTNIMDTTLW NLANLAHLLG YGWCLGTKGI NVGEDFYRSG DSWVADSRLF WPLTYCEGYK CYERLAIDYI NWNLAVKEIK YGQSVVDELK PDAVHTGVEF NHLNESSSKL FEYSKTVTET IEHSTTSSWK 326 xgBioGlab47.1-B15
BGLB000076          157 AGYYITGLYK SNCNYLHCLD SLQCCKMADK PMVIDDVHKL KTNIMDTTLW NLANLAHLLG YGWCLGTKGI NVGEDFYRSG DSWVADSRLF WPLTYCEGYK CYERLAIDYI NWNLAVKEIK YGQSVVDELK PDAVHTGVEF NHLNESSSKL FEYSKTVTET IEHSTTSSWK 326 BGLB000076
BB02-B15            157 AGYYITGLYK SNCNYLHCLD SLQCCKMADK PMVIDDIHKL KTNIMDTTLW NLANLAHLLG YGWCLGTKGI NVGEDFYRSG DSWVADSRLF WPLIYCEGYK CYERLAIDYI NWNLAVKEIK YGQSVVDELK PDTVHTGVEF NHLDESSSKL FEYSKTVTET IEHSTTSSWK 326 BB02-B15
xgBioGlab47.1-B16   157 EGYYITGLHK GNCKNLHCLD SLQCCKMADK PMVIDDIHKL KTNIMDTTLW NLANLAHLLG YGWCLGTKGS NVGEDFYRSG DSWVADSRLF WPLTKCEGDK CNERLAIDYI DWNLAVKETK YGQSVVDELK PDAVHTGVEF NHLNESSSKL FEYSKTVTET IEHSTTSSWK 326 xgBioGlab47.1-B16
BGLB000125-RA       157 AGYYITGLYK GNCNNLHCLD SLQCCKMADK PMVIDDIHKL KTNIMDTTLW NLANLAHLLG YGWCLGTKGI NVGEDFYRSG DSWVADSRLF WHLTKCEGDK CNERLAIDYI DWNLAVKETK YGQSVVDELK PDAVHTGVEF NHLNESSSKL FEYSKTVTET IEHSTTSSWK 326 BGLB000125-RA
BB02-B16            157 AGYYITGLYK GNCNNLHCLD SLQCCKMADK PMVIDDIHKL KTNIMDTTLW NLANLAHLLG YGWCLGTKGI NVGEDFYRSG DSWVADSRLF WHLTKCEGDK CNERLAIDYI DWNLAVKETK YGQSVVDELK PDAVHTGVEF NHLNESSSKL FEYSKTVTET IEHSTTSSWK 326 BB02-B16
xgBioGlab47.1-B17   159 EDYYVSGLHR GSCDNLYCVD RLQCCKMANV PEEIIDEHKL KTKIMDTTLS NLAYLAAVLG YGWCLGTKGI YVGEDFYREG DSWVADSRLF WPNTRCEGYK CNERLAIDYT GWTLAAKEII YGEHVVDELK PETIDTGVEY NHMNTSSTKL LERSQTVTET IQHSTTSSWK 328 xgBioGlab47.1-B17
BGLB000064          159 EDYYVSGLHR GSCDNLYCVD RLQCCKMANV PEEIIDEHKL KTKIMDTTLS NLAYLAAVLG YGWCLGTKGI YVGEDFYREG DSWVADSRLF WPNTRCEGYK CNERLAIDYT GWTLAVKEII YGEHVVDELK PETIDTGVEY NHLNTSSTKL LERSQTVTET IQHSTTSSWK 328 BGLB000064
BB02-B17            159 EDYYVSGLHR GSCDNLYCVD RLQCCKMANV PEEIIDEHKL KTKIMDTTLS NLAYLAAVLG YGWCLGTKGI YVGEDFYRDG DSWVADSRLF WPNTRCEGYK CNERLAIDYT GWTLAVKEII YGEHVVDELK PETIDTGVEY NHLNTSSTKL LERSQTVTET IQHSTTSSWK 328 BB02-B17
xgBioGlab47.1-B18   159 TDYFITGMYK GDCRYLYCIK ALRCCKMSDT SEIIDDAETF KTKIMDTTLF NLANVADRLG YGWCGGEKGD DVGEDFYKDG DSWIADATPF KPKTECEGFK CDERLKIDYL GWNLAVKNII YGNSIIDSLQ PEVVNTGREV NNMDVSSTRS FEYSTIFTDT VQHSSTSSWK 328 xgBioGlab47.1-B18
BGLB000010          159 TDYFITGMYK GDCRYLYCIK ALRCCKMSDT SEIIDDSETF KTKIMDTTLF NLANLADRLG YGWCGGEKGD DVGEDFYKDG DTWVADARPF KPNTQCEGFK CNERLKIDYL GWNLAVKNIV YGNSIIDSLQ PEVVNTGIEF NYMDVSSTRS FEYSTTLTET IQHSTTSSWK 328 BGLB000010
BB02-B18            159 TDYFITGMYK GDCRYLYCIK ALRCCKMSDT SEIIDDSETF KTKIMDTTLF NLANLADRLG YGWCGGEKGD DVGEDFYKDG DTWVADARPF KPNTQCEGFK CNERLKIDYL GWNLAVKNIV YGNSIIDSLQ PEVVNTGKEF NYMDVSSTRS FEYSTTLTET IQHSTTSSWK 328 BB02-B18
BGLB000001-RA       159 TNFFITGIHK VKGDYLHHID KLYCCAMEEE PEIVYDVDTF KRVIMENTMN NLVRLAMALG YGHCAGNKAI YVGDDFYRDG DSWIADHRLF WGDKKCNGEK CEERLKIDYL DWSFNMKDII YSNGVIDELK PERVYEMGFQ NNQNNNVTKT FEYKNAFSSS ITHSTSISKK 328 BGLB000001-RA
BB02-B19            159 TNFFITGIHK VKGDYLHHID KLYCCAMEEE PEIVYDVDTF KRVIMENTMN NLVRLAMALG YGHCAGNKAI YVGDDFYRDG DSWIADHRLF WGDKKCNGEK CEERLKIDYL DWSFGKKDII YGHSIIDELK PERVYEMGFQ NNQNYNVTKT FEYKNTFSSS TTHSITHSWK 328 BB02-B19
xgBioGlab47.1-B20   161 ENYFITGLHK VKGDYLHHID KLKCCAMQDD PEFVFDVDTF KRIIMDNTLP RLTMLAYAMG YDHISGLKGW MLGDDYFRDG DSWVADTRLY GWDKKCSGIK CEERFKIDYL DWSFHIKDII YGDSVIDELK PEKVYEKLFP NLRNHTVPHI TKLMHEFLPS LKHTITNHWE 330 xgBioGlab47.1-B20
BGLB000202          115 ENYFITGLHK VKSDYLHHID KLKCCAMQDD PEFVFDVDTF KRIIMDNTLP RLTMLAYAMG YDHISGLKGW MLGDDYFRDG DSWVADTRLY GWDKKCSGIK CEERFKIDYL DWSFHIKDII YGDSVIDELK PEKVYEKLFP NLRNHTVPHI TKLKHEFLPS LKHTITNHWE 284 BGLB000202
BB02-B20            161 ENYFITGLHK VKGDYLHHID KLKCCAMQDD PEFVFDVDTF KRIIMDNTLP RLTMLAYAMG YDHISGLKGW MLGDDYFRDG DSWVADTRLY GWDKKCSGIK CEERFKIDYL DWSFHIKDII YGDSVIDELK PEKVYEKLFP NLRNHTVPHI TKLMHEFLPS LKHTITNHWE 330 BB02-B20
xgBioGlab47.1-B21   158 DDYYVTGLHK GNCDSLRCLD KLHCCKMEVD QRILDNVDKV KTKIMESTLP KLVRLAAALG YGHAAGNKGI YIGDDFKREG DSWVADSRLF WGEEECTDLK CKEILAVEYL EWSLTVKEIK YGEKYIDYLE PEVVHSAVFH NENNTSSIQT FQYSNVVSET VTHSPTSRWR 327 xgBioGlab47.1-B21
BGLB000109          158 DDYYVTGLHK GNCDSLRCLD KLHCCKMEVE QRILDNVDKV KTKIMESTLP KLVRLAAALG YGHAAGNKGI YIGDDFKREG DSWVADSRLF WGEEECTDLK CKEILAVEYL EWSLTVKEIK YGEKYIDYLE PEEVHSAVFH NENNTSCIKT FQYSNVVSET VTHSPTSRWR 327 BGLB000109
BB02-B21            158 DDYYVTGLHK GNCDSLRCLD KLHCCKMEVE QRILDNVDKV KTKIMESTLP KLVRLAAALG YGHAAGNKGI YIGDDFKREG DSWVADSRLF WGEEECTDLK CKEILAVEYL EWSLTVKEIK YGEKYIDYLE PEVVHSAVFH NENNTSSIKT FQYSNVVSET VTHSPTSRWR 327 BB02-B21
xgBioGlab47.1-B22   162 DGYFIAGLYR HKCNAPLCIG KFYCCHMEEE -LKIHSAEEL KTKIMEKNLR KLANLATYMG YMNTFGYSGL FVGEDFYVGK DHTTAGRGFL DK-RNCYDIN CNFLFRILYG NWSLAVKDII YGESIHDDLE AEEIHTGEEY NYLDTESTKD IEYSKTIQET ITHTASSTWK 329 xgBioGlab47.1-B22
BGLB028593-RA       162 DGYFIAGLYR HKCNAPLCIG KFYCCHMEEE -LKIHSPEEL KTKIMEKNLR KLANLATYMG YMNTFGYSGL FVGEDFYVGK DHTTAGRGFL DK-RNCYDIN CNFLFRILYG NWSLAVKDII YGESIHDDLE AEEIHTGEEY NYLDTESTKD IEYSKTIQET ITHTASSTWK 329 BGLB028593-RA
BB02-B22            162 DGYFIAGLYR HKCNAPLCIG KFYCCHMEEE -LKIHSPEEL KTKIMEKNLR KLANLATYMG YMNTFGYSGL FVGEDFYVGK DHTTAGRGFL DK-RNCYDIN CNFLFRILYG NWSLAVKDII YGESIHDDLE AEEIHTGEEY NYLDTESTKD IEYSKTIQET ITHTASSTWK 329 BB02-B22
xgBioGlab47.1-B23   152 PNRSVTALHK SRCGKSHCFD KIYCCQLKDE -EEDRSSEEI KQKIMENSLT NLATLANMLG YAYPLGARGI NVGEDFHMEG NSWVANKQDF NG-KECRIPG CNERLTIEYK NWSLAVKDII YGDKVIDNLS PEAIHSGVEY NHMNTSSTKT FEFSKIISET IVHSITSSFT 319 xgBioGlab47.1-B23
BGLB021098-RA       152 PNRSVTALHK SRCGKSHCFD KIYCCQLKDE -EEDRSSEEI KQKIMENSLT NLATLANMLG YAYPLGARGI NVGEDFHMEG NSWVANKQDF NG-KECRIPG CNERLTIEYK NWSLAVKDII YGDKVIDNLS PEAIHSGVEY NHMNTSSTKT FEFSKIISET IVHSITSSFT 319 BGLB021098-RA
BB02-B23            152 PNRSVTALHK SRCGKSHCFD KIYCCQLKDE -EEDRSSEEI KQKIMENSLT NLATLANMLG YAYPLGARGI NVGEDFHMEG NSWVANKQDF NG-KECRIPG CNERLTIEYK NWSLAVKDII YGDKVIDNLS PEAIHSGVEY NHMNTSSTKT FEFSKIISET IVHSITSSFT 319 BB02-B23

                               350        360        370        380        390        400        410        420        430        440        450        460        470        480        490        500        510
                        ....|....| ....|....| ....|....| ....|....| ....|....| ....|....| ....|....| ....|....| ....|....| ....|....| ....|....| ....|....| ....|....| ....|....| ....|....| ....|....| ....|....|
B1-Antibody_PEP     15  ---------- ---------- ---------- ---------- ---------- ---------- ---------- ---------- ---------- ---------- ---------- ---------- ---------- ---------- ---------- ---------- ---------- 15 B1-Antibody_PEP
xgBioGlab47.1-B1    330 NSHELGVE-- ----LEFEIA ----SVKGKA SYKTRFEYST STTNSKSISE TQGFTKQSSI TLGPMEGAKY EVIMSKSRTT VPYTAIITTK FSTEMKGFLR WEDG----NG NFHQDYRTNS GRP-TFNYRF GDSSVPFYKA LKKQSDNNEG VWMWGMLFQK FPDARRVINR 484 xgBioGlab47.1-B1
BGLB000137          330 NSHELGVE-- ----LEFEIA ----SVKGKA SYKTRFEYST STTNSKSISE TQGFTKQSSI TLGPMEGAKY EVIMSKSRTT VPYTAIITTK FSTEMKGFLR WEDG----NG NFHQDYRTNS GRP-TFNYRF GDSSVPFYKA LKKQSDNNEG VWMWGMLFQK FPDARRVINR 484 BGLB000137
BB02-B1             330 NSHGLGVE-- ----LEFEIA ----SVKGKA SYKTRFEYST STTNSKSISE TQGFTKQSSI TLGPMEGAKY EVIMSKSRTT VPYTAIITTK FSTEMKGFLR WEDG----NG NFHQDYRTNS GRP-TFNYRF GDSSVPFYKA LKKQSDNNEG VWMWGMLFQK FPDARRVTNR 484 BB02-B1
xgBioGlab47.1-B2    330 NSHELGIE-- ----LNFEIA ----SVSGKA SYTTKFEYSK ATTNEKSISQ TTGFTKKSSI TLGPMEGARY EIIMSKSRTT VPYTAIITTK FSTEMRGFLR WEDG----NG NFHQDYRTNS GRP-TYNYRF GDSSVPFYKA LKKQSDNNEG VWMWGMLLQK FPDARQIINR 484 xgBioGlab47.1-B2
BGLB000033          330 NSHELGIE-- ----LNFEIA ----SVSGKA SYTTKFEYSK ATTNEKSISQ TAGFTKKSSI TLGPMEGAKY EIIMSKSRTT VPYTAIITTK FSTEMKGFLR WEDG----NG NFHQDYRTNS GRP-TYNYRF GDSSVPFYKA LKKQSDNNEG VWMWGMLFQK FPDARRVINR 484 BGLB000033
BB02-B2             330 NSHELGIE-- ----LNFEIA ----SVSGKA SYTTKFEYSK ATTNEKSISQ TAGFTKKSSI TLGPMEGAKY EIIMSKSRTT VPYTAIITTK FSTEMKGFLR WEDG----NG NFHQDYRTNS GRP-TYNYRF GDSSVPFYKA LKKQSDNNEG VWMWGMLFQK FPDARRVINR 484 BB02-B2
xgBioGlab47.1-B3    331 NSHELGIE-- ----LNFEYA ----SVSGKA SYTTKFEYST SSTNTKTLDK TEGFTKQSSI TLGPMEGAKY EVIMSKSRTT VPYTAIITTK FSTEMKGFLR WEDG----NG NFHQQHRTNS GRP-SYNYRF GNANNPFYKD LKKQSDNNEG VWMWGMLFQK FPDARRVINR 485 xgBioGlab47.1-B3
BGLB000003          331 NSHELGIE-- ----LNFEYA ----SVSGKA SYTTKFEYST SSTNTKTLDK TEGFTKQSSI TLGPMEGAKY EVIMSKSRTT VPYTAIITTK FSTEMKGFLR WEDG----NG NFHQQHRTNS GRP-TYNYRF GNANIPFYKD LKKQSDNNEG VWMWGMLFQK FPDARRVINR 485 BGLB000003
BB02-B3             331 NSHELGIE-- ----LNFEYA ----SVSGKA SYTTKFEYST SSTNTKTLDK TEGFTKQSSI TLGPMEGAKY EVIMSKSRTT VPYTAIITTK FSTEMKGFLR WEDG----NG NFHQQHRTNS GRP-SYNYRF GNANNPFYKD LKKQSDNNEG VWMWGMLFQK FPDARRVINR 485 BB02-B3
xgBioGlab47.1-B4    330 NNNDLGIS-- ----LNFDVF G---IARSGL SYTFRYTSST QTTNGKTFSQ TDGFSKSSSI TLGPMEGAKY KVILSKSRTT VPYTAIVTTK FSTVMNGFLR WEDG----DG NFHQIYRTSR DRP-TFSYRF GDASVPFYEA LKKQSDNNEG PWMWGMLFQK FPYARHVINR 485 xgBioGlab47.1-B4
BGLB000104          330 NNNDLGIS-- ----LNFDVF G---IARSGL SYTFRYTSST QTTNGKTFSQ TDGFSKSSSI TLGPMEGAKY KVILSKSRTT VPYTAIVTTK FSTVMNGFLR WEDG----DG NFHQIYRTSR DRP-TFSYRF GDASVPFYEA LKKQSDNNEG P--------- ----KKII-- 470 BGLB000104
BB02-B4             330 NNNDLGIS-- ----LNFDVF G---IARSGL SYTFRYTSST QTTNGKTFSQ TDGFSKSSSI TLGPMEGAKY KVILSKSRTT VPYTAIVTTK FSTVMNGFLR WEDG----DG NFHQIYRTSR DRP-TFSYRF GDASVPFYEA LXKQSDNNEG PWMWGMLFQK FPHARHVINR 485 BB02-B4
BGLB000002          330 NSHELGIS-- ----LNFDIG ----IVNGGT SYTFKYESSK TTTNEKSVSQ TAGFTKT--- -LGPGEAAKY KVILSKSRTT VPYTAIVMTK FSTEMKGFLR WEDG----NG NFHQMYRTNR DRP-TFNYRF GDASFSFYEA LKKQSANREG PWMWGMLFQK FPNARHVIDR 480 BGLB000002
BB02-B5             330 NSHELGIS-- ----LNFDIG ----IVNGGT SYTFKYESSK TTTNEKSVSQ TAGFTKT--- -LGPGEAAKY KVILSKSRTT VPYTAIVMTK FSTEMKGFLR WEDG----NG NFHQMYRTNR DRP-TFNYRF GDASFSFYEA LKKQSANREG PWMWGMLFQK FPNARHVIDR 480 BB02-B5
xgBioGlab47.1-B6    323 NSHELGIS-- ----LNFDIG ----VVSGGT SYTFKYESSK TTTKENSVSQ TAGFTKTSSI TLGPGEAAKY KVILSKSRTT IPYTAVVTTK VSTEMKGFLR WEDG----NG NFHQIYRTNR NRP-TFNYRF GDASVSFFEA LKKQSVNKEG PWMWGMLFQK FPIARHVIDR 477 xgBioGlab47.1-B6
BGLB000004          229 NSHELGIS-- ----LNFDIG ----VVSGGT SYTFKYESSK TTTKENSVSQ TAGFTKTSSI TLGPGEAAKY KVILSKSRTT IPYTAVVTTK VSTEMKGFLR WEDG----NG NFHQIYRTNR NRP-TFNYRF GDASVSFFEA LKKQSVNKEG PWMWGMLFQK FPIARHVIDR 383 BGLB000004
BB02-B6             324 NSHELGIS-- ----LNFDIG ----IVNGGT SYTFKYESSK TTTNEKSVSQ TAGFTKTSSI TLGPGEAAKY KVILSKSRTT VPYTAIVMTK FSTEMKGFLR WEDG----NG NFHQMYRTNR DRP-TFNYRF GDASFSFYEA LKKQSANREG PWMWGMLFQK FPNARHVIDR 478 BB02-B6
xgBioGlab47.1-B7    330 NSHELGIS-- ----LNFDIG ----IVSGGT SYTFKYESST TTTNEQSISQ TAGFTKTSKI ELGPMEGAKY RVILSKSRTT IPYTAVVTTK FSTEMKGFLR WEDG----DG NFHQMYRTNR NRP-TFNYRF GDASVPFYEA LKKQSDRNEG PWMWGMLFQK FPNARHVINR 484 xgBioGlab47.1-B7
BGLB000119          330 NSHELGIS-- ----LNFDIG ----IVSGGT SYTFKYESST TTTNEQSISQ TAGFTKTSKI ELGPMEGAKY RVILSKSRTT IPYTAVVTTK FSTEMKGFLR WEDG----DG NFHQMYRTNR NRP-TFNYRF GDASVPFYEA LKKQSDRNEG PWMWGMLFQK FPNARHVINR 484 BGLB000119
BB02-B7             330 NSHELGIS-- ----LNFDIG ----IVSGGT SYTFKYESST TTTNEQSISQ TEGFTKTSKI ELGPMEGAKY RVILSKSRTT IPYTAVVTTK FSTEMNGFLR WEDG----DG NFHQIYRTSR DRP-TFNYRF GDTSVPFYEA LKKQSDRNEG PWMWGMLFQK FPNARHVINR 484 BB02-B7
xgBioGlab47.1-B8    329 TSQETSFK-- ----ISFNVN AVFGSASSST TYTTKYENSF STTNEKSISN TNSFSKQSSI TLGPMQAAKY NVVLNKIRTT VPYTAVVITK FSTEFKGFLR WSDG----DG NFHSKFTYSH DRP-AFNYLF GAETTPFYSA LKRQSDSRSE PWLWNLMLQK YPDARRIINR 487 xgBioGlab47.1-B8
BGLB000016          329 TSQETSFQ-- ----ISFNVN AVFGSASSST TYTTKYENSF STTKEKSISN TNSFSKQSSI TLGPMQAAKY NVVLNKIRTT VPYTAVVITK FSTEFKGFLR WSDG----DG NFHSKFTYSH DRP-AFNYRF GTETTPFYSA LKRQSDSRSE PWLWNLMLQK YPDARRIINR 487 BGLB000016
BB02-B8             329 TSQETSFQ-- ----ISFNVN AVFGSASSST TYTTKYENSF STTNEKSISN TNSFSKQSSI TLGPMQAAKY NVVLNKIRTT VPYTAVVITK FSTEFKGFLR WSDG----DG NFHSKFTYSH DRP-AFNYRF GTETTPFYSA LKRQSDSRSE PWLWNLMLQK YPDARRIINR 487 BB02-B8
xgBioGlab47.1-B9    320 ETHNFGVN-- ----FKYAAY ----GASAQL YYDFLYKTSK TTVTNNNTED VKTFTVNIEK TQEPHTVKKW KLMLSKSRTT LPYTATIRVK FSVDFQGFLR WGGGRNGQDT NYHHQYRGSE DRP-SFHYQF GDKSVPFYEA LKKQSNKNSL PWEWFNIKSK YPVAQALIDD 478 xgBioGlab47.1-B9
BGLB000044          320 ETHNFGVN-- ----FKYAAY ----GASAQL YYDFLYKTSK TTVTNNNIED VKTFTVNIEK TQEPHTVKKW KLMLSKSRTT LPYTATIRVK FSVDFQGFLR WGGGRNGQDT NYHHQYRGSE DRP-SFHYQF GDKSVPFYEA LKEQSNKNSL PWEWFNIKSK YPVAQALIDD 478 BGLB000044
BB02-B9             320 ETHNFGVN-- ----FKYAAY ----GASAQL YYDFLYKTSK TTVTNNNIED VKTFTVNIEK TQEPHTVKKW KLMLSKSRTT LPYTATIRVK FSVDFQGFLR WGGGRNGQDT NYHHQYRGSE DRP-SFHYQF GDKSVPFYEA LKEQSNKNSL PWEWFNIKSK YPVAQALIDD 478 BB02-B9
xgBioGlab47.1-B10   285 NRIDLGLSVT VQIPLMFPQS ----SLSSTS TFTTSFEQSS SNTKDQSTAN SKTFKKTIEK SIAPFSAAEY EVILHKTRTT VPYTATIIAK FSTEFRGFLR WDGGNDSPTS NYHYQFKGSE DRP-TFNYRF GDDSEAFYSA LKKEIITRSK PWLWKDIITN YPDTQHLINR 449 xgBioGlab47.1-B10
BGLB000056          319 NRIDLGLSVT VQIPLMFPQS ----SLSSTS TFTTSFEQSS SNTKDQSTAN SKTFKKTIEK SIAPFSAAEY EVILHKTRTT VPYTATIIAK FSTEFRGFLR WDGGNDSPTS NYHYQFKGSE DRP-TFNYRF GDDSEAFYSA LKKEIITRSK PWLWKDIITN YPDTQHLINR 483 BGLB000056
BB02-B10            319 NRIDLGLSVT VQIPLMFPQS ----SLSSTS TFTTSFEQSS SNTKDQSTAN SKTFKKTIEK SIAPFSAAEY EVILHKTRTT VPYTATIIAK FSTEFRGFLR WDGGNDSPTS NYHYQFKGSE DRP-TFNYRF GDDSEAFYSA LKKEIITRSK PWLWKDIITN YPDTQHLINR 483 BB02-B10
xgBioGlab47.1-B11   327 TSLELSFS-- ----VEVGLK H---LAKASR TLKIGFDYSK SKTETKSNTK TDVLKATTTI SIPPRSAAKC TVIVSKARTT IPYTAVVIAR FNVGFRGFLR WGQGYDSPST NYHSQHKGSG ARP-SVPYTF GDQSEAFYTA LKRQSESQAW PWLWNEMLRN YPSVRHVINR 486 xgBioGlab47.1-B11
BGLB000108          145 TSLELSFS-- ----VEVGLK H---LAKASR TLKIGFDYSK SKTEKKSNTT TDVLKATTTI SIPPRSASKC TVIVSKARTT IPYTAVVIAR FNVGFRGFLR WGQGYDSPST NYHSQHKGSG ARP-SVPYTF GDQSEAFYTA LKRQSESQAW PWLWNEMLRN YPSVRHVINR 304 BGLB000108
BB02-B11            327 TSLELSFS-- ----VEVGLK H---LAKASR TLKIGFDYSK SKTETKSSTK TDVLKATTTI SIPPRSAAKC TVIVSKARTT IPYTAVVIAR FNVGFRGFLR WGQGYDSPST NYHSQHKGSG ARP-SVPYTF GDQSEAFYTA LKRQSESQAW PWLWNEMLRN YPSVRHVINR 486 BB02-B11
xgBioGlab47.1-B12   325 TSLELSFS-- ----FELGVK D---IGKASS TFKVAFEYSQ STTKKNSQTT SHSLKTTSTQ TIPPHSAAEY TIKMSKTRTT IPYTAVIIAK FSVGLRGFLR FGQGFSSPTT NYHFHHKGSD EEQ-SVPYTF GSQSEAFYTA LKRESETQSK PWLWNDMFKN HPSVRHVINK 484 xgBioGlab47.1-B12
BGLB021232-RA       325 TSLELSFS-- ----LEFGFK E---TGKASS TFKVAFEYSQ STTKKNSQTT SHTLKTTSTQ TIPPHSAAEY TIKMSKTRTT IPYTAVIIAK FSVGLRGFLR FGQGFSSPTT NYHFHHKGSD EEQ-SVPYTF GSQSEAFYTA LKRESETQSK PWLWNDMFKN HPSVRHVINK 484 BGLB021232-RA
BB02-B12            325 TSLELSFS-- ----LEFGFK E---TGKASS TFKVAFEYSQ STTKKNSQTT SHTLKTTSTQ TIPPHSAAEY TIKMSKTRTT IPYTAVIIAK FSVGLRGFLR FGQGFSSPTT NYHFHHKGSD EEQ-SIPYTF GSQSEAFYTA LKRESETQSK PWLWNDMFKN HPSVRHVINK 484 BB02-B12
xgBioGlab47.1-B13   329 ISVGMSFS-- ----LEFQIN K---LTQASG TFRTAFEYSR SSTNTNSQMR TDTLRTSSTQ TIPPRSAARY SVMVGKTRTT IPYTAVIIAK FNVRFRGFLR WGGGFSGSTK NYHYQHKGSG DRP-SVPYTF GDQSQPFYTA LKRESESQAR PWLWNEMTNR YPSVRHEISR 488 xgBioGlab47.1-B13
BGLB000122          329 ISVGMSFS-- ----LEFQIN K---LTQASE TFRTAFEYSR SSTNTNSQMR TDTLRTSSTQ TIPPRSAARY SVMVGKTRTT IPYTAVIIAK FNVRFRGFLR WGGGFSGSTK NYHYQHKGSG DRP-SVPYTF GDQSQ----- ---------- ---------- ---------- 453 BGLB000122
BB02-B13            322 ISVGMSFS-- ----LEFQIN K---LTQASE TFRTAFEYSR SSTNTNSRMR TDTLRTSSTQ TIPPRSAARY SVMVGKTRTT IPYTAVIIAK FNVRFRGFLR WGGGFSGSMT NYHYQHKGSG DRP-SVPYTF GDQSQPFYTA LKRESESQAR PWLWNEMTNR YPSVRHEISR 481 BB02-B13
BGLB000086          127 LSNEMSFS-- ----LAFGIK T---LGQASV TYRTAFDYSS SSTDSNSQMR SDTLRTSTTL TIPSRSAAKY TVMVGKTRTT IPYTAVIIAK FNVKLTGFLR WGGGFGGSST NYHYQYRGSH DRP-SLPYTF GDQSEPFYTA LKRESESQAR HWLWNEMTNR YPSVRHEISR 286 BGLB000086
BB02-B14            329 LSNEMSFS-- ----LEFGIK T---LGQASV TYRTAFDYSS SSTDSNSQMR SDTLRTSTTL TIPSRSAAKY TVMVGKTRTT IPYTAVIIAK FNVKLTGFLR WGGGFGGLST NYHYQYRGSH DRP-SLPYTF GDQSEPFYTA LKRESESQAR PWLWNEMTNR YPSVRHEISR 488 BB02-B14
xgBioGlab47.1-B15   327 NSHEVSVS-- ----LNFDLG D---VLKISL SYKTAFEYSR STTNKNSQMK SDTLKISTTQ TIPPRSAAKY TVMVGKTRTT IPYTAVIIAK FNVKFRGFLR WGKGYGSPIT NYHHQHKGSG DRP-TVPYTF GDQSEAFYTA LKRESETQAM PWLWNEMIKN YPSARQLINR 486 xgBioGlab47.1-B15
BGLB000076          327 NSHEVSVS-- ----LNFDLG D---VLKISL SYKTAFEYSR STTNKNSQMK SDTLKISTTQ TIPPRSAAKY TVMVGKTRTT IPYTAVIIAK FNVKFRGFLR WGKGYGSPIT NYHYKHKGSG DRP-TVPYTF GDQSEAFYTA LKRESETQAM PWLWNEMIKN YPSARQLINR 486 BGLB000076
BB02-B15            327 NSHEVSVS-- ----LNFDLG D---VLKISL SYKTAFEYSR STTNKNSQMK SDTLKISTTQ TIPPRSAAKY TVMVGKTRTT IPYTAVIIAK FNVKFRGFLR WGKGYGSPIT NYHYKHKGSG DRP-TVPYTF GDQSEAFYTA LKRESETQSM PWLWNEMIKN YPSARQLINR 486 BB02-B15
```

```
xgBioGlab47.1-B16   327 NSHEVSVS-- ----LNFNLF N---ILQTSL SYKTTFENSR STTDNNSQMK SDTLKISTTQ TIPPRSAAKF TVLVGKTRTT IPYTAVIIAR FNVKFRGFLR WGKGFGSATT NYHHQHKGSG DRP-TVPYTF GDKSEAFYTA LKRESETQAM PWLWNEMIKN YPSARQLINR 486  xgBioGlab47.1-B16
BGLB000125-RA       327 NSHEFSVS-- ----LNFNLF N---ILQTSL SYKTAFENSR STTDTNSQMK SDTLKISTTQ TIPPRSAAKF TVLVGKTRTT IPYTAVIIAK FNVKFRGFLR WGKGFGSPTT NYHHQHKGSG DRP-TVPYTF GDKSEAFYTA LKRESETQAM PWLWNEMIKN YPSARQLINR 486  BGLB000125-RA
BB02-B16            327 NSHEFSVS-- ----LNFNLF N---ILQTSL SYKTAFENSR STTDTNSQMK SDTLKISTTQ TIPPRSAAKF TVLVGKTRTT IPYTAVIIAK FNVKFRGFLR WGKGFGSPTT NYHHQHKGSG DRP-TVPYTF GDKSEAFYTA LKRESETQSM PWLWNEMIKN YPSARQLINR 486  BB02-B16
xgBioGlab47.1-B17   329 NSLEYSVS-- ----LNFDLF D---ILKISA TYKTTFEYTS STTNENSLMK ASTFKVSTTQ TIPRRSAVKY TVVLGKTRTT VPYTAVIIAK FNVKFRGFLR WGGGSSSTTT NYHYLHKGSG DRP-SIPYTF GDQSEAFYAA LKRQSEHQAR PWLWNEMINN YPSARHLINR 488  xgBioGlab47.1-B17
BGLB000064          329 NCLEYSVS-- ----LNFDLF D---ILKISA TYKTTFEYTS STTNENSLMK ASTFKVSTTQ TIPPRSAVKY TVVLGKTRTT VPYTAVIIAK FNVKFRGFLR WGGGSSSTTT NYHYLHKGSG DRP-SIPYTF GDQSEAFYAA LKRQSEHQAR PWLWNEMINN YPSARHLINR 488  BGLB000064
BB02-B17            329 NSLEYSVS-- ----LNFDLF D---ILKISA TYKTTFEYTS STTNENSQMK ASTFKVSTTQ TIPPRSAVKY TVVLGKTRTT VPYTAVIIAK FNVKFRGFLR WGGGSSSTTT NYHYLHKGSG DRP-SIPYTF GDQSEAFYAA LKRQSEHQAR PWLWNEMINN YPSARHLINR 488  BB02-B17
xgBioGlab47.1-B18   329 TSLQLSFS-- ----LQAATP G---AAKVSA GVNVAFDYSK SDTDTSTEVK SQTTKITTVQ TIPPHSAANY TVKVLKTRTT NPYTAVIIAK FNVRFRGFLR WGGGYGNPLT NYHYQYRGSG ARP-SFPYTF GSQSDAFYKA LKRQNITKSR PWMWNDMIKN YPSVLHVFNR 488  xgBioGlab47.1-B18
BGLB000010          329 TSLELSFS-- ----LEASKI FKTVAGKASA AFKVSFDLSE SDTDTSTEVK TETTKITTTQ TIPPHSAANY SVMVLKTRTT IPYTAVIIAK FNVRFRGFLR WGGGYGNPLT NYHYQYRGSG ARP-SFPYTF GSQSRAFYKA LKRQNITKSR PWMWNDMIKN YPSVLNVINR 491  BGLB000010
BB02-B18            329 TSLELSFS-- ----LEASKI FKTVAGKASA AFKVSFDLSE SDTDTSTEVK TETTKITTTQ TIPPHSAANY SVMVLKTRTT IPYTAVIIAK FNVRFRGFLR WGGGYGNPLT NYHYQYRGSG ARP-SFPYTF GSQSRAFYKA LKRQNITKSR PWMWNDMIKN YPSVLNVINR 491  BB02-B18
BGLB000001-RA       329 TSFEYTVS-- ----MEVDAK F---ISKIKG SIMMAFENSE LYKTEMGSKN GEELVDRINY SIPAQTFAKY KVFLSKIRTT TPYTAIFIAK FNVRFTGMLR YGGGYASKMT NFHYQYKGST ERP-SFTYTF GDDSDAFYTA LKLQSRNRAM PWLWKDIFKN SDRIRDIINS 488  BGLB000001-RA
BB02-B19            329 NNVDYSVS-- ----LEYDTK F---FGKITG SIKMAFEDSE IKTAEEGSKN ADQLKKVISY SIPAQTFAKY KVFLSKIRTT TPYTAIFIAK FNVRFTGMLR YGGGYASKMT NFHYQYKGST ERP-SFTYTF GDDSDAFYTA LKHQSRNRAM PWLWKDIFKN SDRIRDIINS 488  BB02-B19
xgBioGlab47.1-B20   331 DNPEYISL-- ----MEYDPK Y---FGNIAR SIKLAAEGSR SNTAGEGKK- --VLNNAFMY SIPPRTLAKF KFFFNKVRTT TPYTANLVAK FNVRFTGILR P--------H NLHHQYRGSQ ERP-KIIHTF GDESETFYTD LRRQKRINAS PWLWYDMH-- -DYYHSVVND 476  xgBioGlab47.1-B20
BGLB000202          285 DNPEYISL-- ----LEYDPK Y---FGNIAR SIKLAAEGSR SNTAGEGKK- --VLNNAFMY SIPARTLAKF KFFLNKVRTT TPYTANLVAK FNVRFTGILR P--------H NLHHQYRGSQ ERP-KIIHTF GDESETFYTD LRRQKRINAS PWLWYDMH-- -DYYHSVVND 430  BGLB000202
BB02-B20            331 DNPEYISL-- ----MEYDPK Y---FGNIAR SIKLAAEGSR SNTAGEGKK- --VLNNGFMY SIPPRTLAKF KFFLNKVRTT TPYTANLVAK FNVRFTGILR P--------H NLHHQYRGSQ ERP-KIIHTF GDESETFYTD LRRQKRINAS PWLWYDMH-- -DYYHSVVND 476  BB02-B20
xgBioGlab47.1-B21   328 NNEKLSVS-- ----LEFGTN L---FSKVQM PFKTGFQYSE LIITGNNEAE RTTLHDTLEH NIPPNTSAQY KVLLKKIRTT IPYTAVIIAN FSVRYRGFLG RGNGFDNEKT NFHINYREST DRP-GVPYTF GSKSEAFYTA LSRQIASKEE PWMWEEILRK DPSLGEHIKK 487  xgBioGlab47.1-B21
BGLB000109          328 NNEKLSVS-- ----LEFGTN L---FSKVQM PFKTGFKYSE LIITGNNEAE RSTLHDTLEH NIPPNTSAQY KVLLKKIRTT IPYTAVIIAN FSVRYR---- ---------- ---------- ---------- ---------- ---------- ---------- ---------- 414  BGLB000109
BB02-B21            328 NNEKLSVS-- ----LEFGTN L---FSKVQM PFKTGFQYSE LIITGNNEAE RTTLHDTLEH NIPPNTSAQY KVLLKKIRTT IPYTAVIIAN FSVRYRGFLG RGNGFDSEKT NFHINYREST DRP-GVPYTF GSKSEAFYTA LSRQIASKEE PWMWEEILRK DPSLGEHIKK 487  BB02-B21
xgBioGlab47.1-B22   330 NSAEMSLS-- ----LDFGVK S---LGSVSM SFTFRVEYSS STTNQKQHSN SINFRVATNK VIPPKSYAKY KIMVSKSRTT IPYTAIIVAK FSVTFDGYLS TDDENSNPNY NFHYKY-ETP KKFWRVSHAF GDHFEPFYEA IKRQYTSSAK PWLWMEMTKN NPPVTRIIDV 489  xgBioGlab47.1-B22
BGLB028593-RA       330 NSAEMSLS-- ----LDFGIK S---LGSVSM SFTFRVEYSS STTNQKQHSN SINFRVATNK VIPPKSYAKY KIMVSKSRTT IPYTAIIVAK FSVAFDGYLS TDDENSNPNY NFHYKY-ETP KKFWRVSHAF GDHLEPFYEA IKRQYTSSAK PWLWMEMTKN NPPVTRIIDV 489  BGLB028593-RA
BB02-B22            330 NSAEMSLS-- ----LDFGIK S---LGSVSM SFTFRVEYSS STTNQKQHSN SINFRVATNK VIPPKSYAKY KIMVSKSRTT IPYTAIIVAK FSVAFDGYLS TDDENSNPNY NFHYKY-ETP KKFWRVSHAF GDHLEPFYEA IKRQYTSSAK PWLWMEMTKN NPPVTRIIDV 489  BB02-B22
xgBioGlab47.1-B23   320 TSAEMSAS-- ----LDFGFK S---FG-GSI SLTLGIEHST TSSSEDSNMK SVLYKASTTH TIPPQSAAKY SAVVSKVRTT IPYTAVIAAR FSVRFTGYLR VREGLEHPNF NFHYRYSNIL DKNKWIDFTF GDTSEPFHAA LQRANQTKCL PWLWNEIKQN YNDSIPLINE 479  xgBioGlab47.1-B23
BGLB021098-RA       320 TSAEMSAS-- ----LDFGFK S---FG-GSI SLTLGIEHST TSSSEDSNMK SVLYKASTTH TIPPQSAAKY SAVVSKVRTT IPYTAVIAAR FSVRFTGYLR VREGLEHPNF NFHYRYSNIL DKNKWIDFTF GDTSEPFHAA LQRANQTKCL PWLWNEIKQN YNDSIPLINE 479  BGLB021098-RA
BB02-B23            320 TSAEMSAS-- ----LDFGFK S---FG-GSI SLTLGIEHST TSSSEDSNMK SVLYKASTTH TIPPQSAAKY SAVVSKVRTT IPYTAVIAAR FSVRFTGYLR VREGLEHPNF NFHYRYSNIL DKNKWIDFTF GDTSEPFHAA LQRANQTKCL PWLWNEIKQN YNDSIPLINE 479  BB02-B23

                                   520        530        540        550        560        570        580        590        600        610
                        ....|....| ....|....| ....|....| ....|....| ....|....| ....|....| ....|....| ....|....| ....|....| ....|....| ....|.

B1-Antibody_PEP      15 ---------- ---------- ---------- ---------- ---------- ---------- ---------- ---------- ---------- ---------- ------ 15   B1-Antibody_PEP
xgBioGlab47.1-B1    484 --LTDETQYQ FTLTGKLEKV EGTSVNVKWE KMKL---NRR DVSGNDAPG- ---SNITTYI AASGPADKPA VVE------- YPKVNLNNKE PFKPIEIPVT EVKV-- 572  xgBioGlab47.1-B1
BGLB000137          484 --LTDETQYQ FTLTGKLEKV EGTSVNVKWE KMKL---NRR DVSGNDAPG- ---SNITTYI AASGPADKPA VVE------- YPKVNLNNKE PFKPIEISVT EVKV-- 572  BGLB000137
BB02-B1             484 --LTDETQYQ FTLAGKLEKV EGTSVNVKWE KMKL---NRR DVSGNDEPG- ---SNITTYI AASGPADKPA VVE------- YPKVNLNNKE PFKPIEIPVT EVKV-- 572  BB02-B1
xgBioGlab47.1-B2    484 --LTDETQYQ FTLTGKLEKV EGTSVNVKWE KMKL---NRR DVSGNDAPG- ---RNITTYI AASGPADKPA VVE------- YPKVNLNNKE PFKPIEIPVT EVKV-- 572  xgBioGlab47.1-B2
BGLB000033          484 --LTDETQYQ FTLTGKLEKV EGTSVNVKWE KIKL---NRR DVSGNDAPG- ---SNITTYI AASGPADKPA VVE------- YPKVNLNNKE PFKPIEIPVT EVKV-- 572  BGLB000033
BB02-B2             484 --LTDETQYQ FTLTGKLEKV EGTSVNVKWE KMKL---NRR DVSGNDEPG- ---RNITTYI AASGPADKPA VVE------- YPKVNLNNKE PFKPIEIPVT EVKV-- 572  BB02-B2
xgBioGlab47.1-B3    485 --LTDETQYQ FTLTGKLEKV EGTSINVKWQ KVKL---NRR DVSNDKVSA- ---RNITTYI AKSGPADKPA VVE------- YPKVNLNNQE SFKPVQIPVT EVKV-- 573  xgBioGlab47.1-B3
BGLB000003          485 --LTDETQYQ FTLTGKLEQV LM-------- ---------- ---------- ---------- ---------- ---------- ---------- ---------- ------ 505  BGLB000003
BB02-B3             485 --LTDETQYQ FTLTGKLEKV EGTSINVKWQ KVKL---NRR DVSNDKVSA- ---RNITTYI AKSGPADKPA VVE------- YPKVNLNNQE SFKPVQIPVT EVKVSR 575  BB02-B3
xgBioGlab47.1-B4    485 --LTDESQYQ FTLTGKLEKV EGTHVDVEWE KIPL---SRR DVSGDEAPA- ---SNITTYI ATSGSADKPA SVE------- YPKVELKNKE PFEPIQIPVS NIKV-- 573  xgBioGlab47.1-B4
BGLB000104          470 ---------- ---------- ---------- ---------- ---------- ---------- ---------- ---------- ---------- ---------- ------ 470  BGLB000104
BB02-B4             485 --LTDESQYQ FTLTGKLEKV EGTHVDVXWE KIPL---SRR DVSSDEVPA- ---SNITTYI ATSGPADKPA SVE------- YPKVELKNKE PFEPIRIPVS NIKV-- 573  BB02-B4
BGLB000002          480 --LTDESQYQ FTLTGKLEKV EGTHIYIKWE KMPQ---GRR DVSRDEEPA- ---SNITTYI AAPGPADKPA SVE------- YPKVDLKNKV PFEPFQIPIT SV---- 566  BGLB000002
BB02-B5             480 --LTDESQYQ FTLTGKLEKV EGTHIYIKWE KMPQ---GRR DVSRDEEPA- ---SNITTYI AAPGPADKPA SVE------- YPKVDLKNKV PFEPFQIPIT SV---- 566  BB02-B5
xgBioGlab47.1-B6    477 --LTDESQYQ FSLTGKLEKV EGTHIYIEWE KIPQ---GLR DVSRDEAPA- ---SNITTYI AAPGPADNPA IVE------- YPKVDIKSKV PFEPFQIPIT SV---- 563  xgBioGlab47.1-B6
BGLB000004          383 --LTDESQYQ FSLTGKLEKV EGTHIYIEWE KIPQ---GLR DVSRDEAPA- ---SNITTYI AAPGPADNPA IVE------- YPKVDIKSKV PFEPFQIPIT SV---- 469  BGLB000004
BB02-B6             478 --LTDESQYQ FSLTGKLEKV EGTHIYIEWE KISQ---GRR DVSLDEAPA- ---SNITTYI AAPGPADKPA IVE------- YPKVELKNKE PFEPIQIPVS NIKV-- 566  BB02-B6
xgBioGlab47.1-B7    484 --LTDEPQYQ FTLTGKLEKV EGTYINVEWE KIPL---SRR DVSGDEVPA- ---SNSTTYI ATSGPADKPA SVE------- YPKVELKNNE PFEPIQIPVR NV---- 570  xgBioGlab47.1-B7
BGLB000119          484 --LTDEPQYQ FTLTGKLEKV EGTYINVEWE KIPL---SRR DVSGDEVPA- ---SNSTTYI ATSGPADKPA SVE------- YPKVELKNNE PFEPIQIPVR NVKV-- 572  BGLB000119
BB02-B7             484 --LTDETQYQ FTLTGKLEKV EGTYINVEWE KIPL---NRR NVNRDEAPA- ---SNITTYI ATSGPADKPA SVE------- YPKVELKNNE PFEPIQIPVR NVKV-- 572  BB02-B7
xgBioGlab47.1-B8    487 --LTDETQYE FALNGRLEFV EGTDASVVWE KVNI---TKR DVLVNDDTT- ---KSTHPYV AKSGPLDKPA EVQ------- YPEVQISNKE PVAVSVIPVN ------ 571  xgBioGlab47.1-B8
BGLB000016          487 --LTDETQYE FTLNGRLEFV EGTDASVVWE KVNI---TKR DVLVNDDTT- ---KSTHPYV AKSGPLDKPA EVQ------- YPEVQIGNKE PVVVSVIPVN ------ 571  BGLB000016
BB02-B8             487 --LTDETQYE FALNGRLEFV EGTDASVVWE KVNI---TKR DVLVNDDTT- ---KSTHPYV AKSGPLDKPA EVQ------- YPEVQISNKE PVVVSVIPVN ------ 571  BB02-B8
xgBioGlab47.1-B9    478 --LVNEKHYQ FTLNGEFEDI MGTSVDVVWG PVTK---RKR ELSPLKFSN- --PIFRENRI AELGPHDLPV HVE------- KPNIFLDTAK ICEFDVPVNI SYLV-- 567  xgBioGlab47.1-B9
BGLB000044          478 --LVNEKHYQ FTLNGEFEDI MGTSVDVVWG PVTK---RKR ELSPLKFSN- --PIFRENRI AELGPHDLPV HVD------- KPNIFLDTAK ICEFDVPVNI SYLV-- 567  BGLB000044
BB02-B9             478 --LVNEKHYQ FTLNGEFEDI MGTSVDVVWG PVTK---RKR ELSPLKFSN- --PIFRENRI AELGPHDLPV HVD------- KPNIFLDTAK ICEFDVPVNI SYLV-- 567  BB02-B9
xgBioGlab47.1-B10   449 --LIDESQYE FTLSGTFEHV AGKRIDVLWN KVSM---TKR EAHFGSRSRR QCNIIKGTHY ARPGPNDAPA IVK------- YPEVTLVNSE PYR-ESPHYV ENKSA- 541  xgBioGlab47.1-B10
BGLB000056          483 --LIDESQYE FTLSGTFEHV AGKRIDVLWN KVSM---IKR EAHFGSRSRR QCNIIKGTHY ARPGPNDAPA IVK------- YPEVTLVNSE PYR-EGPHYV ENKSA- 575  BGLB000056
BB02-B10            483 --LIDESQYE FTLSGTFEHV AGKRIDVLWN KVSM---TKR EAHFGSRSRR QCNIIKGTHY ARPGPNDAPA IVK------- YPEVTLVNSE PYR-EGPHYV ENKSA- 575  BB02-B10
xgBioGlab47.1-B11   486 --LTDESQYE ITLNGKLEHV SGTKIDIKWE TRELNKNFNR RSETDDGKAN TTQLNNGTFF ATVGPNDKPV DVK------- YPEVELVNAE PFELDILPAN QESS-- 581  xgBioGlab47.1-B11
BGLB000108          304 --LTDESQYE ITLNGKLEHV SGTKIDIKWE TRELNKNFNR RSETDDSKAN TPQLNNGTFF ATVGPNDKPV DVK------- YPEVELVNAE PFELDILPAN QESS-- 399  BGLB000108
BB02-B11            486 --LTDESQYE ITLNGKLEHV SGTKIDIKWE TRELNKNFNR RSETDDSKAN TPQLNNGTFF ATVGPNDKPV DVK------- YPEVELVNAE PFELDILPAN QESS-- 581  BB02-B11
xgBioGlab47.1-B12   484 --LTNESQYE FTLNGKLEHV AGTKIDITWK KVEL-KDLNR RSVLDESKAD TPGPNNMTIF ATVGPKDKPV DVE------- YPEVHLGNAD QVKLETGPVN QDSS-- 578  xgBioGlab47.1-B12
BGLB021232-RA       484 --LTNESQYE FTLNGKLEHV AGTKIDITWK KVEL-KDLNR RSVLDESKAD TPSPNNITIF ATVGPKDKPV DVE------- YPEVHLGNAD QVKLETGPVN QNSS-- 578  BGLB021232-RA
BB02-B12            484 --LTNESQYE FTLNGKLEHV AGTKIDITWK KVEL-KDLNR RSVLDESKAD TPGPNNITIF ATVGPKDKPV DVE------- YPEVHLGNAD QVKLETGPVN QNSS-- 578  BB02-B12
xgBioGlab47.1-B13   488 --LTDESQYQ FTLNGKLEHV AGTKIDVIWE NARL----NR RSVPDESEPS TPYFDNSTFF AKVGPNDKPV DVK------- YPEVVLVNAE PFIPETFPVK PESN-- 579  xgBioGlab47.1-B13
BGLB000122          453 ---------- ---------- ---------- ---------- ---------- ---------- ---------- ---------- ---------- ---------- ------ 453  BGLB000122
BB02-B13            481 --LTDESQYQ FTLNGKLEHV AGTKIDVIWE NARL----NR RSVPDESEPS TPYFDNSPFF AKVGPNDKPV DVK------- YPEVVLVNAE PFILETFPVK PESN-- 572  BB02-B13
BGLB000086          286 --LTDESQYQ FTLNGKLEHV AGTKIDVIWE NARL----NR RSVPDESDSS TPYFDNSTFF AKVGPNDKPV DVK------- YPEVVLVNAE PFILETFPVK PESN-- 377  BGLB000086
BB02-B14            488 --LTDESQYQ FTLNGKLEHV AGTKIDVIWE NARL----NR RSVPDESDSS TPYFDNSTFF AKVGPNDKPV DVK------- YPEVVLVNAE PFILETFPVK PESN-- 579  BB02-B14
xgBioGlab47.1-B15   486 --LTNETQYE FTLNGKLQHV AGTKIDVIWE KARL----NR RSIRDKIKSS TPQFDNSTFF ATVGLNDKPV DVK------- YPEVVLVNSE PFQFESIPFD PDSN-- 577  xgBioGlab47.1-B15
BGLB000076          486 --LTNETQYE FTLNGKLQHV AGTKIDVIWE KARL----NR RSIRDKIKSS TPQLDNSTFF ATVGPNDKPV DVK------- YPEVVLVNSE PFQFESIPFD PDSN-- 577  BGLB000076
BB02-B15            486 --LTNETQYE FTLNGKLQHV AGTKIDVIWE KARL----NR RSIRDKIKSS TPQLDNSTFF ATVGLNDKPV DVK------- YPEVVLVNSE PFQFESIPFD PDSN-- 577  BB02-B15
xgBioGlab47.1-B16   486 --LTNETQYE FTLNGKLEHV AGTKIDVIWE NARL----SR RSIRDKIKSS TPQFDNSTFF ATVGPNDKPV DVK------- YPEVVLVNSE PFQLESIPVD PNSI-- 577  xgBioGlab47.1-B16
BGLB000125-RA       486 --LTNETQYE FTLNGKLEHV AGTKIDVIWE KARL----NR RSIRDKIKSS TPQFDNSTFF ATVGPNDKPV DVK------- YPEVVLVNSE PFQLESIPVD PNSI-- 577  BGLB000125-RA
BB02-B16            486 --LTNETQYE FTLNGKLEHV AGTKIDVIWE KARL----NR RSIRDKIKSS TPQFDNSTFF ARVGPKDKPV DVK------- YPEVVLVNSE PFQLESIPVD PNSI-- 577  BB02-B16
xgBioGlab47.1-B17   488 --LTDESQYE FTLSGKLEHV AGTKIDVIWE NARL----NR RSVPDKRKSN ISHLAYSSFF ATVGPKDKSV DVK------- YPKVELANAE LFKQESILLN PNTT-- 579  xgBioGlab47.1-B17
BGLB000064          488 --LTDESQYE FTLSGKLEHV AGTKIDVIWE NARL----NR RSVPDKRKSN ISHLAYSSFF ATVGPKDKSV DVK------- YPKVELANAE LFKQESILLN PNTT-- 579  BGLB000064
BB02-B17            488 --LTDESQYE FTLSGKLEHV AGTKIDVIWE NARL----NR RSVPDKRKSN ISHLAYSSFF ATVGPKDKSV DVK------- YPKVELANAE LFKQESILLN PNTT-- 579  BB02-B17
xgBioGlab47.1-B18   488 --LTDESQYE FTLRGKLEHV ASRNIIVIWN KTRL----NR RSVVDTINST THHIGNITFI ARVGPNDKPV DVK------- YPEVVLDNAE PFKLETIPLN PDFN-- 579  xgBioGlab47.1-B18
BGLB000010          491 --LTNESQYE FTLNGKLEHV AGRNVVVIWN KTRL----NR RSVVDTINST THHIGNITFI ARVGPNDKPV DVK------- YPEVVLDNAE PFKLETIPLN PDFN-- 582  BGLB000010
BB02-B18            491 --LTNESQYE FTLNGKLEHV AGRNVVVIWN KTRL----NR RSVVDTINST THHIGNITFI ARVGPNDKPV DVK------- YPEVVLDNAE PFKLETIPLN PDFN-- 582  BB02-B18
BGLB000001-RA       488 --LTNETKYQ FILNGKFEQV YGKSIHSLVD LTKL----RS PSVRRKRQTE TFYVND-TFI AKAGPDDKPV EVN------- YPEISLQEIS PIELEPILND PDSS-- 578  BGLB000001-RA
BB02-B19            488 --LTNETKYQ FILNGKFEQV YGKSVHSLVD LTKL----RS PSVRRKRQTE TFYVND-TFI AKAGPDDKPV EVN------- YPEISLQEIS PIELEPILND PDSS-- 578  BB02-B19
xgBioGlab47.1-B20   477 PYLANETNYQ LKLRGKFEYV TGKSLHTLVE FKAA----ND ---RRKRETE TYYFNG-TFI AKAGPDDKPV EVN------- YPEISSKGKN RIQLEPIFND PVSS-- 565  xgBioGlab47.1-B20
BGLB000202          431 PYLANETNYQ LKLRGKFEYV TGKSLHTLVE FKAA----ND ---RRKRETE TYYFNG-TFI AKAGPDDKPV EVN------- YPEISSKGKN RIQLEPIFND PVSS-- 519  BGLB000202
BB02-B20            477 PYLANETNYQ LKLRGKFEYV TGKSLHTLVE FKAA----ND ---RRKRETE TYYFNG-TFI AKAGPDDKPV EVN------- YPEISSKGKN RIQLEPIFND PVSS-- 565  BB02-B20
xgBioGlab47.1-B21   487 --LTEENKYK FTLNGKLEHV AGAKFDSMLT LMKL----NS FSGLEENQTE TQYLND-ILI AKEGPKDKPV DRKIPEYVLK YPKVDLDDAN PLKLEPILKD LDYSS- 585  xgBioGlab47.1-B21
BGLB000109          414 ---------- ---------- ---------- ---------- ---------- ---------- ---------- ---------- ---------- ---------- ------ 414  BGLB000109
BB02-B21            487 --LTEENKYK FTLNGKLEHV AGAKFDSMLT LMKL----NS FSGLEENQTE TQYLND-ILI AKEGPKDKPV DRKIPEYVLK YPKVDLDDAN PLKLEPILKD LDYSS- 585  BB02-B21
xgBioGlab47.1-B22   489 --MSREEQYT FTVSGKLERV WGSKIDVVWE KGRN----IR -SVPNEVKFK NICPDR-VFI VDAGPMDTPS IVE------- YPKVNLTNED WDEIEQIPIT TDGI-- 578  xgBioGlab47.1-B22
BGLB028593-RA       489 --MSREEQYT FTVSGKLERV WGSKIDVVWE KGRN----IR -SVPNEVKFE NICPDR-VFI VDAGPMDTPS IVE------- YPKVNLTNED WDEIEQIPIT TDGT-- 578  BGLB028593-RA
BB02-B22            489 --MSREEQYT FTVSGKLERV WGSKIDVVWE KGRN----IR -SVPNEVKFE NICPDR-VFI VDAGPMDTPS IVE------- YPKVNLTNED WDEIEQIPIT TDGT-- 578  BB02-B22
xgBioGlab47.1-B23   479 --LADENQYA FTLSGKLEHI AGSKIDVVWK EVRL----HR RSTYDELGAG NSCPNR-TYI AKTGPSDKPA KVN------- YPKINIPKAK PSEIK--PTL AD---- 565  xgBioGlab47.1-B23
BGLB021098-RA       479 --LADENQYA FTLSGKLEHI AGSKIDVVWK EVRL----HR RSTYDELGAG NSCPNR-TYI AKTGPSDKPA KVN------- YPKINIPKAK PSEIK--PTL AD---- 565  BGLB021098-RA
BB02-B23            479 --LADENQYA FTLSGKLEHI AGSKIDVVWK EVRL----HR RSTYDELGAG NSCPNR-TYI AKTGPSDKPA KVN------- YPKINIPKAK PSEIK--PTL AD---- 565  BB02-B23
```
